# The β_2_Tubulin, Rad50-ATPase and enolase *cis*-regulatory regions mediate male germline expression in *Tribolium castaneum*

**DOI:** 10.1038/s41598-021-97443-9

**Published:** 2021-09-13

**Authors:** Sher Afzal Khan, Emma Jakes, Kevin M. Myles, Zach N. Adelman

**Affiliations:** 1 Department of Ecology and Genetics, Animal Ecology, Uppsala University, Norbyvägen 18 D, 752 36 Uppsala, Sweden; 2 Department of Entomology and Agrilife Research, Texas A & M University, College Station, TX 77843 USA

**Keywords:** Genetic engineering, Molecular biology, Biotechnology, Gene expression

## Abstract

Genetics-based pest management processes, including the sterile insect technique, are an effective method for the control of some pest insects. However, current SIT methods are not directly transferable to many important pest insect species due to the lack of genetic sexing strains. Genome editing is revolutionizing the way we conduct genetics in insects, including in *Tribolium castaneum*, an important genetic model and agricultural pest. We identified orthologues of β_2_Tubulin, Rad50-ATPase and enolase in *T. castaneum.* Using RT-PCR, we confirmed that these genes are predominantly expressed in the testis. *PiggyBac*-based transformation of *T. castaneum cis-*regulatory regions derived from *Tc-β*_*2*_*t*, *Tc-rad50* or *Tc-eno* resulted in EGFP expression specifically in the *T. castaneum* testis. Additionally, we determined that each of these regulatory regions regulates EGFP expression in different cell types of the male gonad. *Cis*-regulatory regions from *Tc-β*_*2*_*t* produced EGFP expression throughout spermatogenesis and also in mature sperms; *Tc-rad50* resulted in expression only in the haploid spermatid, while *Tc-eno* expressed EGFP in late spermatogenesis. In summary, the regulatory *cis*-regions characterized in this study are not only suited to study male gonadal function but could be used for development of transgenic sexing strains that produce one sex in pest control strategies.

## Introduction

Infestation of plant crops by insect pests causes more than 45 billion US dollars in agricultural losses each year^1^, including damage caused by beetles. Insect pest management provides a number of different methods by which crop damage can be reduced. Conventional sterile insect technique (SIT) programs use radiation to induce male sterility in insects prior to releasing them in field^2^. In such genetics-based pest management programs, several approaches are used for the sex separation of insects so that only males are released^3^. For SIT programs for insects such as the mosquito *Aedes aegypti*, male and female sexes can be separated using differences in pupal body size. Some sex-specific phenotypes, for example body size or development rate are also influenced by environmental factors, and thus special care is required for such systems to be used effectively. However, many of these methods are not directly transferable to other important insect species. Irradiation may also cause reduced male mating competitiveness, potentially reducing the efficiency of traditional SIT approaches.

Several genetic approaches have been developed or are in development for the efficient sex separation of insects^3^. Genetically engineered systems for male sterilization through carrying a dominant lethal trait in males is a promising alternative to conventional SIT methods^4,5^. A precision-guided SIT strategy was recently demonstrated in *Drosophila melanogaster* in which complete male sterility was achieved by the directed mutagenesis of Dm-β2t using genome editing with CRISPR/Cas9^6^. A transgenic sperm-marking strain was established by HDR-based genome editing in the pest *Drosophila suzuki*^7^. The development of such a “genetic-sexing” strain (GSS) is an alternative approach for improving the efficiency of SIT that could facilitate the mass scale separation of males and females for new pest species, including coleopterans^8^. However, the development of genetic sexing approaches in a new pest species may require knowledge about gonad differentiation, sex-biased gene expression and/or regulatory elements capable of efficient and conditional heterologous gene expression in the targeted organisms^9–12^. Tissue or stage-specific transgene expression is of particular value in the field of insect biotechnology, with enhancer/promoter elements used to drive the expression of fluorescent proteins or effector molecules in agricultural pests and disease vectors for sexing, monitoring, and reproductive biology studies^7,8,11–14^.

The testis-specific β2-tubulin gene in *D. melanogaster* has been studied in detail to determine its role in spermatogenesis^15,16^. Mutant β2-tubulin disrupts meiosis in the testis, generating impaired sperm and thus producing male sterility^17^. Study of the male gonad in insects is important for understanding the mechanism of how sexual identity impacts the processes of tissue organogenesis to create sexual dimorphism. Differential gene expression and regulation in the gonads is essential for producing either male sperm or female eggs required for sexual reproduction^18^, while variation in gene regulatory networks have been a major driving force in the production of the diverse morphology and phenotypes in different organisms^19^. The characterization of c*is*-regulatory regions is also critical for understanding conditional gene expression, assessing the impact of genetic variation on different phenotypes in evolutionary biology and for controlling transgene expression. Established cis-regulatory regions enable strategies to induce sterility by linking insect regulatory elements to lethal effector genes without compromising mating behavior. For example, Yamamoto et al.^20^ reported that the β2-tubulin promoter from *Anopheles stephensi* has been used to express a pro-apoptotic factor and thus impair male sterility, resulting in normal mating with control females. To breed lines in the lab with genetically-encoded male sterility, the sterility genes can be switched off/on via the tTA system, which has been established in several pests^21,22^. Transgenic male sterile strains can also be used to study reproductive biology and mating behavior, including sperm transfer, storage and sperm competition^12,23,24^.

Coleoptera (Beetles) are the most diverse animal group on earth and contain one fourth of all species described and includes many major pests of crop plants^25^. Coleoptera consists of approximately 380,000 known species, representing ca. 40% of insect diversity^26^, including *T. castaneum*, a model for functional genetics and developmental studies in agricultural pests. *T. castaneum* has a good quality annotated genome^27,28^, large scale RNA interference (RNAi)-based screens^29,30^, as well as efficient transgenesis and genome editing methods^31–33^. In many respects, *T. castaneum* is more representative of insects than *D. melanogaster*^34^. However, there have been very few reports about the stage-specific transgene expression in coleopteran insects, particularly in *T. castaneum*^32^. In this study we describe the identification and use of *cis*-regulatory regions derived from three genes (β_2_Tubulin, rad50 and enolase) expressed predominantly in the testis of *T. castaneum.* These DNA *cis*-regulatory regions enable testes-specific EGFP expression when introduced into the *T. castaneum* germline via *piggyBac*-mediated transformation. Stage, uni-sex and tissue specific gene expression is vital for the development of novel pest insect control approaches and also for experiments plan to enhance or improve our knowledge of insect molecular biology.

## Materials and methods

### RT-PCR analysis

Beetle pupae were separated based on sex prior to eclosion. Ovaries, testes and carcasses from male and female adult beetles were collected at 7–8 days post eclosion (30 beetles in each replicate processed in a single day per sample). Samples were snap frozen in liquid nitrogen and then transferred to − 80 °C prior to RNA extraction. RNA-extraction and cDNA preparation were performed simultaneously with all samples. Total RNA from these tissues was extracted with Trizol (Invitrogen, Carlsbad, CA, USA) following the manufacturer’s instructions. Single-strand cDNA was synthesized following the manufacturer’s instructions (Fermentas). Primers for RT-PCR were designed by using the software Primer-3 (http://frodo.wi.mit.edu/). Primers were designed by the rules of highest maximum efficiency, and sensitivity rules were followed to avoid formation of self and hetero-dimers, hairpins, self-complementarity and specific to two exons spanning an intron boundary. The primer sequences used in this study are given in Table S1. In brief, single-stranded cDNA was synthesized as follows: 500 ng of total RNA in 11 µl of sterile deionized water previously treated with DNase using the DNA-free kit (Ambion www.ambion.com) following the manufacturer’s instructions. The reverse-transcriptase reaction to generate the cDNA for use in RT-PCR was carried out using the First Strand cDNA Synthesis kit (Fermentas) as follows: 1 μl of oligo d (T) primer was added to the 11 µl of total RNA. The mixture was heated at 65 °C for 5 min, and then placed on ice, and the following were added: 4 μl of 5× first-strand buffer, 2 μl of dNTPs, 1 µl of RNase inhibitor, and 1 µl of reverse transcriptase. cDNA synthesis was performed at 42 °C for 30 min and 50 °C for 60 min. Reactions were stopped by heating samples at 95 °C for 2 min.

RT-PCR amplification conditions were 10 min at 95 °C to activate the polymerase, followed by 30 cycles at 95 °C for 30 s, 58 °C for 30 s and 72 °C for 30 s. Ribosomal protein RpSL32 was used as reference gene, RpSL32 gene specific primer sequences used in this study are given in Table S1. RT-PCR was performed by using Q5 High fidelity DNA Polymerase (NEB).

### Plasmid construction

Gene cassettes containing β_2_t-tTA-P2A-EGFP, Rad50-tTA-P2A-EGFP and Eno-tTA-P2A-EGFP, were synthesized (Epoch life sciences) and cloned into pBluescript-II (SK) (+). Gibson assembly^35^ (NEB), was used to clone each expression cassette into the donor plasmid pBac-3XP3-DsREDafm^36^. tTA was used in constructs to assess its expression effect in *T. castaneum* as part of future research. An overlap of 20 nt was used in primer sequences for the assembly of two fragments (Supplementary Table S1). PCR was performed with Q5 High-Fidelity DNA Polymerase. A total of 0.03–0.2 pmols of each DNA fragment was used in the assembly with 0.01 pmols of vector. The completed plasmids were verified by sequencing.

### Development of transgenic lines

Prior to embryo collection, beetles were kept overnight on whole grain flour (29 °C) and switched to instant flour during the next day. Embryos were collected within two hours of oviposition and washed with luke-warm tap water at room temperature to remove any attached flour. Embryos were injected through the chorion with a mixture of phspBac helper^37^ ~ 300 ng/µl and donor ~ 500 ng/µl plasmid at the posterior end, injections completed within three hours of embryos collection. Injected embryos on each slide were transferred into a petri dish (without lid) and placed on a stand in a sealed plastic container with 100 ml of 2% salt solution (table salt in tap water; 99% relative high humidity) and incubated at 29 °C. At day 3 (injection day is day 0), later in the evening the petri dish was transferred into another plastic container with saturated salt solution (70% relative low humidity) and incubated at 29 °C until hatching. Larvae successfully hatched were counted, collected with a fine brush and transferred into a container with flour. G_0_ beetles surviving to adulthood were outcrossed to the white-eyed mutant strain, and G_1_ progeny assayed for DsRED expression using a Leica MZ165FC stereo fluorescence microscope. G_1_ DsRED + adult beetles were crossed with white-eyed beetles to establish each transgenic line.

### Microscopy

Confocal microscopy was performed at the TAMU Microscopy and Imaging Center using a Leica SP8 laser scanning confocal microscope (Leica Microsystems, Wetzlar, Germany) equipped with white laser, AOBS beam splitter and HyD detectors. HC Plan Apo 10×/0.4 dry objective and HC PL APO 20×/0.75 IMM CORR CS2 multi-immersion objective, used in a water immersion mode, were employed for imaging, with pinhole set to 1 Airy unit, excitation 488 nm, fluorescence emission 493–533 nm. Transmitted light images were collected at the same time.

### Genomic insertion loci of transgenes

Inverse PCR (iPCR)^38^ was performed for the isolation of inserted *piggyBac* elements in *T. castaneum*. For each transgenic strain, ten beetles were collected and placed into 1.5 ml Eppendorf tubes and frozen in liquid nitrogen. Total genomic DNA was extracted from transgenic lines using the Macherey–Nagel Nucleospin Tissue Kit and quantified with the Spectramax i3x. Genomic DNA of 1–3 µg from transgenic lines was digested with restriction enzymes Sau3AI, HaeII, HinP1I, HhaI, RsaI or HpyCH4III overnight at 37 °C; digested fragments were purified with the Nucleospin Tissue Kit and eluted in 35 µl of elution buffer. In ligation reaction 1 µg of purified digested DNA was self-ligated using T4 DNA Ligase (NEB) overnight at 16 °C, followed by a second purification step and collection in 30 µl of Elution buffer. First round PCR was performed using 2.5 µl of purified, circularized DNA, primers listed in Table S1, and Q5 polymerase (NEB). Cycle conditions were: 98 °C for 1 min, 54 °C for 45 s, and 72 °C for 1 min for 30 cycles. If no product was observed, a second, nested PCR was performed using 2.5 µl of 1st round PCR material (annealing temperature was shifted to 59 °C).

PCR products from different samples were purified from an agarose gel using the Macherey–Nagel Nucleospin Gel and PCR Clean up Kit; purified PCR products were quantified by Nanodrop and sequenced for insert and flanking genomic sequences. Insert sequences were aligned to the 5′ or 3′ *piggyBac* terminal sequences, with additional sequences as genomic flanking sequences. Genomic insertion sites were identified by comparison with the *T. castaneum* genome (Tcas5.2) using the blastn function as implemented by the i5K workspace (https://i5k.nal.usda.gov/). Putative insertions at specific sites in the *T. castaneum* chromosome were further confirmed by PCR amplification of genomic DNA using high fidelity Phusion polymerase (NEB) along with one primer landing within the 3’ UTR of the respective transgenic construct and with the other located in the genomic DNA of flour beetle (Supplementary Table S1).

### *T. castaneum* strains and rearing

The white-eyed *T. castaneum* strain used in this study arose from an unknown mutation present in the wild-type beetle population which we used previously for detailed transcriptomic analysis^39^. This sub-strain was selected for transformation experiments, as it lacks black eye pigments that would interfere with our ability to detect eyespecific red fluorescence. White-eyed and wild-type black-eyed beetles were reared separately on flour medium (95% flour, 5% yeast by weight), and caged in glass jars with tight-fitting fine mesh closures. Beetles were housed in a growth chamber at 29 °C with 60–80% relative humidity and 12/12-h light/dark cycling. Populations of beetles were moved to fresh flour medium once per month with initial population densities of approximately 1–2 beetles/1 g flour medium.

## Results

### Identification and characterization of β_2_Tubulin, Rad50-ATPase and enolase orthologues in ***T. castaneum***

We previously identified at least eighteen genes whose transcripts were substantially enriched in male testes compared to the rest of the body, female ovaries/body and early embryos^39^. We reasoned that these genes would be good candidates to donate *cis*-regulatory sequences that might be capable of driving transgene expression specifically in the testes. In order to select candidate genes, we calculated the distance from each testis-enriched gene to the next upstream and downstream gene in the *T. castaneum* genome (Supplementary Table S2). We reasoned that focusing on candidate genes with close neighbors would help ensure that the genomic fragments selected would contain the necessary *cis*-regulatory elements needed for testes-specific expression. We also considered intron length, focusing on genes with only short introns. Based on these criteria, we selected three testes-enriched genes for evaluation: *TC009035* (*β*_*2*_*-tubulin*), *TC006703* (*rad50)*, and *TC011729* (*enolase*).

Tubulin is the major constituent of microtubules, and testes-specific β-tubulin genes have been described in *Drosophila*, *Bombyx,* medfly and mosquitoes^11–13,23^. We identified four different β-tubulin orthologues *TC009589*, *TC034766*, *TC010829*, and *TC009035* in the *T. castaneum* reference genome (Supplementary Fig. S1, Supplementary Table S3). However, only *TC009035* (*β*_*2*_*-tubulin*) was found to have high expression in *T. castaneum* testes^39^, and we refer to this gene as *Tc-β*_*2*_*t* for simplicity. *Tc-β*_*2*_*t* is 95% identical at the amino acid level to the *D. melanogaster* orthologues β-Tub85D (*CG9359; FBgn0003889*) and *CG9222* (*FBgn0031784*), and groups with the β-Tub85D gene highly expressed in the *D. melanogaster* testis (Supplementary Fig. S1). Rad50 forms a dimer with Mre11 nuclease and is required for dsDNA break repair, telomere maintenance, and ataxia telangiectasia mutated kinase checkpoint signaling^40^. *TC015093,* referred to here as *Tc-rad50*, is the only *rad50* gene in the *T. castaneum* genome, and encodes a 1:1 orthologue of the vertebrate Rad50 protein (Supplementary Fig. S2, Supplementary Table S4). Enolase metallo-enzyme is responsible for the conversion of 2-phosphoglycerate into phosphoenolpyruvate, the second to last step in glycolysis process^41^. Unlike *Drosophila,* which encodes a single enolase gene, three enolase orthologues are present in *T. castaneum* (Supplementary Fig. S3, Supplementary Table S5), though only *TC011729*, which we refer to as *Tc-eno,* was strongly expressed in the testes^39^.

**Figure 1:**
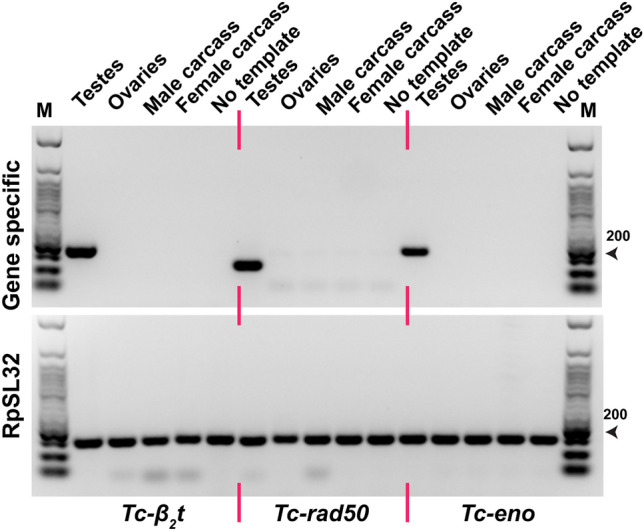
*Tc-β*_*2*_*t*, *Tc-rad50* and *Tc-eno* are expressed specifically in the testes of *T. castaneum*. RT-PCR-based transcript analysis of cDNA prepared from white-eye mutant *T. castaneum* adults. *T. castaneum* RpSL32 housekeeping gene was used to confirm the quality of each cDNA. Lane (M) indicates 50 bp DNA Ladder Catalog No. (N3236S) New England Biolabs.

To confirm the expression pattern of *Tc-β*_*2*_*t*, *Tc-rad50* and *Tc-eno* as testis-enriched*,* we performed reverse transcriptase PCR (RT-PCR) on total mRNA extracted from dissected adult tissues of *T. castaneum* (Fig. 1). PCR based analysis showed that *Tc-β*_*2*_*t*, *Tc-rad50* and *Tc-eno* transcripts could be found only in male testes and were not detectable in any other tested tissues such as female ovaries and both male and female carcasses (Fig. 1). Based on the tissue-restricted expression and amenable gene structure, potential *cis*-regulatory regions from the *Tc-β*_*2*_*t*, *Tc-rad50* and *Tc-eno* loci were selected for generating transposon-based transformation vectors.

### PiggyBac-based transformation of candidate regulatory sequences into the *T. castaneum* genome

**Figure 2:**
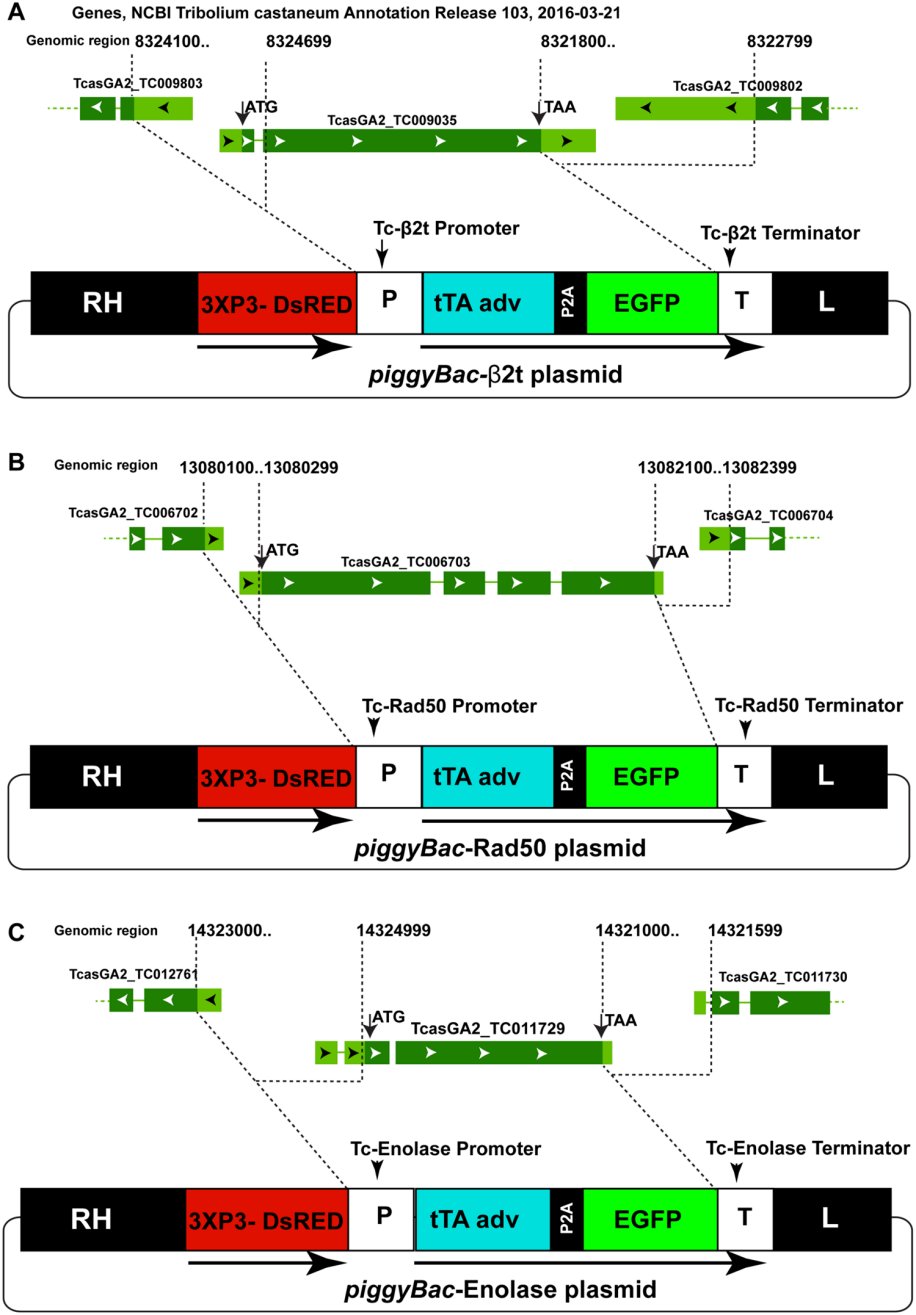
Schematic diagram of *Tc-β*_*2*_*t*, *Tc-rad50* and *Tc-eno* loci and corresponding transformation constructs. Schematic diagrams of the *Tc-β*_*2*_*t* genomic locus and *piggyBac*-β_2_t donor plasmid (**A**), the *Tc-rad50* locus and *piggyBac*-Rad50 donor plasmid (**B**), and the *Tc-eno* locus and *piggyBac*-Eno donor plasmid (**C**). All three constructs contained the 3XP3-DsRED cassette as a visual marker and *piggyBac* inverted repeats (RH and L) for transposase mediated integration in *T. castaneum* genome. tTA adv and EGFP were separated by the P2A site in all tested plasmids. P, putative promoter region; T, putative terminator region. Dotted lines indicate the upstream and downstream genomic regions used as promoter and terminator respectively to drive the tTA-P2A-EGFP expression in vector construction.

To determine if genomic sequences derived from *Tc-β*_*2*_*t, Tc-rad50* and *Tc-eno* could drive the expression of an EGFP reporter gene specifically in the testis we constructed three independent transformation plasmids based on the *piggyBac* transposon (Fig. 2). In all cases, genomic fragments corresponding to the entire genomic region upstream and downstream of the respective ORF were cloned upstream/downstream of the selected reporter gene (tTA-P2A-EGFP, Supplementary Table S6), bounded only by the sequence coding for the ORF of each neighboring gene (Fig. 2).

In addition, all constructs contained the 3xP3-DsRED cassette to serve as a visual marker for transformation and *piggyBac* inverted repeats for transposase mediated integration into the *T. castaneum* genome. As eye-specific DsRED expression was anticipated to be difficult to detect in wild-type beetles, we performed germline transformation in a white-eyed *T. castaneum* strain (Supplementary Fig. S4). Embryos from the white-eyed *T. castaneum* strain were injected using an hsp70-driven transposase helper plasmid^42^ in conjunction with each of the three *piggyBac* donor plasmids. In each case, transgenic founder events were recovered (Table 1).

**Table 1:** Summary of *piggyBac* based transformation experiments.

Exp	Donor plasmid	No of embryos injected	Hatched larvae	Number of adult G_0_	Female/male G_0_*	DsRED^+^ (#) G1
1	*piggyBac*-β_2_t	1640	152	80	42/38	15
2	*piggyBac*-Rad50	1750	151	101	58/43	13
3	*piggyBac*-Eno	1260	113	52	25/37	7

*Crossed the G_0_ putative transgenic female with w^−/−^ male in single pair cross or in pool of two to four beetles and vice versa. G1 larvae were screened for the DsRED fluorescence in eye.

### Spatial and temporal expression of EGFP in Tc-β_2_t-EGFP, Tc-Rad50-EGFP and Tc-Eno-EGFP lines

**Figure 3:**
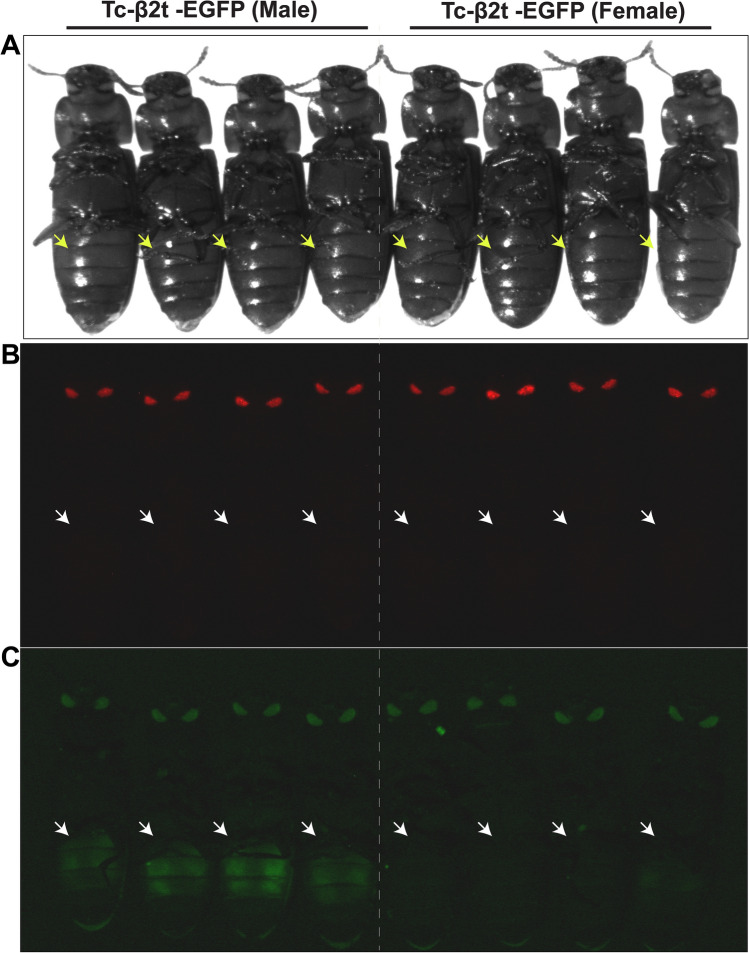
Fluorescent microphotographs showing sex-specific β2t drive EGFP expression in *T. castaneum*. (**A**) Transgenic adult male and female beetles viewed under bright field (**B**) DsRED filter and (**C**) GFP filter. Arrows indicate the abdomen where EGFP expression respectively was expected for transgenic male beetles in Tc-β_2_t-EGFP lines in gfp field.

**Figure 4:**
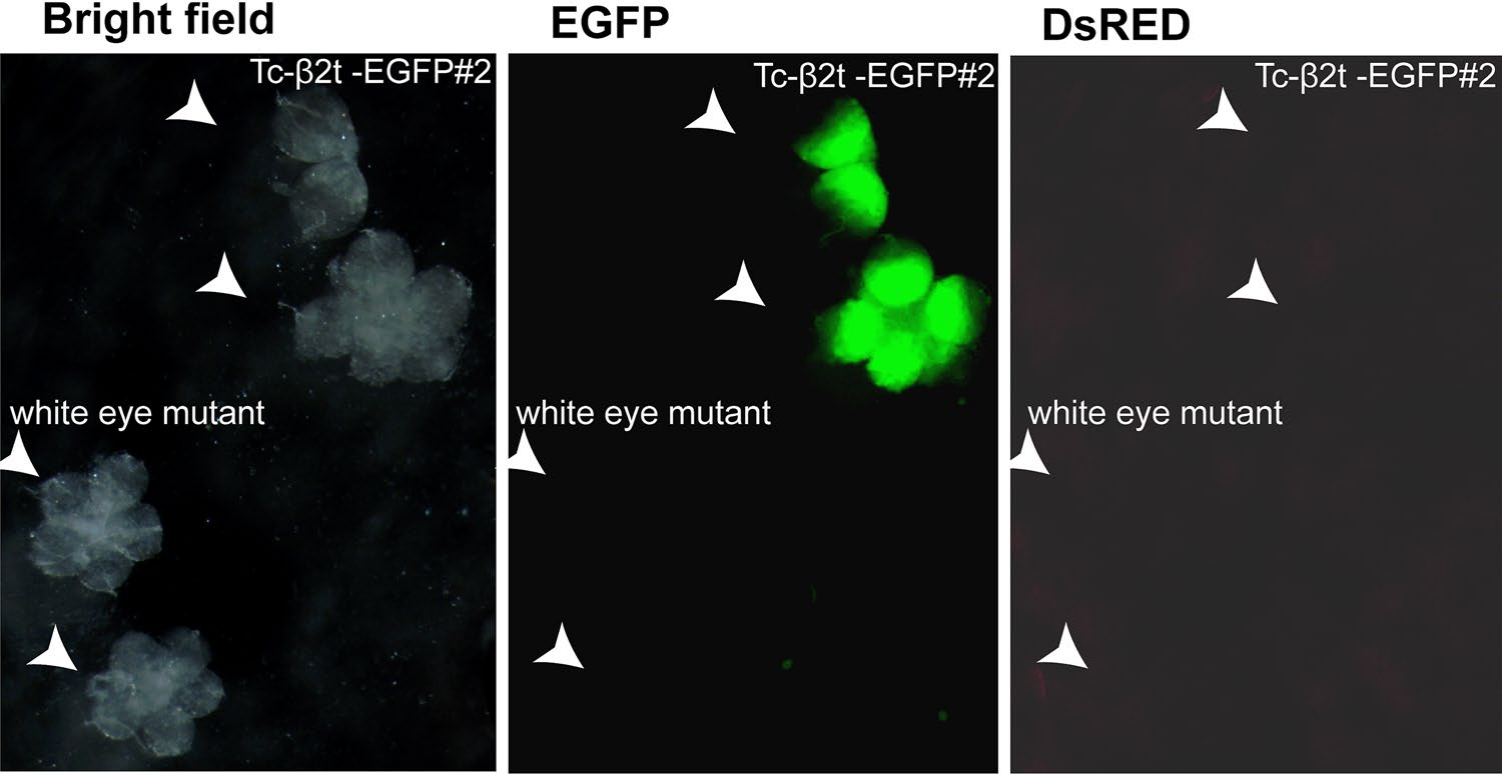
*Tc-β*_*2*_*t cis*-regulatory regions drive EGFP expression in testes. (**A**) Testes were dissected from Tc-β_2_t-EGFP#2 (top right) and white eye mutant (lower left) beetles and viewed under bright field, EGFP or DsRED filters.

Three independent transgenic *T. castaneum* lines, which we refer to as Tc-β_2_t-EGFP#1, Tc-β_2_t-EGFP#2 and Tc-β_2_t-EGFP#3 were produced following injection with *piggyBac*-β_2_t. Male and female beetles were separated based on black spots on the first pair of legs of male adults which are absent in females^43^. While some autofluorescence was visible in the beetle eyes in the green channel (Supplementary Fig. S5A), EGFP was visible only in the abdomen of male adults (Fig. 3). DsRED fluorescence was detected in the insect compound eyes, regardless of the beetle sex, while EGFP fluorescence was detectable exclusively in the male gonad (Fig. 4). This tissue specific expression pattern of EGFP in dissected testes from male adults from the Tc-β_2_t-EGFP line was similar to that reported for β_2_-tubulin in other insects^12,23,44^. As was expected, we did not detect EGFP in the ovaries of transgenic beetles in any Tc-β_2_t-EGFP lines (Supplementary Fig. S6). While reliable identification of EGFP expression in adult male beetles was feasible, we were not able to observe EGFP in pupae or late stage larvae due to auto-fluorescence (Supplementary Fig. S5B) for any Tc-β_2_t-EGFP lines. This is different from reports in mosquitoes, where detection of the reporter protein through the body wall was obvious in all developmental stages^12,44^.

**Figure 5:**
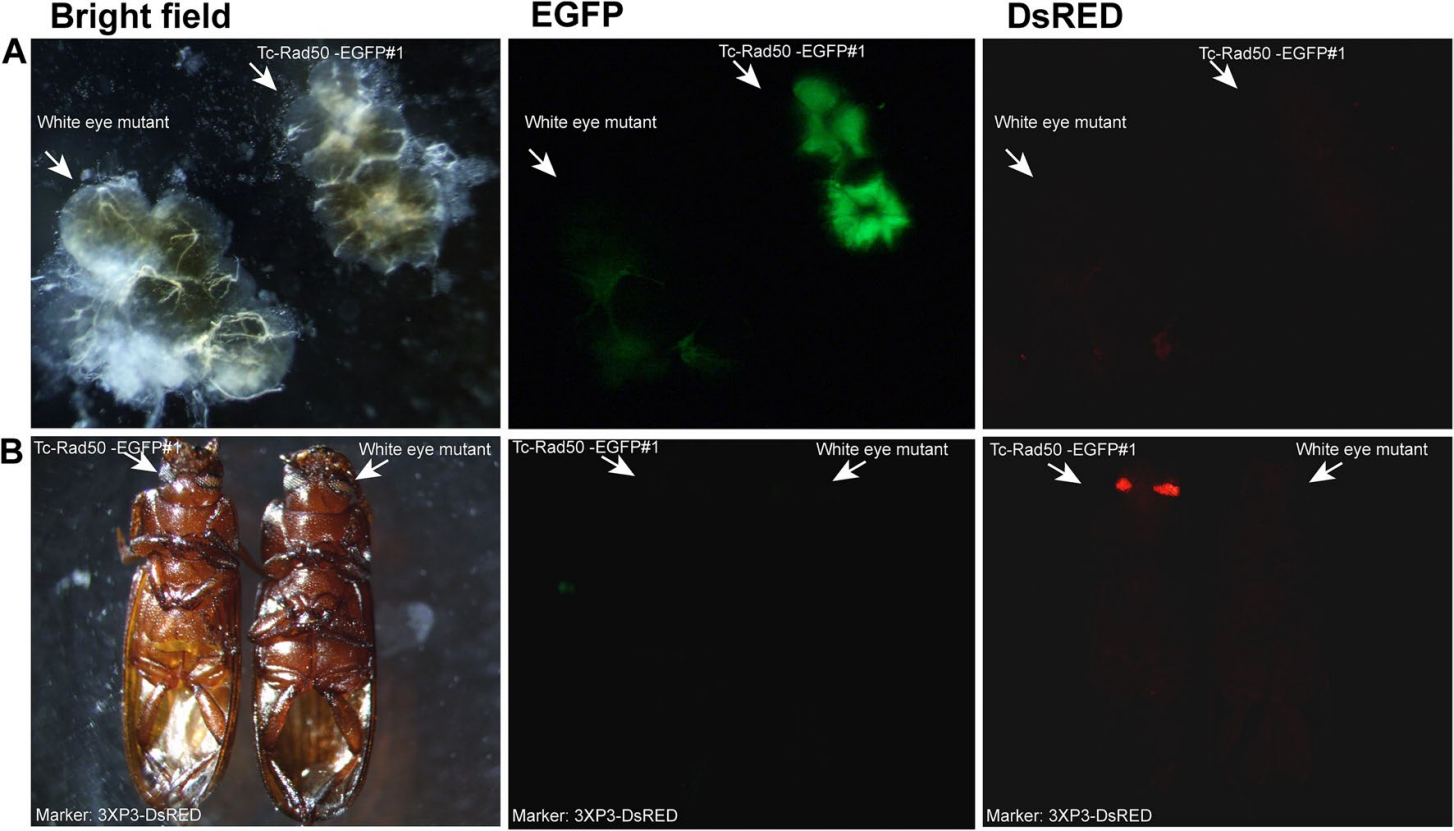
*Tc*-*rad50* regulatory regions drive EGFP expression in testes. (**A**) Testes from transgenic beetles (upper right) and non-transgenic beetles (lower left) viewed under bright field, EGFP or DsRED filters. (**B**) Adult transgenic (left) and non-transgenic (right) viewed under bright field, EGFP or DsRED filters. Arrows indicate the eyes and testes where DsRED and EGFP expression, respectively, were expected for transgenic Tc-Rad50-EGFP#1 line beetles.

Three independent transgenic *T. castaneum* lines were generated by using the *piggyBac*-Rad50 construct, Tc-Rad50-EGFP#1, Tc-Rad50-EGFP#2 and Tc-Rad50-EGFP#3. Once again, EGFP was detectable only in the male gonads in beetles from Tc-Rad50-EGFP lines (Fig. 5), and we did not detect EGFP fluorescence in ovaries from transgenic females (Supplementary Fig. S7). Unlike the Tc-β_2_t-EGFP lines, EGFP was not detectable in whole adults from Tc-Rad50-EGFP lines, while the marker gene DsRED was visible in the eyes at all developmental stages, irrespective of sex. Interestingly, the pattern of EGFP fluorescence was unique in Tc-Rad50-EGFP testes as compared to Tc-β_2_t-EGFP lines. The EGFP in these beetles appeared less intense as compared to the testes from Tc-β_2_t-EGFP beetles, and was not as widely distributed along the spherical shape of the testes (Fig. 5). For both Tc-β_2_t-EGFP and Tc-Rad50-EGFP constructs, the pattern and specificity of EGFP expression was similar for separate transgenic events, with EGFP fluorescence detectable only in the gonads of males (Supplementary Fig. S8).

**Figure 6:**
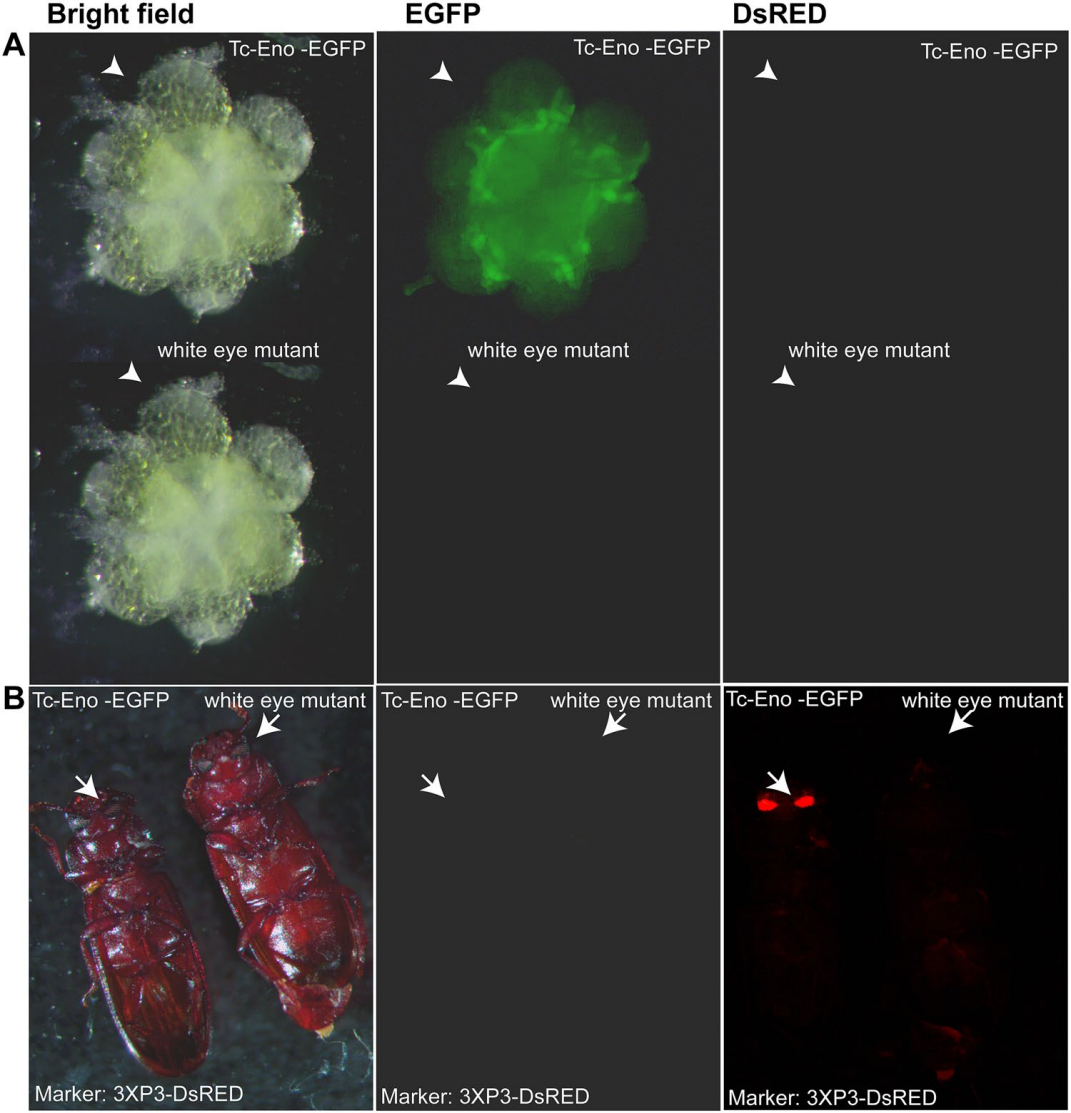
*Tc*-eno regulatory regions drive EGFP expression in testes. (**A**) Testes from transgenic beetles (upper right) and non-transgenic beetles (lower left) viewed under bright field, EGFP or DsRED filters. (**B**) Adult transgenic (left) and non-transgenic (right) viewed under bright field, EGFP or DsRED filters. Arrows indicate the eyes and testes where DsRED and EGFP expression respectively were expected for transgenic Tc-Eno-EGFP#1 line beetles.

Finally, a single transgenic *T. castaneum* line was generated using the *piggyBac*-Enolase construct; Tc-Eno-EGFP#1. In all tested beetles, the EGFP was detectable from dissected transgenic male adults, corresponding to the region where spermatogenesis^45^ is completed. As in adult beetles from the Tc-Rad50-EGFP lines, EGFP was detectable only in the male dissected gonad (Fig. 6). The intensity of EGFP from dissected testes appeared to be less than that observed for Tc-β_2_t-EGFP and Tc-Rad50-EGFP lines, and no fluorescence was detected in testes and ovaries collected from wild-type beetles. However, we interpret these data with caution as only a single line was developed.

**Figure 7:**
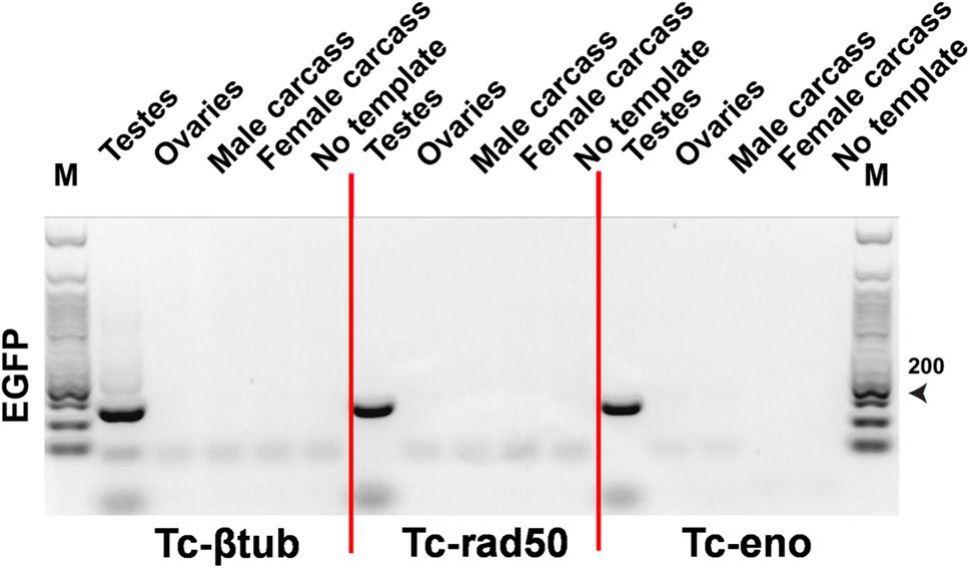
Transcription of the EGFP reporter in transgenic strains is testes-specific. cDNA from Tc-β_2_t-EGFP#2, Tc-Rad50-EGFP#1 and Tc-Eno-EGFP#1 transgenic lines was used to perform the RT-PCR. *T. castaneum* RpSL32 served as a control for cDNA quality (same samples as shown in Fig. 1). Lane#M is 50 bp DNA Ladder Catalog No. (N3236S) New England Biolabs.

In addition to measuring EGFP fluorescence, the expression of EGFP transcripts was examined by RT-PCR in transgenic beetles. In all cases, EGFP transcripts were detectable in the gonads of males, but not in ovaries, or male and female carcasses (Fig. 7), mimicking the expression pattern of the endogenous *Tc-β*_*2*_*t*, *Tc-rad50* and *Tc-eno* loci in *T. castaneum* male testes (Fig. 1).

**Figure 8:**
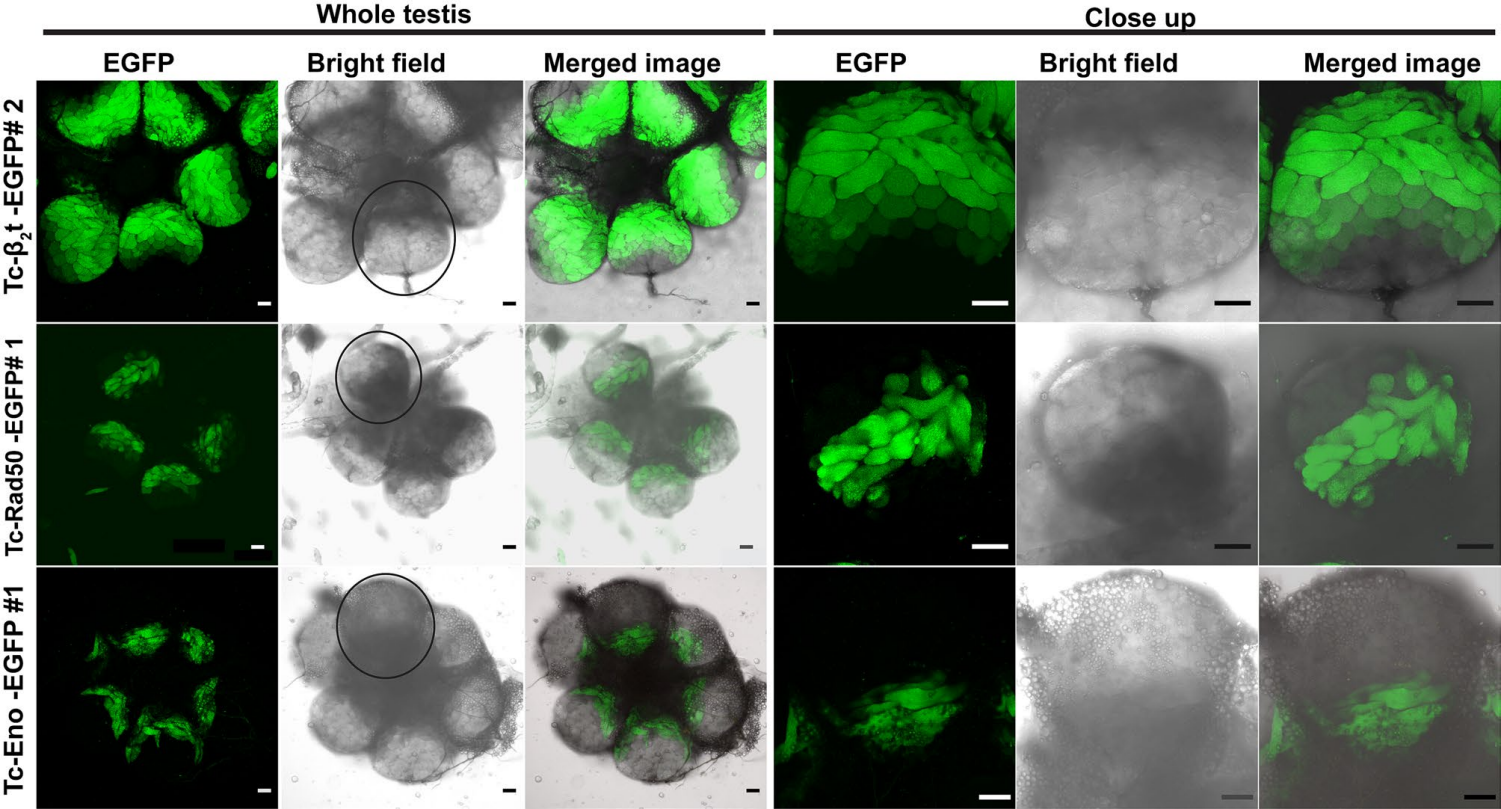
Confocal analysis of EGFP expression in transgenic male gonad. Expression of EGFP from transgenic males in whole testes and close up of single spherical testis dissected from Tc-β_2_t-EGFP#2, Tc-Rad50-EGFP#1, and Tc-Eno-EGFP#1 beetles. Black circle around the area in the whole testis corresponds to the close up image. Scale bar indicates 50 µm.

**Figure 9:**
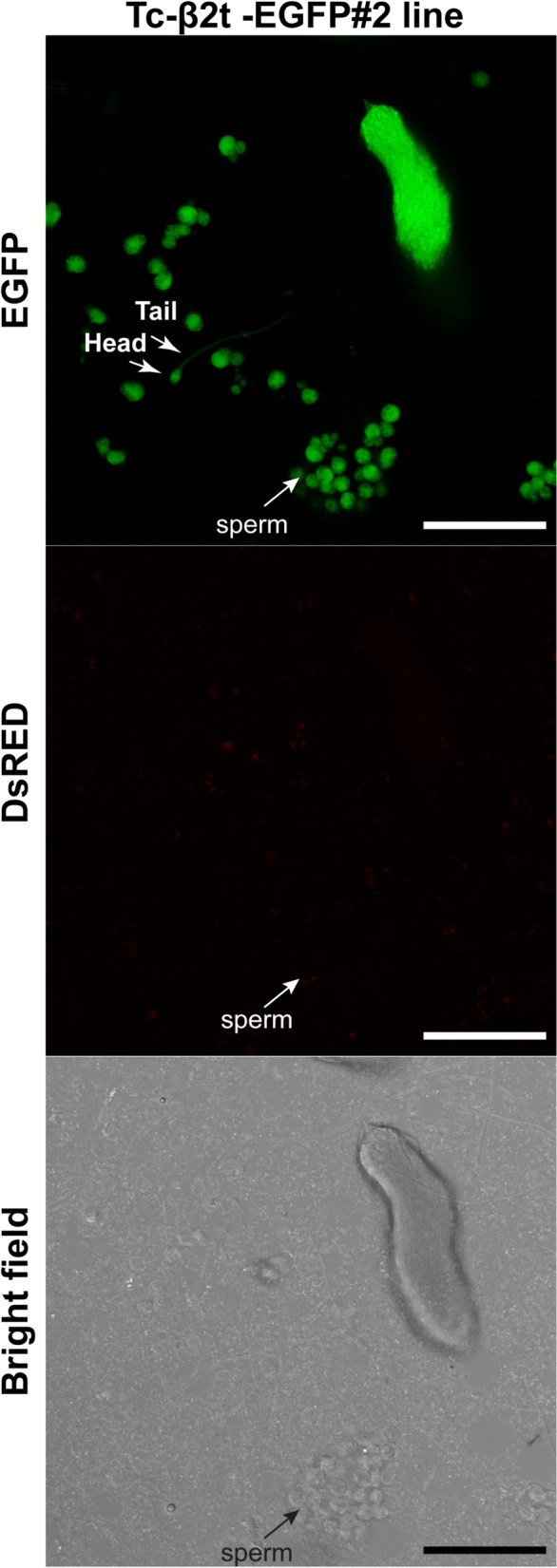
*Tc-β2t* drives EGFP expression in mature sperm. Sperm from testis of Tc-β_2_t-EGFP#2 viewed under EGFP, DsRED and Bright field filters. Green fluorescence can be visualized among single sperm as indicated by arrows. Scale bar indicates 50 µm.

To further analyze the expression pattern of the fluorescent reporter within the male beetle gonad, confocal microscopic analyses were performed on dissected testes from transgenic beetles from Tc-β_2_t-EGFP, Tc-Rad50-EGFP and Tc-Eno-EGFP lines. Dissected testes from Tc-β_2_t-EGFP individuals confirmed a very strong and widespread distribution of EGFP fluorescence along the longitudinal axis, ranging from the gonial (primitive germ cells) amplification stages, developing spermatocytes, spermatids, and spermatozoa^45^ (Fig. 8), up to individual mature sperm cells (Fig. 9). However, EGFP was not detectable in the apical tip of the testes (Fig. 8), indicating that the cloned *cis*-regulatory regions from the *Tc-β*_*2*_*t* locus in *piggyBac*-β_2_t plasmid did not direct EGFP expression in hub cells, male germline stem cells and somatic stem cells.

**Figure 10:**
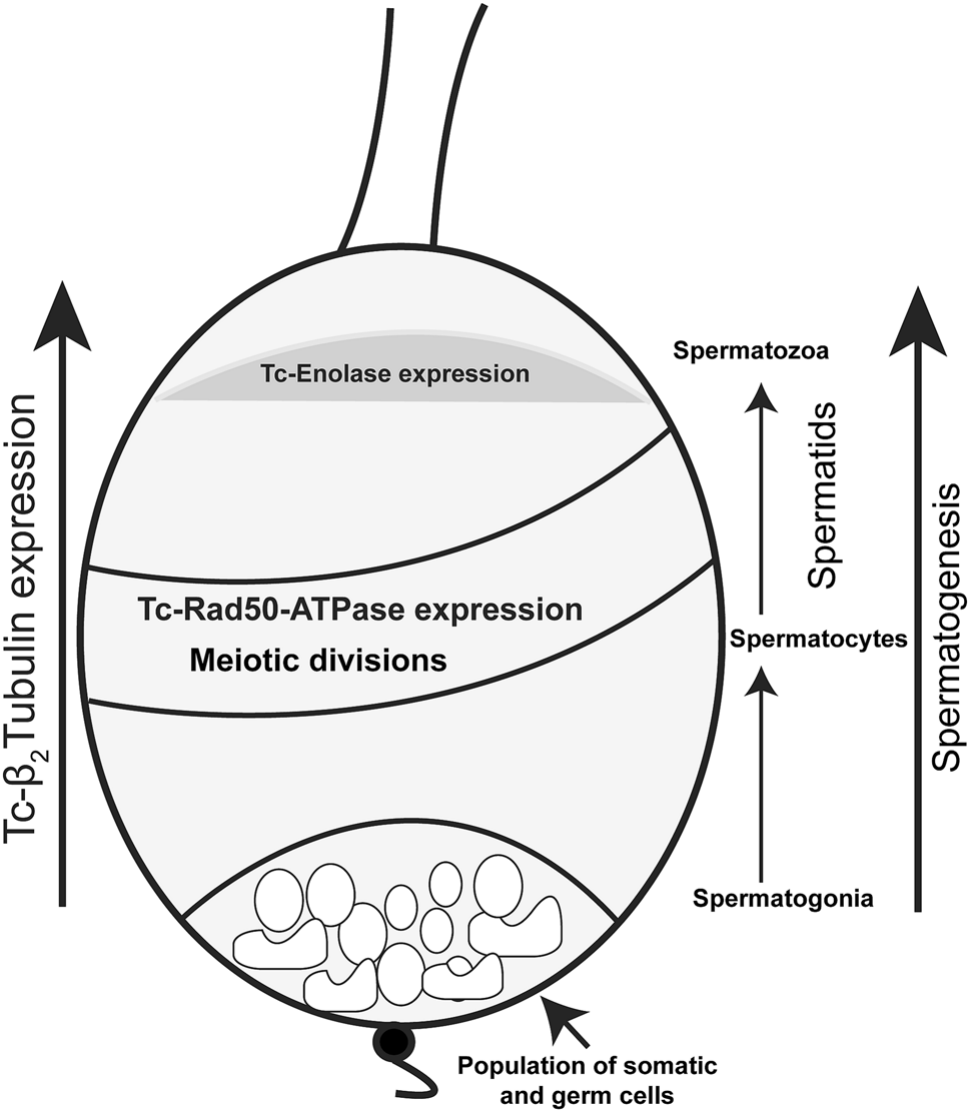
Diagram of the organization of a *T. castaneum* testis. The stem cell niche which is maintained by Stromal hub cells adheres to the apical tip of the testis. Hub cells are surrounded by germ line stem cells and somatic stem cells. Germ cells differentiate into spermatogonia and begin the development of spermatogenesis process to become mature sperm. Relative expression patterns of *Tc-β2t*, *Tc-eno* and *Tc-rad50* are noted.

In Tc-Rad50-EGFP beetles, EGFP fluorescence was detected only in elongated spermatid cells (Fig. 8). EGFP was not detectable in all stages of spermatogenesis including the germ stem cells in the apical tip, thus indicating that the *cis*-regulatory region from the *Tc-rad50* locus was active specifically in spermatid cells during spermatogenesis (Figs. 8, 10). Below the elongated spermatid cells, round spermatogonia, spermatocytes and germ stem cells, presumably the somatic stem cells surrounding the germ stem cells did not reveal any EGFP fluorescence in dissected testes from Tc-Rad50-EGFP male testes. No EGFP was detected in mature sperm from the beetles in Tc-Rad50-EGFP beetles again supporting the idea that the *cis*-regulatory regions derived from the *Tc-rad50* gene regulated EGFP expression in the *T. castaneum* testes differently than *cis*-regulatory regions from the *Tc-β*_*2*_*t* locus (Figs. 8, 9, 10). Similarly, testis in Tc-Eno-EGFP beetles were also examined in confocal microscopic analysis where we observed that EGFP fluorescence was entirely localized to cells transformed from spermatids into spermatozoa by the process of spermiogenesis (Figs. 8, 10). As in Tc-Rad50-EGFP beetles, Tc-Eno-EGFP was not detectable in mature sperm. No EGFP fluorescence was detected in the testes and ovaries from the wild-type black eye or untransformed white-eyed beetles (Supplementary Fig. S9).

### Insertion site detection

**Figure 11:**
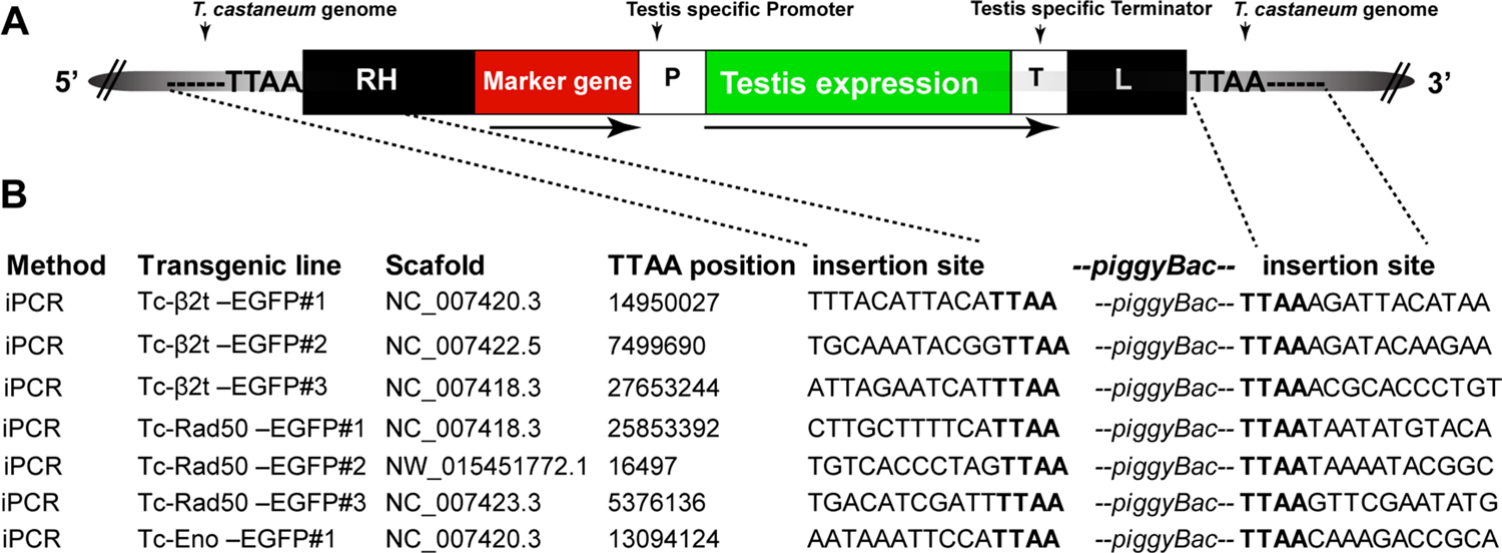
Inverse PCR strategies to isolate and sequence the *piggyBac* vector and insertion sites in different transgenic lines. (**A**) Schematic diagram (not to scale) of the *piggBac* vector insertion in the *T. castaneum* genome. The vector contained the marker cassette as a visual marker and *piggyBac* inverted repeats (RH and L) for transposase mediated integration in *T. castaneum* genome. *P* putative promoter region, *T* putative terminator region. Duplicated TTAA flanked the *piggyBac* elements in the host genome. (**B**) Below are shown the TTAA position in the *T. castaneum* genome where the transgenes were inserted and the flanked *piggyBac* transgenes insertion site sequences from the transgenic lines.

Insertion junctions for each line were subsequently determined using inverse PCR (iPCR)^38^ (Fig. 11). Sequences analysis confirmed that all insertions terminate correctly with expected *piggyBac* inverted repeats and that all are flanked by the normal *piggyBac* (TTAA) target sequence. Transgenes insertion were further confirmed by direct amplification of genomic DNA using Phusion polymerase (NEB) along with one primer located within the 3′ UTR of the respective transgenic construct, with the other located in the genomic DNA of host *T. castaneum*. We conclude that each element did indeed integrate into unique locations in the *T. castaneum* genome.

## Discussion

Here, we report on the development of transgenic strains expressing a fluorescent marker specifically in the male gonads of *T. castaneum*, a model for coleopterans and an important pest of stored grain. While several promoters have been characterized from or for *T. castaneum* to control transgene expression, these have been restricted to activity only in embryos/muscles such as twist^46^, caudal^47^, hunchback^47^, nubbin^32^, hairy^48^, and tailless^49^, hsp68^50^ or lack tissue-specificity as with the constitutive promoter Polyubiquitin^51,52^. In this report we show that *cis*-regulatory regions derived from three different *T. castaneum* genes were capable of controlling transgene expression specific to the testes.

The β_2_-tubulin promoter has been successfully used in other insects for transgenic male sexing. In *Drosophila melanogaster* and mosquitos, β_2_-tubulin transcripts are detectable in the male gonads from late larval developmental stages throughout later stages of sperm development^44,53^. In *Drosophila*, this gene is transcribed in late third larval instar before the onset of meiosis in the developing testis and remains active throughout adulthood^54^. Low level expression of β-Tub85D was also reported in other tissues in the fly such as in adult carcass and larval fat body^55^. In *A. aegypti* fourth instar larvae and pupae were easily scored as positive or negative for DsRED driven by the β_2_-tubulin promoter, these were confirmed as males upon adult emergence^12^. Similarly, in *An. stephensi,* EGFP driven by β_2_-tubulin promoter was used in automated sex sorting. Mosquitoes were separated during the larval stages and all larvae identified with green fluorescence phenotypes developed into males, while all larvae lacking EGFP were confirmed as female^44^. Like mosquitoes^12,44^, in the beetle we also found that EGFP expressed by the *Tc-β*_*2*_*t* regulatory regions could be detected through the body wall in male adults. The male gonad specific expression of EGFP under the tight control of the *Tc-β*_*2*_*t* cis regulatory region in *T. castaneum* provides an efficient, male-specific marker that can be used for sex sorting. In dissected testes, *Tc-β*_*2*_*t* controlled EGFP signals were not observed until spermatogonia reached the primary spermatocyte stage, with EGFP remaining present upon completion of spermatogenesis and in mature sperm.

Rad50 plays a key role in double stranded DNA break repair^40,56^, and to our knowledge our work represents the first use of a rad50 promoter to drive transgene expression in insects. Interestingly, low Rad50 expression was linked with spermatogenic failure in humans^57^, suggesting a potential conserved role in this process. In spermatogenesis, meiotic cell division is a vital step during which diploid spermatocytes generate haploid spermatids. This process is initiated by the formation of DNA double-strand breaks at specific sites^58^, which may need to be repaired using a complex containing of certain proteins such as MRE11, Rad50, ATM, NBS1 and Rad51^59^. Though meiotic cell division also takes place in eggs, Rad50 is not expressed in the ovary, suggesting other repair complexes may be dominant in the female germline. While much additional work is required to evaluate the role of DNA repair in beetle gonadal development, in our study we were unable to detect *Tc-rad50* driven EGFP in dissected ovaries. Like in *B. mori* where the Bmβ4-promoter was found to drive EGFP expression only in the microtubule of testes^13^, similarly the *Tc-rad50 cis*-regulatory region expressed EGFP in dissected testes but we did not observe EGFP signals in mature sperm.

The structure of *Drosophila* enolase has been characterized and the mature protein forms a homodimer with conserved residues at the dimer interface^60^. Fly enolase has an open conformation in its structure and has conserved residue elements for catalytic activity^60^. Enolase contains conserved key amino acid residues for metal binding (magnesium ion binding) and substrate binding (phosphopyruvate hydratase activity). The fly genome encodes one enolase and the *B. mori* genome has two enolase orthologues, in which one was shown to have high testis expression^61^. *T. castaneum* encodes three enolase genes, in which only the *Tc-eno* used in this study has high expression in testes^39^. The pattern of EGFP expression from the *Tc-eno* regulatory regions in beetle testes was distinct from the other two tested regulatory elements. While *Tc-eno* EGFP was not observed in mature sperm, the endogenous gene product may assist with providing energy to fuel sperm mobility.

Our investigation evaluating the *Tc-β*_*2*_*t*, *Tc-rad50* and *Tc-eno* loci *cis*-regulatory regions for male gonad expression and function in the *T. castaneum* model system and could be extended to other related insects. While the *β*_*2*_*t* promoter^12,13,15,62^ is well studied in other insects, here we report the rad50 and eno based reporter gene expression in insect testis for the first time. EGFP fluorescence was readily detected in *Tc-β*_*2*_*t* adult beetles and could potentially be used for non-lethal approaches in sex separation in the adult stage. In our experiments, EGFP expression alone could not be used as a marker to predict sex in beetles at the larval and pupal stages. This complicates the use of simple reporter constructs as presented here from being used for non-lethal approaches in sex separation in early developmental stages as seen for the β_2-_tubulin promoter in *Drosophila* and different mosquitos’ species^12,44,53^. It is possible that this could be overcome with the use of alternative reporters and/or using filters sets that minimize autofluorescence. β4-Tubulin in transgenic silkworm also drives EGFP expression in testis from late stage larvae to adult stage^13^, however, these authors did not report if the EGFP signal from transgenic animals could be detected through non-lethal approaches, as was reported in mosquitos^12,44^. The EGFP pattern driven by Bmβ4p was different from the *Tc-β*_*2*_*t*, where EGFP was detectable in the microtubules of testis from the dissected late larval to adult stages^13^. All of our transgenic strains were designed to also express the tetracycline transactivator (tTA), in addition to EGFP, through the use of the P2A viral sequence. While have not yet analyzed these strains for their ability to drive the expression of a gene under the control of tetO, this is a priority for future research, where tTA could activate the expression of a lethal gene^8^ or impair mating ability specifically in males. We note that male beetles from each strain remain fertile, so the level of tTA expression in the testes is considered below the threshold for potential strong toxicity. In conclusion, we have successfully established three male-specific reporter transgene systems in *T. castaneum*. The regulatory elements we characterized could be used for functional analysis of processes occurring in the testes in this and other related insects as well as for the development of transgenic strategies for adult male sexing in important agricultural pest species such as *T. castaneum*.

## Supplementary Information

Supplementary Information.

## Data Availability

All data generated or analyzed during this study are included in this published article (and its Supplementary Information files).
