# Author Correction: The β_2_Tubulin, Rad50-ATPase and enolase *cis*-regulatory regions mediate male germline expression in *Tribolium castaneum*

**DOI:** 10.1038/s41598-021-01236-z

**Published:** 2021-10-28

**Authors:** Sher Afzal Khan, Emma Jakes, Kevin M. Myles, Zach N. Adelman

**Affiliations:** 1 Department of Ecology and Genetics, Animal Ecology, Uppsala University, Norbyvägen 18 D, 752 36 Uppsala, Sweden; 2 Department of Entomology and Agrilife Research, Texas A & M University, College Station, TX 77843 USA

Correction to: *Scientific Reports* 10.1038/s41598-021-97443-9, published online 13 September 2021

The original version of this Article contained errors in the Figure legends of Figure 3 and Figure 4. The legends of these Figures were inadvertently switched.

The legend of Figure 3:

“*Tc-β*_*2*_*t cis*-regulatory regions drive EGFP expression in testes. (**A**) Testes were dissected from Tc-β_2_t-EGFP#2 (top right) and white eye mutant (lower left) beetles and viewed under bright field, EGFP or DsRED filters.”

now reads:

“Fluorescent microphotographs showing sex-specific β2t drive EGFP expression in *T. castaneum*. (**A**) Transgenic adult male and female beetles viewed under bright field (**B**) DsRED filter and (**C**) GFP filter. Arrows indicate the abdomen where EGFP expression respectively was expected for transgenic male beetles in Tc-β_2_t-EGFP lines in gfp field.”

The legend of Figure 4:

“Fluorescent microphotographs showing sex-specific β2t drive EGFP expression in *T. castaneum*. (**A**) Transgenic adult male and female beetles viewed under bright field (**B**) DsRED filter and (**C**) GFP filter. Arrows indicate the abdomen where EGFP expression respectively was expected for transgenic male beetles in Tc-β_2_t-EGFP lines in gfp field.”

now reads:

“*Tc-β*_*2*_*t cis*-regulatory regions drive EGFP expression in testes. (**A**) Testes were dissected from Tc-β_2_t-EGFP#2 (top right) and white eye mutant (lower left) beetles and viewed under bright field, EGFP or DsRED filters.”

The original Article has been corrected.

