# Supplementary material for: The β2Tubulin, Rad50-ATPase and enolase cis-regulatory regions mediate male germline expression in Tribolium castaneum
Source: Sci Rep. 2021 Sep 13;11:18131. doi: 10.1038/s41598-021-97443-9 (PMC8438054; doi:10.1038/s41598-021-97443-9)
Supplement: Supplementary file 1 — Supplementary Information. [file 41598_2021_97443_MOESM1_ESM.docx]

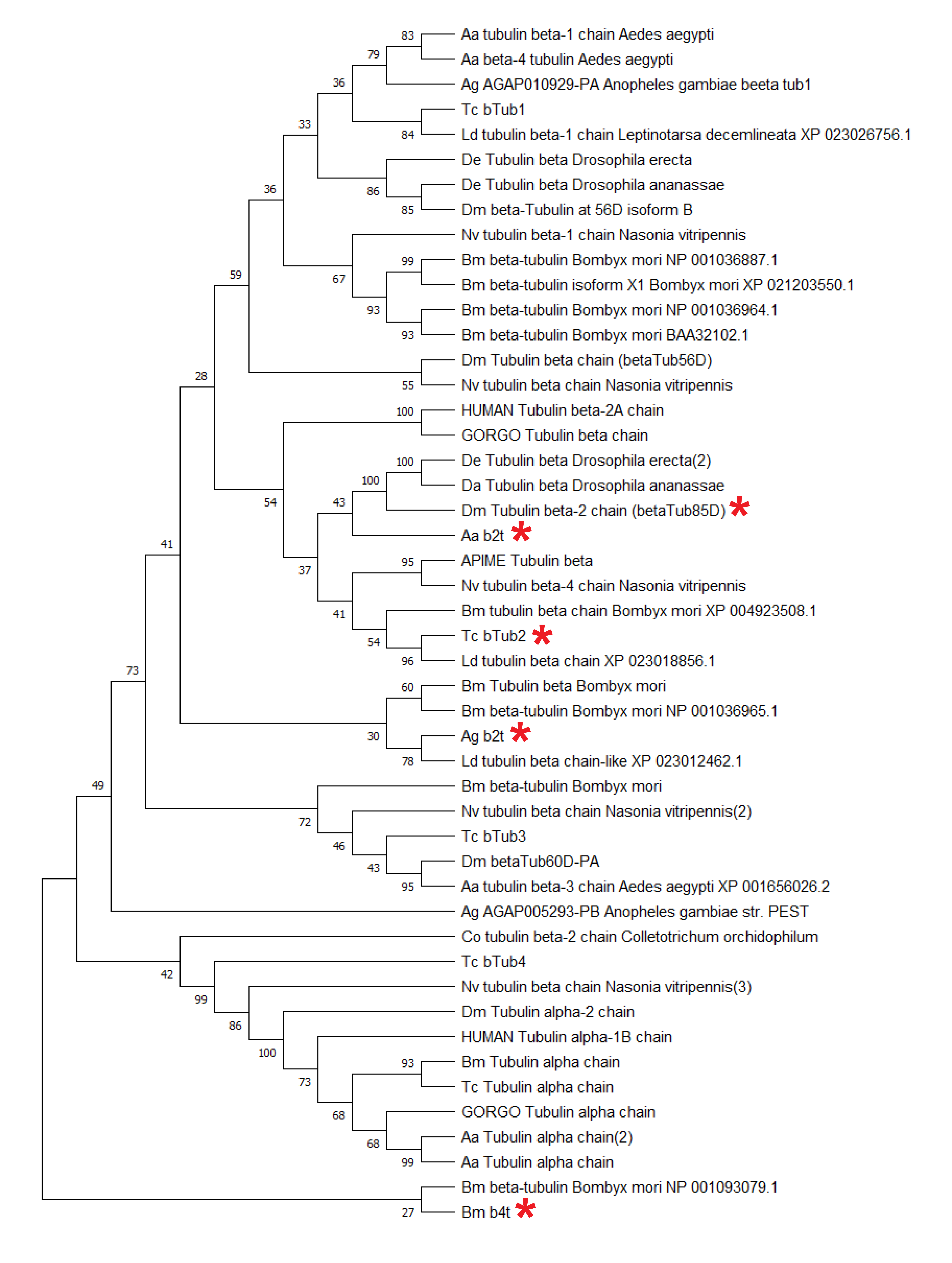


**Fig. S1.** **Phylogenetic tree inferred from Neighbor-joining analysis based on β-Tubulin, amino acids sequences for different organisms**. Reconstructed phylogenetic tree based on the amino acid sequences of β- Tubulin orthologues for different species as given in (Table S2) using the Neighbor-joining method with default parameters and 1000 bootstrapping implemented in MEGA10. Asterix (*) indicates the promoter from the β2 tubulin genes were used to express a transgene in testis from these insects, where the pattern of EGFP observed in testes from Bmβ_4_t –EGFP was different than for Tc-β_2_t, Dm-β_2_t, Aa-β_2_t and Ag-β_2_t.


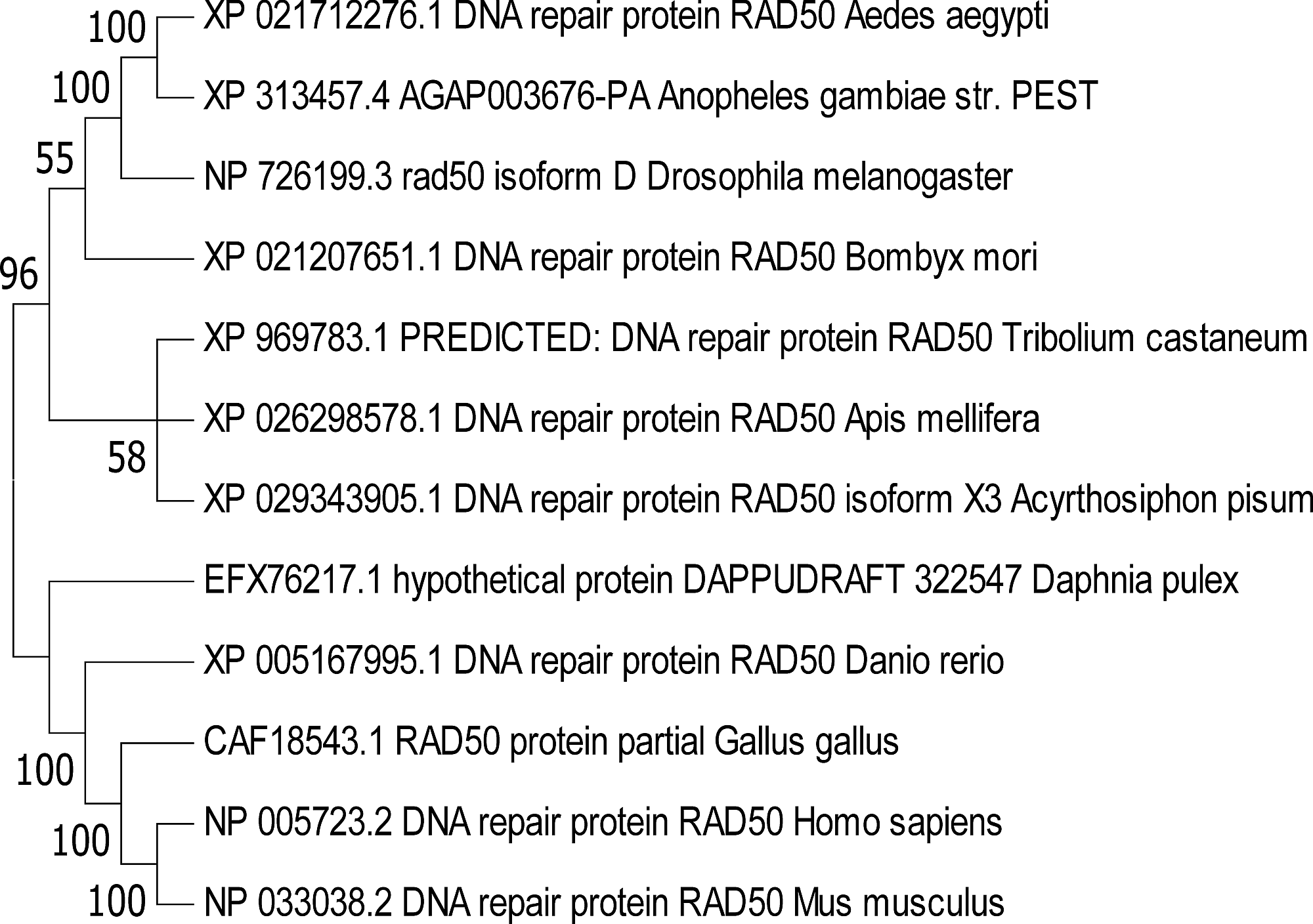


**Fig. S2.** **TcRad50 is a single copy ortholog of insect and vertebrate rad50 proteins.** Phylogenetic tree produced using the Neighbor-Joining method in MEGA following alignment of the respective protein sequences using MUSCLE. GenBank accession numbers are indicated for each sequence; % confidence is indicated for each branch when >50% following 1000 bootstrap replicates. Branches will less than 50% supports were collapsed.


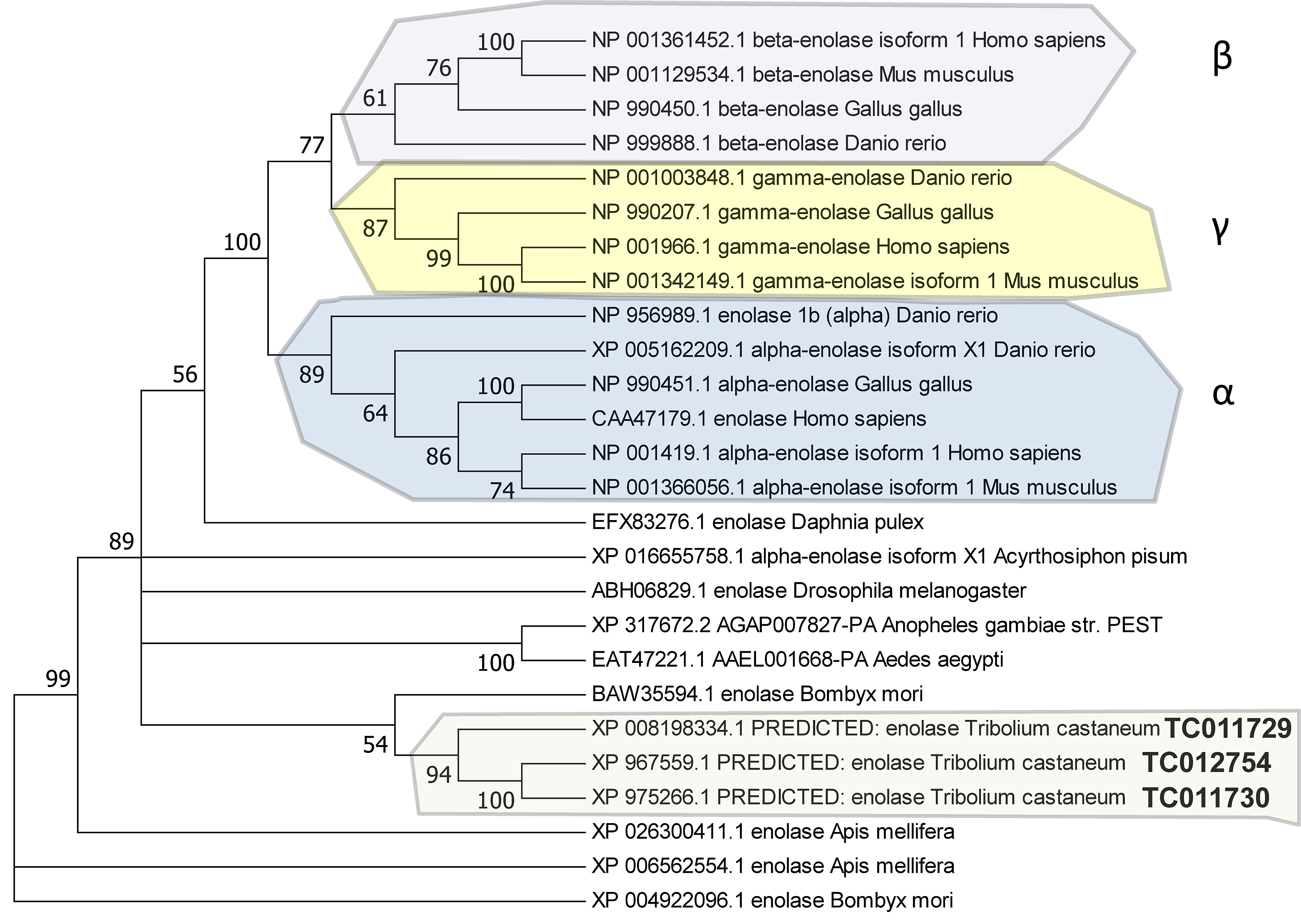


**Fig. S3.** **TC011729 (*Tc-eno*) and its paralogs compared to other insect and vertebrate enolase proteins.** Phylogenetic tree produced using the Neighbor-Joining method in MEGA following alignment of the respective protein sequences using MUSCLE. GenBank accession numbers are indicated for each sequence; % confidence is indicated for each branch when >50% following 1000 bootstrap replicates. Branches will less than 50% support were collapsed. Vertebrate α, β, and γ clades are indicated.


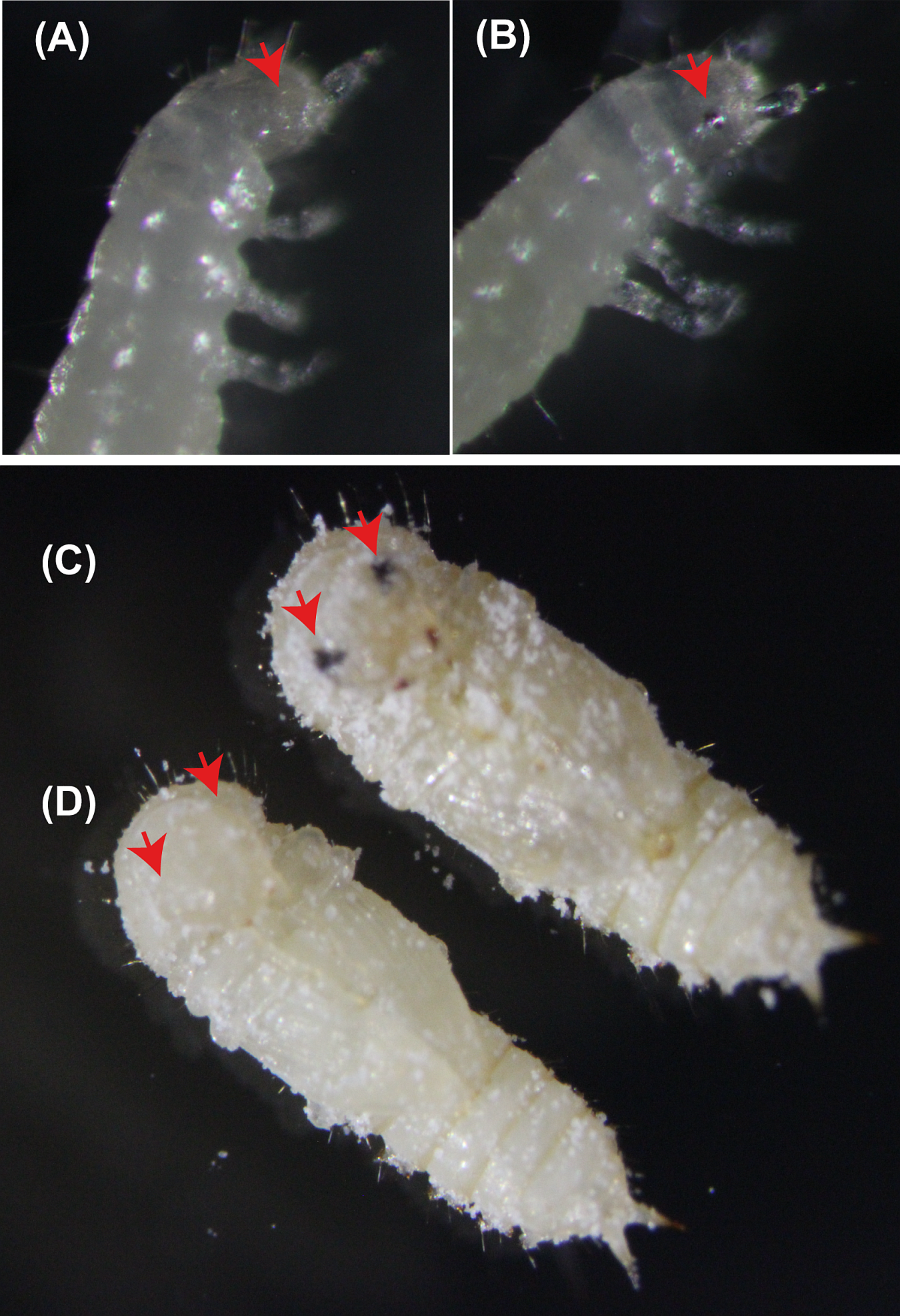


**Fig. S4.** **White eyed phenotypes in larvae and pupa of *T. castaneum*** (A) white eyed larva; (B) Wild type larva; (C) Wild type pupa and (D) homozygous white eyed pupa. Arrows indicate the white eyed and wild type phenotypes in larvae and pupae.


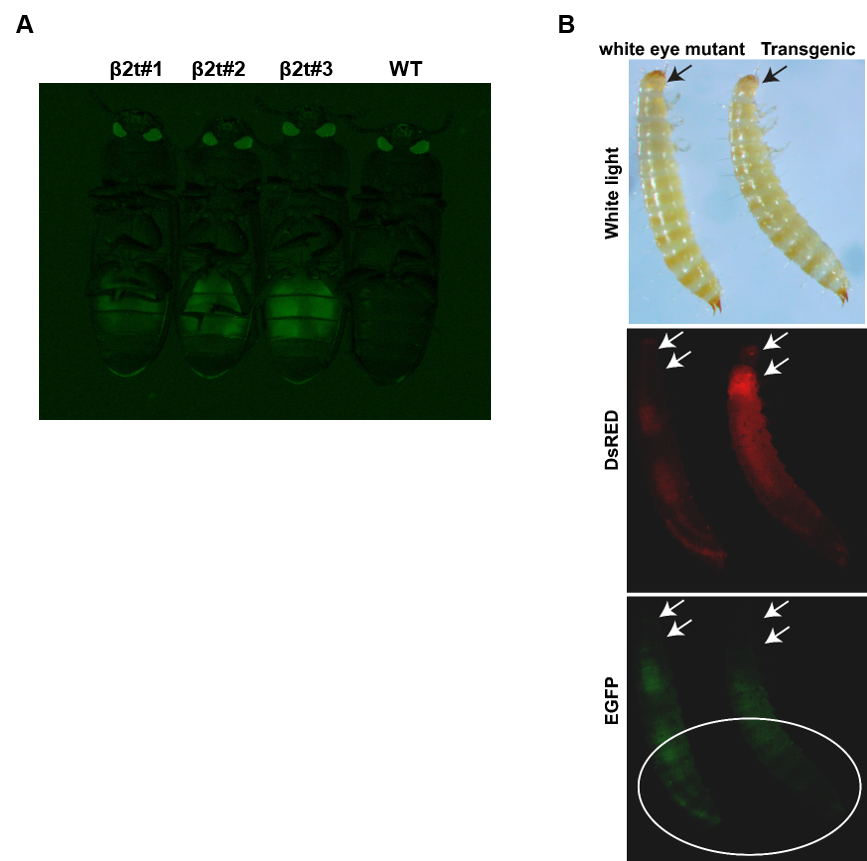


**Fig. S5. No reporter protein fluorescence detected in larvae. (A)** Adult male beetles from each transgenic B2t line along with the wild-type (WT) parental strain as viewed for EGFP fluorescence. **(B)** No EGFP expression was observed in the last instar larvae in Tc-β_2_t –EGFP#2 line (right) and white eye mutant (left) in bright field. Transgenic Tc-β_2_t –EGFP#2 larva showed DsRED fluorescence in the larva eyes in DsRED filter, while the white-eyed mutant larva had no florescence signal. Neither the transgenic nor the white-eyed mutant larvae have shown EGFP florescence in GFP filter. Arrows indicate to the larvae eye and nerve cord, white oval line indicates expected gonad region in larvae and these larvae developed as male pupae. Magnification 2X.

**
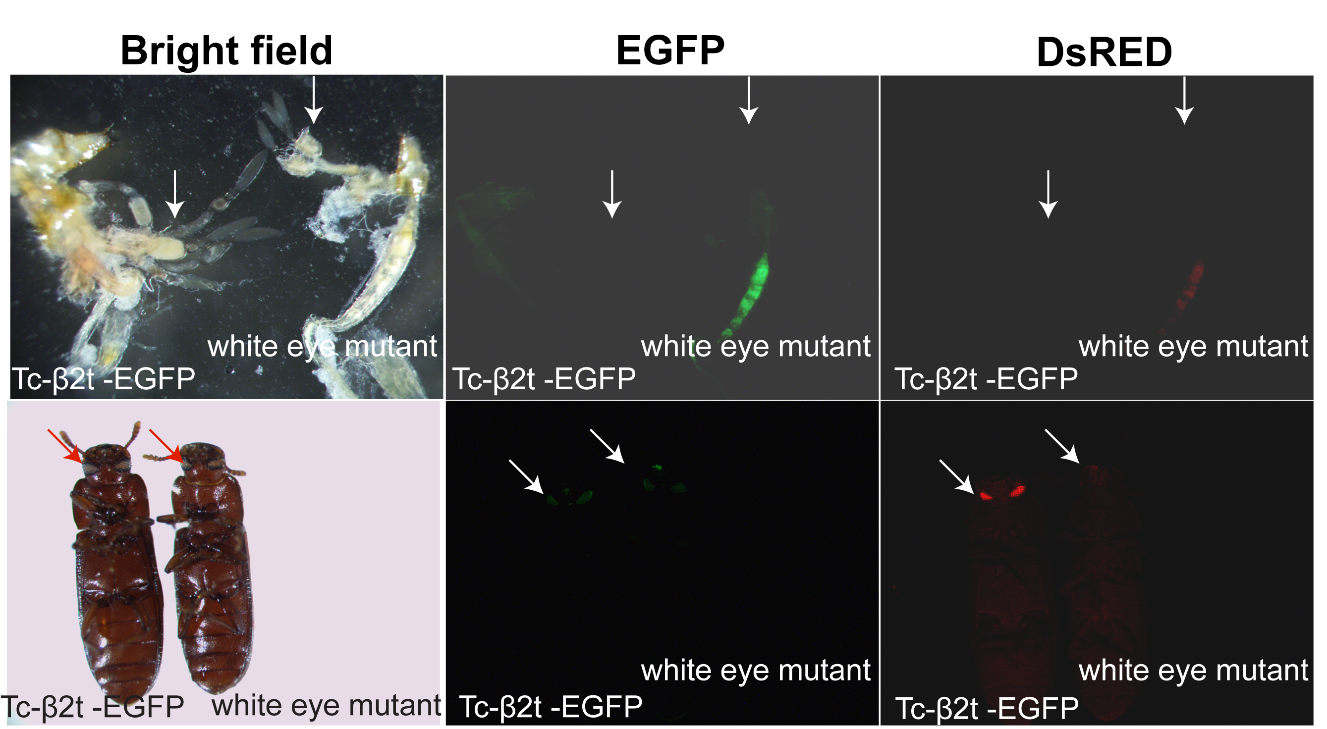
**

**Fig. S6. No EGFP fluorescence detected in the female gonad of Tc-β_2_t –EGFP** **beetles**. Ovaries dissected from the transgenic and white eye mutant beetles in bright field. No EGFP and DsRED expression was observed in transgenic ovaries in GFP and DsRED filters respectively. Arrows either indicate ovary or eye. Magnification (3X upper and 2x lower).


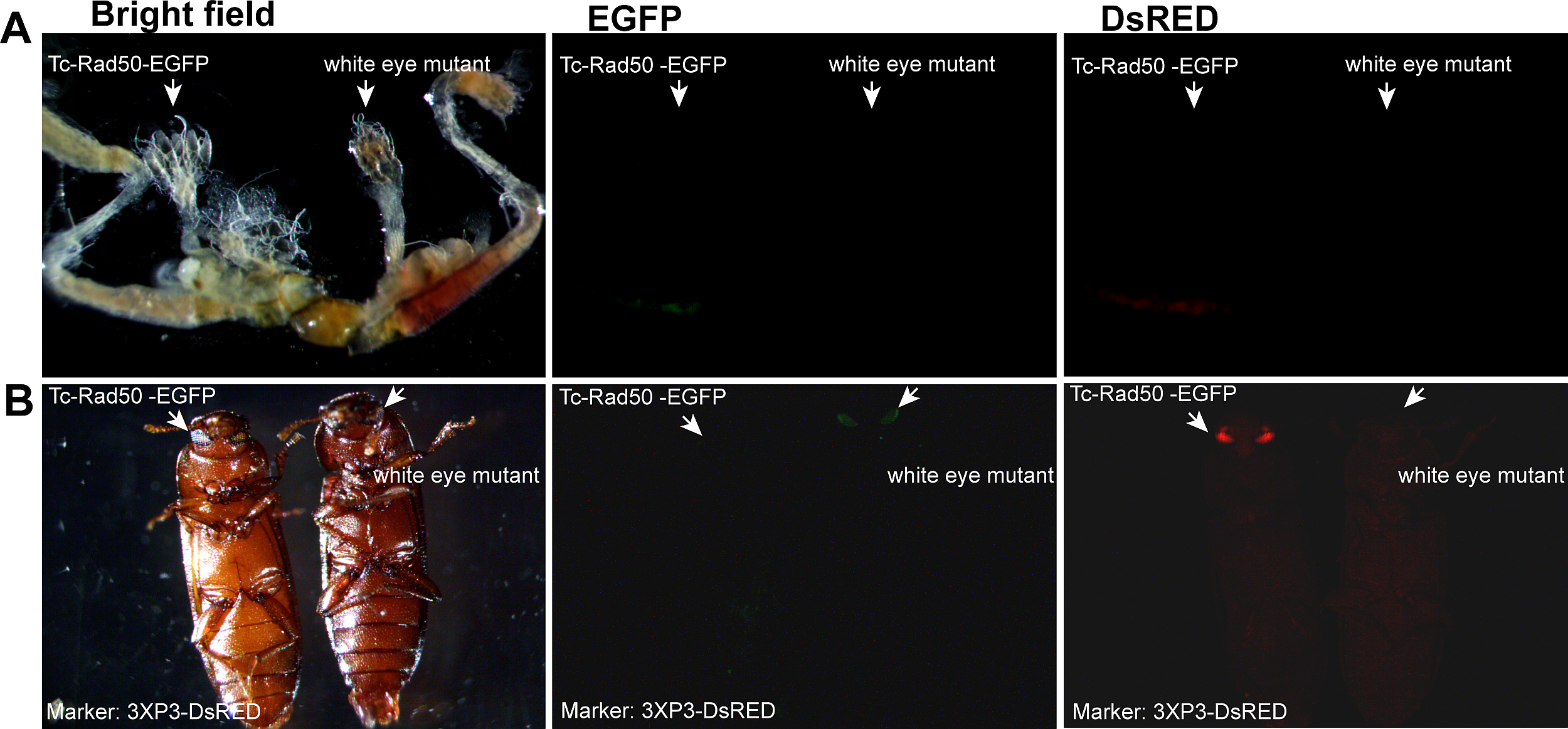


**Fig. S7. No EGFP fluorescence detected in the female gonad of Tc-Rad50 –EGFP#1 beetles.** Ovaries dissected from the transgenic and white-eyed mutant beetles in bright field. No EGFP and DsRED expression was observed in transgenic ovaries in GFP and DsRED filters respectively. Arrows indicate ovary and eye. Magnification A (3X), and B (2.5X).


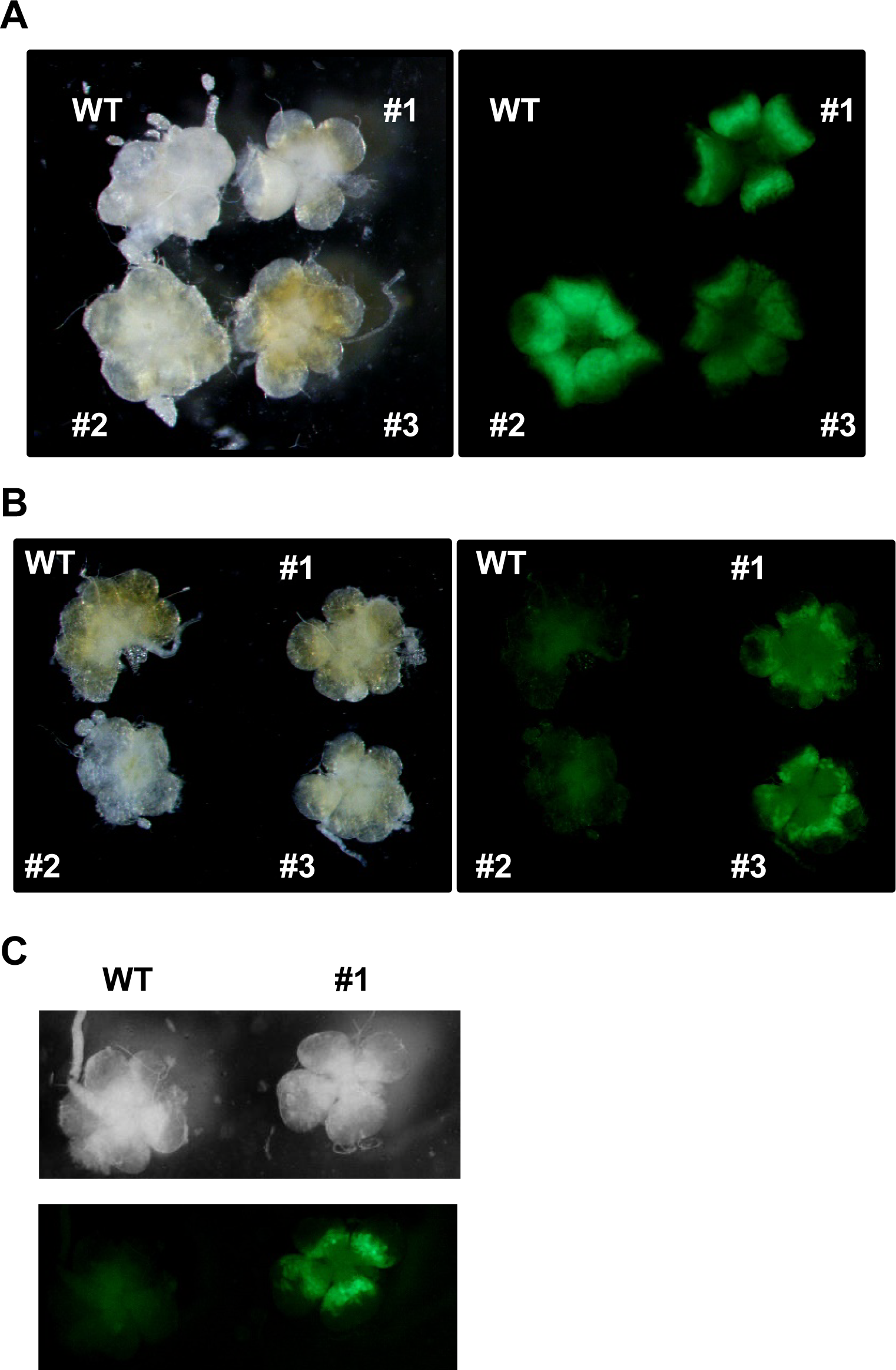


**Fig. S8. Expression of the EGFP reporter in the testes in transgenic beetle strains.** (**A**) Dissected testes from wild-type (WT) or transgenic beetle strains from Tc -β_2_t –EGFP lines (**B**) Tc -Rad50 -EGFP lines and (**C**) Tc –Eno -EGFP line, as viewed under white light or EGFP fluorescence. Magnification A (2.5 X), B(2X) and C(3X).


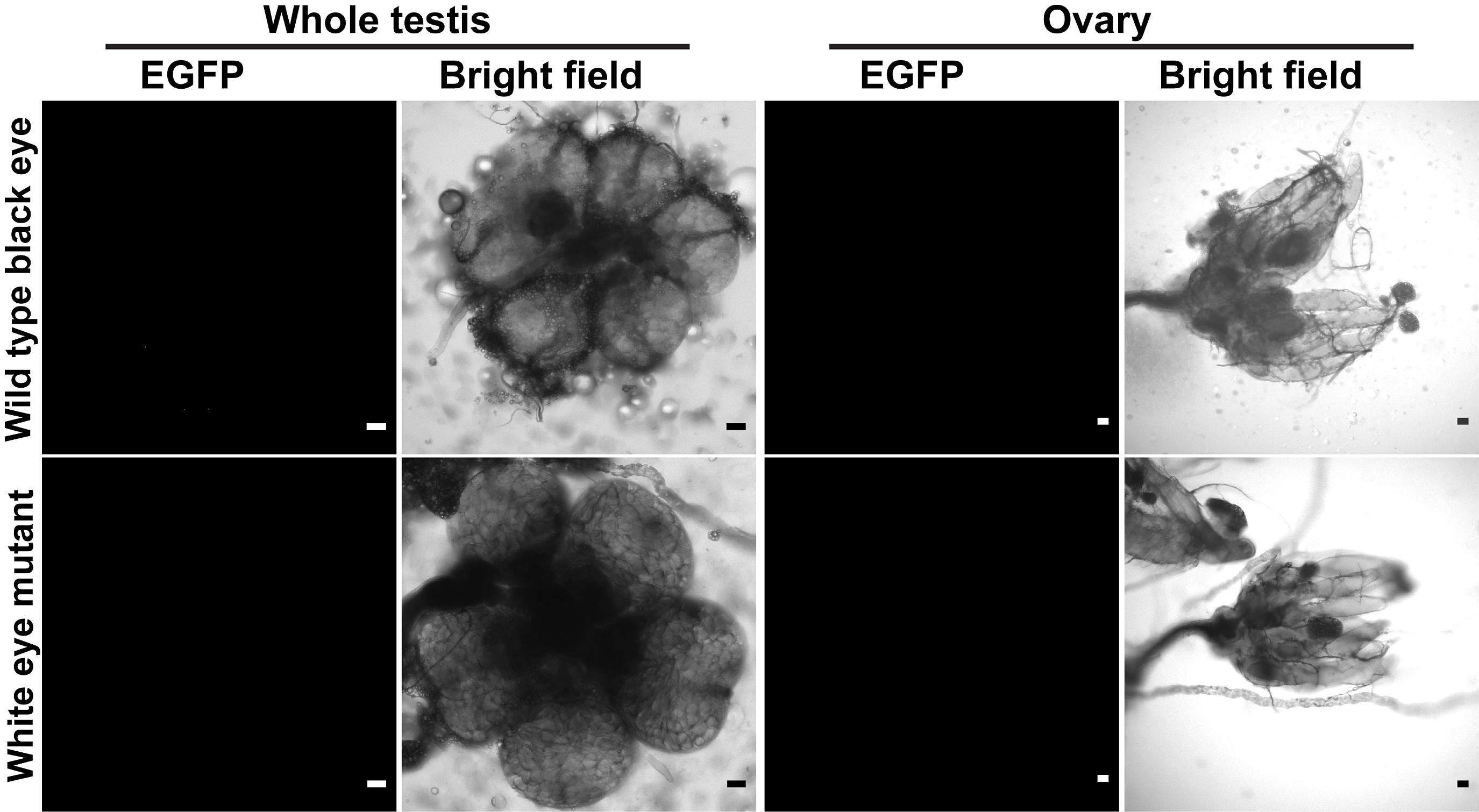


**Fig. S9.** **Confocal analysis of EGFP expression in wild type black eye and white-eyed mutant beetles’ gonads**. No EGFP fluorescence was detected in testes or ovaries from wild type black eye and white-eye mutant beetles. Scale bar indicates 50µm.

**Table S1. Primers used in this study.**

| **Primers sequence used for the RT-PCR amplification in white eyed mutant beetles and EGFP transgenic lines.** | |
| --- | --- |
| RpSL32 fwd | GGCACCAGTCTGACCGTTAT |
| RpSL32 Rev | GGGTAGCATGTGCTTCGTTT |
| Tub.β2 fwd | GCATGGACGAAATGGAGTTT |
| Tub.β2 rev | ATCGGTCCAATACACCGTTC |
| Rad50 ATPase fwd | GGAGGAGTACGAGGCGAAA |
| Rad50 ATPase rev | CGTTTGTTGGCTCTCCCTAA |
| Enolase fwd | GTTTGTTCCGAGCAGCTGTA |
| Enolase rev | TTGCGTTACCTCCAAGTGTG |
| EGFP fwd | GCAAGCTGACCCTGAAGTTC |
| EGFPrev | AAGTCGTGCTGCTTCATGTG |
|  |  |
| **Primers sequences used in Gibson assembly. Uppercase = gene-specific bases** | |
| Gibson β2t_fwd | cttatcgaatacgcgtacggAATTTACGAAAAACCGATGCATGGATTCAAAAC |
| Gibson β2t_rev | tcagatccgagatcggccggTTCCACGGACTGTTTATAGTGTGCTAAGCCA |
| Gib Rad 50 fwd | taaagcttatcgaatacgcgtacggGGTTTGGGGAGATTTTCCAG |
| Gib Rad 50 rev | cattgtcagatccgagatcggccggGTTTTTCAAATTGCTGGTTTTTG |
| Gib Enolase fwd | taaagcttatcgaatacgcgtacggGGTTTGGGGAGATTTTCCAG |
| Gib Enolase rev | cattgtcagatccgagatcggccggGTTATCACACTTAAATAATGGTTCC |
|  |  |
|  |  |
| **3' UTR Beetle_Confirm_Primers** |  |
|  |  |
| B2Tubulin 3' UTR F | CACAATCGAAGCAGGTATAC |
| Rad50 3'UTR F | GGTAGATGTCGCTCGCGTC |
| Enolase 3'UTR F | CAGAAACAATGACAGTGGC |
| Enolase #1 Genomic R | ATGTGATGCGTCCGG |
| B2Tubulin #1 Genomic R | CATTGTCAGATCCGAGATCGGCC |
| B2Tubulin #2 Genomic R | GCACTGAACATTGTCAGATCCGAGATC |
| Rad50 #1 Genomic R | CATTGTAGACGGAATGAAGTGG |
| Rad50 #2 Genomic R | CTTTATCCCTCAACTACAC |
| Rad50 #3 Genomic R | CTTGTTGCTATCGCATTTTG |
| Enolase #1 Genomic New R | CTGATAAATGCGCCGGCG |
| B2Tubulin #1 Genom R New | GACACTTCCCATATCCCTAG |
| B2Tubulin #2 Genom R New | GTAACGGTTAAAGCACAGC |
| Enolase #2 Genomic R | CATATTTAGACAGGCGTACGTG |
| B2Tubulin #3 Genomic R | CAATAGAGTAAAGCGGTAC |
| Rad50 #1 FINAL GEN R | TCAGATCATCAGAGAAGTTTAGAGTTT |
|  |  |
| **Inverse PCR** |  |
| iPCR 5’ 1 F | TGACACTTACCGCATTGACA |
| iPCR 5’ 2 F | TCCAAGCGGCGACTGAGATG |
| iPCR 5’ 1 R | GACGCATGATTATCTTTTACGTGA |
| iPCR 5’ 2 R | GCGATGACGAGCTTGTTGGTG |

**Table S2. Testis enriched genes in *T. castaneum* identified in Khan et al 2019.**

| **Testis enriched genes in *T. castaneum* identified in previous study Khan et al 2019 .** | | | | | | |  |  |  |  |
| --- | --- | --- | --- | --- | --- | --- | --- | --- | --- | --- |
|  |  |  | **5' -end cis-regulatory region** | | |  | **3' -end cis-regulatory region** | | |  |
|  | **Linkge group** | **5' -end gene** | **Start** | **end** | **length (nt)** | **Testis enriched genes** | **start** | **end** | **length (nt)** | **3' -end gene** |
| 1 | LG9 | TcasGA2_TC012420 | 7084800 | 7085299 | 499 | **TcasGA2_TC034328 /TC012421** | 7086500 | 7086899 | 399 | TcasGA2_TC034329 |
| 2 | LG8 | TcasGA2_TC002176 | 915000 | 929999 | 14999 | **TcasGA2_TC002214** | 933000 | 940999 | 7999 | TcasGA2_TC033927 |
| 3 | LG3 | TcasGA2_TC010503 | 15351000 | 15382999 | 31999 | **TcasGA2_TC010548** | 15387000 | 15387999 | 999 | TcasGA2_TC010502 |
| 4 | LG2 | TcasGA2_TC001290 | 13995000 | 14001999 | 6999 | **TcasGA2_TC000256** | 13986000 | 13994999 | 8999 | TcasGA2_TC034566 |
| ***Tc-rad50*** | LG8 | TcasGA2_TC006702 | 13080100 | 13080299 | 199 | **TcasGA2_TC006703** | 13082100 | 13082399 | 299 | TcasGA2_TC006704 |
| 6 | LG2 | TcasGA2_TC032056 | 6189000 | 6192999 | 3999 | **TcasGA2_TC032055** | 6188000 | 6188999 | 999 | TcasGA2_TC032054 |
| ***Tc-eno*** | LG9 | TcasGA2_TC034448 | 14323000 | 14323999 | 999 | **TcasGA2_TC011729** | 14321000 | 14321599 | 599 | TcasGA2_TC011730 |
| 8 | LG7 | TcasGA2_TC033610 | 4201400 | 4202199 | 799 | **TcasGA2_TC033611** | 4195100 | 4200499 | 5399 | TcasGA2_TC015919 |
| 9 | LG9 | TcasGA2_TC031242 | 10578000 | 10593999 | 15999 | **TcasGA2_TC011969** | 10597000 | 10600999 | 3999 | TcasGA2_TC012510 |
| 10 | LG2 | TcasGA2_TC032152 | 10266000 | 10273999 | 7999 | **TcasGA2_TC032153** | 10276000 | 10284999 | 8999 | TcasGA2_TC032154 |
| 11 | LG6 | TcasGA2_TC015681 | 4995000 | 4996999 | 1999 | **TcasGA2_TC015010/ TcasGA2_TC033421** | 5007000 | 5008999 | 1999 | TcasGA2_TC015011 |
| 12 | LG8 | TcasGA2_TC006316 | 7148300 | 7148499 | 199 | **TcasGA2_TC006317** | 7149100 | 7150699 | 1599 | TcasGA2_TC006318 |
| 13 | LG6 | TcasGA2_TC033455 | 8887000 | 8890999 | 3999 | **TcasGA2_TC033456** | 8892000 | 8901999 | 9999 | TcasGA2_TC033457 |
| 14 | LG4 | TcasGA2_TC007145 | 8674000 | 8675999 | 1999 | **TcasGA2_TC008334** | 8681000 | 8687999 | 6999 | TcasGA2_TC008335 |
| 15 | LG3 | TcasGA2_TC003592 | 8495000 | 8505999 | 10999 | **TcasGA2_TC002844** | 8511000 | 8597999 | 86999 | TcasGA2_TC002843 |
| 16 | LG4 | TcasGA2_TC007826 | 1096000 | 1101999 | 5999 | **TcasGA2_TC007828** | 1103000 | 1105999 | 2999 | TcasGA2_TC032743 |
| ***Tc-β_2_t*** | LG7 | TcasGA2_TC009803 | 8324100 | 8324699 | 599 | **TcasGA2_TC009035** | 8321800 | 8322799 | 999 | TcasGA2_TC009802 |
| 18 | LG9 | TC012339 is present within 3rd intron in opposite direction of TC034296 (close fly homolog Tango1 - Transport and Golgi organization 1 (CG11098) | | | | **TcasGA2_TC012339** |  |  |  |  |

**Table S3. B-tubulin protein sequences used to build alignment in Supplementary Fig. S1.**

>APIME Tubulin beta

MREIAHLQAGQCGNQIGAKFWEVISDEHGIDPTGTYHGDSDLQLERINVYYNEASGGKYV

PRAILVDLEPGTMDAVRSGPFGQIFRPDNFVFGQSGAGNNWAKGHYTEGAELVDSVLDVV

RKEAESCDCLQGVQLTHSLGGGTGAGMGTLLISKIREEYPDRIMMTFSVVPSPKVSDTVV

EPYNCTLSVHQLVENTDESYCIDNEALYDICFRTLKLTTPTYGDLNHLVSATLSGVTTCL

RFPGQLNADLRKLAVNMVPFPRLHFFIPGFAPLTSRGSQQYRALTVPELTQQMFDAKNMM

AACDPRHGRYLTVAAVFRGRMSMKEVDEQMLNIQNKNSSYFVEWIPSNVKTAVCDIPPRG

LKMSATFIGNSTAIQELFKRVSEQFTAMFRRKAFLHWYTGEGMDEMEFTEAESNMNDLVS

EYQQYQEATAEEEGEFDEEEEGEGEHP

>Dm Tubulin beta chain (betaTub56D)

MLIGARPIHRVYDGVHGWPKPRSRAQTCFWEIISDEHGIDATGAYHGDSDLQLERINVYY

NEASGGKYVPRAVLVDLEPGTMDSVRSGPFGQIFRPDNFVFGQSGAGNNWAKGHYTEGAE

LVDSVLDVVRKEAESCDCLQGFQLTHSLGGGTGSGMGTLLISKIREEYPDRIMNTYSVVP

SPKVSDTVVEPYNATLSVHQLVENTDETYCIDNEALYDICFRTLKLTTPTYGDLNHLVSL

TMSGVTTCLRFPGQLNADLRKLAVNMVPFPRLHFFMPGFAPLTSRGSQQYRALTVPELTQ

QMFDAKNMMAACDPRHGRYLTVAAIFRGRMSMKEVDEQMLNIQNKNSSYFVEWIPNNVKT

AVCDIPPRGLKMSATFIGNSTAIQELFKRISEQFTAMFRRKAFLHWYTGEGMDEMEFTEA

ESNMNDLVSEYQQYQEATADEDAEFEEEQEAEVDEN

>Dm Tubulin beta-2 chain (betaTub85D)

MREIVHIQAGQCGNQIGGKFWEVISDEHCIDATGTYYGDSDLQLERINVYYNEATGAKYV

PRAILVDLEPGTMDSVRSGAFGQIFRPDNFVFGQSGAGNNWAKGHYTEGAELVDSVLDVV

RKESEGCDCLQGFQLTHSLGGGTGSGMGTLLISKIREEYPDRIMNTFSVVPSPKVSDTVV

EPYNATLSVHQLVENTDETYCIDNEALYDICFRTLKLTTPTYGDLNHLVSATMSGVTTCL

RFPGQLNADLRKLAVNMVPFPRLHFFMPGFAPLTSRGSQQYRALTVPELTQQMFDAKNMM

AACDPRHGRYLTVAAIFRGRMSMKEVDEQMLNIQNKNSSFFVEWIPNNCKTAVCDIPPRG

LKMSATFIGNSTAIQELFKRVSEQFTAMFRRKAFLHWYTGEGMDEMEFTEAESNMNDLVS

EYQQYQEATADEEGEFDEDEEGGGDE

>Bm Tubulin beta Bombyx mori

MREIVHIQAGQCGNQIGAKFWEVISDEHGIDATGAYSGDSDLQLERINVYYNEASGGKYV

PRAVMVDLEPGTMDSVRSGPFGQIFRPDNFVFGQSGAGNNWAKGHYTEGAELVDSVLDVV

RKEAEGCDCLQGFQLTHSLGGGTGSGMGTLLISKIREEYPDRIMNTFSVVPSPKVSDTVV

EPYNATLSVHQLVENTDETYCIDNEALYDICFRTLKLTTPTYGDLNHLVSATMSGVTTCL

RFPGQLNADLRKLAVNMVPFPRLHFFMPGFAPLTSRGSQQYRALSVPELTQQMFDAKNMM

AACDPRHGRYLTVAAVFRGRMSMKEVDEQMLNIQNKNSSYFVEWIPNNVKTAVCDIPPRG

LKMSATFIGNTTAIQELFKRISEQFTAMFRRKAFLHWYTGEGMDEMEFTEAESNMNDLFR

STSSIKTPRLMMKGNLMKR

>De Tubulin beta Drosophila erecta

MREIVHIQAGQCGNQIGGKFWEVISDEHCIDATGTYYGDSDLQLERINVYYNEATGAKYV

PRAILVDLEPGTMDSVRSGAFGQIFRPDNFVFGQSGAGNNWAKGHYTEGAELVDSVLDVV

RKESEGCDCLQGFQLTHSLGGGTGSGMGTLLISKIREEYPDRIMNTFSVVPSPKVSDTVV

EPYNATLSVHQLVENTDETYCIDNEALYDICFRTLKLTTPTYGDLNHLVSATMSGVTTCL

RFPGQLNADLRKLAVNMVPFPRLHFFMPGFAPLTSRGSQQYRALTVPELTQQMFDAKNMM

AACDPRHGRYLTVAAIFRGRMSMKEVDEQMLNIQNKNSSFFVEWIPNNCKTAVCDIPPRG

LKMSATFIGNSTAIQELFKRVSEQFTAMFRRKAFLHWYTGEGMDEMEFTEAESNMNDLVS

EYQQYQEATADEEGEFDEDEEGGGDE

>De Tubulin beta Drosophila erecta

MREIVHIQAGQCGNQIGAKFWEIISDEHGIDATGAYHGDSDLQLERINVYYNEASGGKYV

PRAVLVDLEPGTMDSVRSGPFGQIFRPDNFVFGQSGAGNNWAKGHYTEGAELVDSVLDVV

RKEAESCDCLQGFQLTHSLGGGTGSGMGTLLISKIREEYPDRIMNTYSVVPSPKVSDTVV

EPYNATLSVHQLVENTDETYCIDNEALYDICFRTLKLTTPTYGDLNHLVSLTMSGVTTCL

RFPGQLNADLRKLAVNMVPFPRLHFFMPGFAPLTSRGSQQYRALTVPELTQQMFDAKNMM

AACDPRHGRYLTVAAIFRGRMSMKEVDEQMLNIQNKNSSYFVEWIPNNVKTAVCDIPPRG

LKMSATFIGNSTAIQELFKRISEQFTAMFRRKAFLHWYTGEGMDEMEFTEAESNMNDLVS

EYQQYQEATADEDAEFEEEQEAEVDEN

>De Tubulin beta Drosophila ananassae

MREIVHIQAGQCGNQIGAKFWEIISDEHGIDATGAYHGDSDLQLERINVYYNEASGGKYV

PRAVLVDLEPGTMDSVRSGPFGQIFRPDNFVFGQSGAGNNWAKGHYTEGAELVDSVLDVV

RKEAESCDCLQGFQLTHSLGGGTGSGMGTLLISKIREEYPDRIMNTYSVVPSPKVSDTVV

EPYNATLSVHQLVENTDETYCIDNEALYDICFRTLKLTTPTYGDLNHLVSLTMSGVTTCL

RFPGQLNADLRKLAVNMVPFPRLHFFMPGFAPLTSRGSQQYRALTVPELTQQMFDAKNMM

AACDPRHGRYLTVAAIFRGRMSMKEVDEQMLNIQNKNSSYFVEWIPNNVKTAVCDIPPRG

LKMSATFIGNSTAIQELFKRISEQFTAMFRRKAFLHWYTGEGMDEMEFTEAESNMNDLVS

EYQQYQEATADEDAEFEEEQEAEVDEN

>Da Tubulin beta Drosophila ananassae

MREIVHIQAGQCGNQIGGKFWEVISDEHCIDATGTYYGDSDLQLERINVYYNEATGAKYV

PRAILVDLEPGTMDSVRSGAFGQIFRPDNFVFGQSGAGNNWAKGHYTEGAELVDSVLDVV

RKESEGCDCLQGFQLTHSLGGGTGSGMGTLLISKIREEYPDRIMNTFSVVPSPKVSDTVV

EPYNATLSVHQLVENTDETYCIDNEALYDICFRTLKLTTPTYGDLNHLVSATMSGVTTCL

RFPGQLNADLRKLAVNMVPFPRLHFFMPGFAPLTSRGSQQYRALTVPELTQQMFDAKNMM

AACDPRHGRYLTVAAIFRGRMSMKEVDEQMLNIQNKNSSFFVEWIPNNCKTAVCDIPPRG

LKMSATFIGNSTAIQELFKRVSEQFTAMFRRKAFLHWYTGEGMDEMEFTEAESNMNDLVS

EYQQYQEATADEEGEFDEDEEGGGDE

>Dm_betaTub60D-PA

MREIVNLQAGQCGNQIGAKFWEIISEEHGIDSNGIYVGDSDLQLERVSVYYNEASAVTRS

SGGKYVPRAILLDLEPGTMESVRSGPYGQLFRPDNFVYGQSGAGNNWAKGHYTEGAELVD

NVLDVVRKECENCDCLQGFQLTHSLGGGTGSGMGTLLISKIREEYPDRIMNTYSVVPSPK

VSDTVVEPYNATLSIHQLVENTDETYCIDNEALYDICFRTLKVSNPSYGDLNHLVSLTMS

GVTTCLRFPGQLNADLRKLAVNMVPFPRLHFFMPGFAPLTSRGSQQYRALTVPELTQQMF

DAKNMMAACDPRHGRYLTVAAVFRGRMSMKEVDEQMLAVQNKNSSYFVEWIPNNVKTAVC

DIPPKGLKMSSTFIGNTTAIQELFKRISEQFSAMFRRKAFLHWYTGEGMDEMEFTEAESN

MNDLVSEYQQYQEATADDEFDPEVNQEEVEGDCI

>Nv_tubulin beta chain [Nasonia vitripennis]

MREILNIQAGQCGNQIGSRFWEVISGEHGIQPDGSYEGTNDLQLDKISVFFNETSFKRYIPRTVIVDLEP

GTIDAIRSSAFGDLFRPDNFIFGQNGASNNWAKGHYTEGAEIVDQVLDVVRREAENCDCIQGFQLTHSLG

GGTGSGMGTLLLSKIRDEYPDRIINAYSIVPSPKVQDTVVGPYNVLLSIQQLVNLTDSTFCIDNQALYEI

CLRTLELPSPTYTDLNHLVSLTMSGVTTCLRFPGQLNADLRKLAVNMVPFPRLHFFLTGFAPLISRASKI

YNGLSVSELTQQMFDARNVMAACDPRNGRYLTVAAIFRGQMSMKEVEDEMLNIQNKNSPYFVDWIPNNVK

TAVCDIPPKGLSISGTFLGNSTVIHEVFKRVSAQFTLMFKRKAFLHWFTGEGMEENEFTDAHADLLDLIA

EYQQYETMNGPRDAPDEDVEVVEEELRD

>Nv_tubulin beta-1 chain [Nasonia vitripennis]

MREIVHIQAGQCGNQIGAKFWEIISDEHGIDPTGAYHGDSDLQLERINVYYNEASGGKYVPRAILVDLEP

GTMDSVRSGPFGQIFRPDNFVFGQSGAGNNWAKGHYTEGAELVDSVLDVVRKEAESCDCLQGFQLTHSLG

GGTGSGMGTLLISKIREEYPDRIMNTYSVVPSPKVSDTVVEPYNATLSVHQLVENTDETYCIDNEALYDI

CFRTLKLSTPTYGDLNHLVSLTMSGVTTCLRFPGQLNADLRKLAVNMVPFPRLHFFMPGFAPLTSRGSQQ

YRALSVPELTQQMFDAKNMMAACDPRHGRYLTVAAIFRGRMSMKEVDEQMLNIQNKNSSYFVEWIPNNVK

TAVCDIPPRGLKMSATFIGNSTAIQELFKRISEQFTAMFRRKAFLHWYTGEGMDEMEFTEAESNMNDLVS

EYQQYQEATADEDAEFDEEAEQEVDEN

>Nv_tubulin beta-4 chain [Nasonia vitripennis]

MREIVHLQAGQCGNQIGAKFWEVISDEHGIDPTGAYHGDSDLQLERINVYYNEATGGKYVPRAILVDLEP

GTMDAVRSGPFGQIFRPDNFVFGQSGAGNNWAKGHYTEGAELVDSVLDVVRKEAESCDCLQGFQLTHSLG

GGTGAGMGTLLISKIREEYPDRIMMTFSVVPSPKVSDTVVEPYNCTLSVHQLVENTDESYCIDNEALYDI

CFRTLKLTTPTYGDLNHLVCATLSGVTTCLRFPGQLNADLRKLAVNMVPFPRLHFFIPGFAPLTSRGSQQ

YRSLTVPELTQQMFDAKNMMAACDPRNGRYLTVAAVFRGRMSMKEVDEQMLNIQNKNSSYFVEWIPSNVK

TAVCDIPPRGLKMSATFIGNSTAIQELFKRVSEQFTAMFRRKAFLHWYTGEGMDEMEFTEAESNMNDLVS

EYQQYQEATAEEDTEFDEDEGENEGN

>Ag_b2t

MREIVHLQTGQCGNQIGAKFWEVISNEHGIDATGAFQGDCGDLQLERINVYYNEASGGKYVPRAILVDLE

PGTMDSVRSGPYGQLFRPDNFAFGQSGAGNNWAKGHYTEGAELVDSVLDIVRKEAEGCDCLQGFQLTHSL

GGGTGSGMGTLLISKIREEYPDRIMNTFSIVPSPKVSDTVVEPYNATLSVHQLIENTDETYCIDNEALYD

ICFRTLK

>Bm_beta-tubulin [Bombyx mori]

MREIVHLQAGQCGNQIGAKFWEIISEEHGIDPTGVYRGTSDLQLERISVYYNEASVATAESGGKYVPRAI

LLDLEPGTMDAVRSGAYGQLFRPDNFVFGQSGAGNNWAKGHYTEGAELVDAVLDVVRKECENCDCLQGFQ

LTHSLGGGTGSGMGTLLISKIREEYPDRIMNTYSVVPSPKVSDTVVEPYNAVLSIHQLVENTDETYCIDN

EALYDICYRTLKVPNPTYGDLNHLVSLTMSGVTTCLRFPGQLNADLRNWSVNMVPFPRLHFFMPGFAPLT

SRGSQQYRALTVPELTQQMFDAKNMMAACDPRHGRYLTVAAIFRGRMSMKEVDEQMLSIQNKNSSFFVEW

IPNNVKTAVCDIPPKGLKMSSTFIGNTTAIQELFKRISEQFSAMFRRKAFLHWYTGEGMDEMEFNEAESN

VNDLVSEYQQYQEATAEDDTEFDQEDLEELAQDEHHD

>Co_tubulin beta-2 chain [Colletotrichum orchidophilum]

MREIVHLQTGQCGNQIGAAFWQNISGEHGLDSNGVYNGTSELQLERMSVYFNEASGNKYVPRAVLVDLEP

GTMDAVRAGPFGQLFRPDNFVFGQSGAGNNWAKGHYTEGAELVDQVLDVVRREAEGCDCLQGFQITHSLG

GGTGAGMGTLLISKIREEFPDRMMATFSVVPSPKVSDTVVEPYNATLSVHQLVENSDETFCIDNEALYDI

CMRTLKLSNPSYGDLNHLVSAVMSGVTTCLRFPGQLNSDLRKLAVNMVPFPRLHFFMVGFAPLTSRGAHS

FRAVSVPELTQQMFDPKNMMAASDFRNGRYLTCSAIFRGKVAMKDVEDQMRNVQNKNSSYFVEWIPNNVQ

TALCSIPPRGLKMSSTFVGNSTAIQELFKRVGEQFTAMFRRKAFLHWYTGEGMDEMEFTEAESNMNDLVS

EYQQYQDAGVDEEEEEYEEEVPLEEEV

>HUMAN Tubulin beta-2A chain

MREIVHIQAGQCGNQIGAKFWEVISDEHGIDPTGSYHGDSDLQLERINVYYNEAAGNKYV

PRAILVDLEPGTMDSVRSGPFGQIFRPDNFVFGQSGAGNNWAKGHYTEGAELVDSVLDVV

RKESESCDCLQGFQLTHSLGGGTGSGMGTLLISKIREEYPDRIMNTFSVMPSPKVSDTVV

EPYNATLSVHQLVENTDETYSIDNEALYDICFRTLKLTTPTYGDLNHLVSATMSGVTTCL

RFPGQLNADLRKLAVNMVPFPRLHFFMPGFAPLTSRGSQQYRALTVPELTQQMFDSKNMM

AACDPRHGRYLTVAAIFRGRMSMKEVDEQMLNVQNKNSSYFVEWIPNNVKTAVCDIPPRG

LKMSATFIGNSTAIQELFKRISEQFTAMFRRKAFLHWYTGEGMDEMEFTEAESNMNDLVS

EYQQYQDATADEQGEFEEEEGEDEA

>GORGO_Tubulin beta chain

MREIVHIQAGQCGNQIGAKFWEVISDEHGIDPTGSYHGDSDLQLERINVYYNEATGNKYV

PRAILVDLEPGTMDSVRSGPFGQIFRPDNFVFGQSGAGNNWAKGHYTEGAELVDSVLDVV

RKESESCDCLQGFQLTHSLGGGTGSGMGTLLISKIREEYPDRIMNTFSVMPSPKVSDTVV

EPYNATLSVHQLVENTDETYSIDNEALYDICFRTLKLTTPTYGDLNHLVSATMSGVTTCL

RFPGQLNADLRKLAVNMVPFPRLHFFMPGFAPLTSRGSQQYRALTVPELTQQMFDSKNMM

AACDPRHGRYLTVAAIFRGRMSMKEVDEQMLNVQNKNSSYFVEWIPNNVKTAVCDIPPRG

LKMSATFIGNSTAIQELFKRISEQFTAMFRRKAFLHWYTGEGMDEMEFTEAESNMNDLVS

EYQQYQDATADEQGEFEEEEGEDEA

>Dm Tubulin alpha-2 chain

MRECISVHIGQAGVQIGNACWELYCLEHGIQPDGHMPSDKTVGGGDDSFSTFFSETGAGK

HVPRAVFVDLEPTVVDEVRTGTYRQLFHPEQLITGKEDAANNYARGHYTIGKEIVDVVLD

RIRKLADQCTGLQGFLVFHSFGGGTGSGFTSLLMERLSVDYGKKSKLEFSIYPAPQVSTA

VVEPYNSILTTHTTLEHSDCAFMVDNEAIYDICRRNLDIERPTYMNLNRLIGQIVSSITA

SLRFDGALNVDLTEFQTNLVPYPRIHFPLATYAPVISVEKAYHEQLTVAEITNACFEPAN

QMVKCDPRRGKYMACCMLYRGDVVPKDVNAAIATIKTKRSIQFVDWCPTGFKVGINYQPP

TVVPGGDLAKVQRAVCMLSNTTAIAEAWARLDHKFDLMYAKRAFVHWYVGEGMEEGEFAE

AREDLAALEKDYEEVGIDSTTELGEDEEY

>Bm Tubulin alpha chain

MRECISVHIGQAGVQIGNACWELYCLEHGIQPDGQMPSDKTLGGGDDSFNTFFSETGAGK

HVPRAVFVDLEPTVVDEVRTGTYRQLFHPEQLITGKEDAANNYARGHYTIGKEIVDVVLD

RIRKLADQCTGLQGFLVFHSFGGGTGSGFTSLLMERLSVDYGKKSKLEFSIYPAPQVSTA

VVEPYNSILTTHTTLEHSDCAFMVDNEAIYDICRRNLDIERPTYTNLNRLIGQIVSSITA

SLRFDGALNVDLTEFQTNLVPYPRIHFPLATYAPVISAEKAYHEQLTVAEITNACFEPAN

QMVKCDPRHGKYMACCMLYRGDVVPKDVNAAIATIKTKRTIQFVDWCPTGFKVGINYQPP

TVVPGGDLAKVQRAVCMLSNTTAIAEAWARLDHKFDLMYAKRAFVHWYVGEGMEEGEFSE

AREDLAALEKDYEEVGVDSTEGELDEENEY

>Tc Tubulin alpha chain

MRECISVHIGQAGVQIGNACWELYCLEHGIQPDGQMPSDKTIGGGDDSFNTFFSETGAGK

HVPRAVFVDLEPTVVDEVRTGTYRQLFHPEQLITGKEDAANNYARGHYTIGKEIVDVVLD

RIRKLADQCTGLQGFLVFHSFGGGTGSGFTSLLMERLSVDYGKKSKLEFSIYPAPQVSTA

VVEPYNSILTTHTTLEHSDCAFMVDNEAIYDICRRNLDIERPTYTNLNRLIGQIVSSITA

SLRFDGALNVDLTEFQTNLVPYPRIHFPLATYAPVISAEKAYHEQLTVAEITNACFEPAN

QMVKCDPRHGKYMACCMLYRGDVVPKDVNAAIATIKTKRTIQFVDWCPTGFKVGINYQPP

TVVPGGDLAKVQRAVCMLSNTTAIAEAWARLDHKFDLMYAKRAFVHWYVGEGMEEGEFSE

AREDLAALEKDYEEVAVDSIEGEGDEGDEY

>Aa Tubulin alpha chain

MRECISVHVGQAGVQIGNACWELYCLEHGIQPDGQMPSDKTIGGGDDSFNTFFSETGAGK

HVPRAVFVDLEPTVVDEVRTGTYRQLFHPEQLITGKEDAANNYARGHYTIGKEIVDVVLD

RIRKLADQCTGLQGFLIFHSFGGGTGSGFTSLLMERLSVDYGKKSKLEFAIYPAPQVSTA

VVEPYNSILTTHTTLEHSDCAFMVDNEAIYDICRRNLDIERPTYTNLNRLIGQIVSSITA

SLRFDGALNVDLTEFQTNLVPYPRIHFPLVTYAPVISAEKAYHEQLSVAEITNACFEPAN

QMVKCDPRHGKYMACCMLYRGDVVPKDVNAAIATIKTKRTIQFVDWCPTGFKVGINYQPP

TVVPGGDLAKVQRAVCMLSNTTAIAEAWARLDHKFDLMYAKRAFVHWYVGEGMEEGEFSE

AREDLAALEKDYEEVGMDSGEGEGEGAEEY

>Aa Tubulin alpha chain

MRECISVHVGQAGVQIGNACWELYCLEHGIQPDGQMPSDKTIGGGDDSFNTFFSETGAGK

HVPRAVFVDLEPTVVDEVRTGTYRQLFHPEQLITGKEDAANNYARGHYTIGKEIVDLVLD

RIRKLADQCTGLQGFLIFHSFGGGTGSGFTSLLMERLSVDYGKKSKLEFAIYPAPQVSTA

VVEPYNSILTTHTTLEHSDCAFMVDNEAIYDICRRNLDIERPTYTNLNRLIGQIVSSITA

SLRFDGALNVDLTEFQTNLVPYPRIHFPLVTYAPVISAEKAYHEQLSVAEITNACFEPAN

QMVKCDPRHGKYMACCMLYRGDVVPKDVNAAIATIKTKRTIQFVDWCPTGFKVGINYQPP

TVVPGGDLAKVQRAVCMLSNTTAIAEAWARLDHKFDLMYAKRAFVHWYVGEGMEEGEFSE

AREDLAALEKDYEEVGMDSGEGEGEGAEEY

>HUMAN Tubulin alpha-1B chain

MRECISIHVGQAGVQIGNACWELYCLEHGIQPDGQMPSDKTIGGGDDSFNTFFSETGAGK

HVPRAVFVDLEPTVIDEVRTGTYRQLFHPEQLITGKEDAANNYARGHYTIGKEIIDLVLD

RIRKLADQCTGLQGFLVFHSFGGGTGSGFTSLLMERLSVDYGKKSKLEFSIYPAPQVSTA

VVEPYNSILTTHTTLEHSDCAFMVDNEAIYDICRRNLDIERPTYTNLNRLISQIVSSITA

SLRFDGALNVDLTEFQTNLVPYPRIHFPLATYAPVISAEKAYHEQLSVAEITNACFEPAN

QMVKCDPRHGKYMACCLLYRGDVVPKDVNAAIATIKTKRSIQFVDWCPTGFKVGINYQPP

TVVPGGDLAKVQRAVCMLSNTTAIAEAWARLDHKFDLMYAKRAFVHWYVGEGMEEGEFSE

AREDMAALEKDYEEVGVDSVEGEGEEEGEEY

>GORGO Tubulin alpha chain

MRECISIHVGQAGVQIGNACWELYCLEHGIQPDGQMPSDKTIGGGDDSFNTFFSETGAGK

HVPRAVFVDLEPTVVDEVRTGTYRQLFHPEQLITGKEDAANNYARGHYTIGKEIVDLVLD

RIRKLADLCTGLQGFLIFHSFGGGTGSGFASLLMERLSVDYGKKSKLEFAIYPAPQVSTA

VVEPYNSILTTHTTLEHSDCAFMVDNEAIYDICRRNLDIERPTYTNLNRLIGQIVSSITA

SLRFDGALNVDLTEFQTNLVPYPRIHFPLATYAPVISAEKAYHEQLSVAEITNACFEPAN

QMVKCDPRHGKYMACCMLYRGDVVPKDVNAAIATIKTKRTIQFVDWCPTGFKVGINYQPP

TVVPGGDLAKVQRAVCMLSNTTAIAEAWARLDHKFDLMYAKRAFVHWYVGEGMEEGEFSE

AREDLAALEKDYEEVGVDSVEAEAEEGEEY

>Tc_bTub1

MREIVHIQAGQCGNQIGAKFWEIISDEHGIDPTGAYHGDSDLQLERINVYYNEASGGKY

VPRAILVDLEPGTMDSVRSGPFGQIFRPDNFVFGQSGAGNNWAKGHYTEGAELVDSVLDVVRKEAESCDC

LQGFQLTHSLGGGTGSGMGTLLISKIREEYPDRIMNTYSVVPSPKVSDTVVEPYNATLSVHQLVENTDET

YCIDNEALYDICFRTLKLTTPTYGDLNHLVSLTMSGVTTCLRFPGQLNADLRKLAVNMVPFPRLHFFMPG

FAPLTSRGSQQYRALTVPELTQQMFDAKNMMAACDPRHGRYLTVAAVFRGRMSMKEVDEQMLNIQNKNSS

YFVEWIPNNVKTAVCDIPPRGLKMSATFIGNSTAIQELFKRISEQFTAMFRRKAFLHWYTGEGMDEMEFT

EAESNMNDLVSEYQQYQEATADEDAEFDEEQEAEVDEN

> Tc_bTub2

MREIVHIQAGQCGNQIGAKFWEVISDEHGIDPTGTYHGDSDLQLERINVYYNEATGGKYVPRAILVDLEP

GTMDSVRSGPFGQIFRPDNFVFGQSGAGNNWAKGHYTEGAELVDSVLDVVRKEAEGCDCMQGFQLTHSLG

GGTGSGLGTLLISKIREEYPDRIMNTFSVVPSPKVSDTVVEPYNATLSVHQLVENTDETYCIDNEALYDI

CFRTLKLTTPTYGDLNHLVSATMSGVTTCLRFPGQLNSDLRKLAVNMVPFPRLHFFMPGFAPLTSRGSQQ

YRALTVPELVMQMFDAKNMMAACDPRHGRYLTVAAIFRGRMSMKEVDEQMLNIQNKNSSYFVEWIPNNVK

TAVCDIPPRGLKMSSTFIGNSTCIQELFKRISEQFTAMFRRKAFLHWYTGEGMDEMEFTEAESNMNDLVA

EYQQYQDATAEEEGEFDEEEEGDNEGEN

> Tc_bTub3

MREIVHLQAGQCGNQVGAKFWEVISEEHGIDPSGIYLGESDLQLERINVYYNEATVASRSNGGKYVPRAI

LLDLEPGTMDAVRSGIYGRLFRPDNFVFGQSGAGNNWAKGHYTEGAELVDAVLDVVRKEAENCDCLQGFQ

LTHSLGGGTGSGMGTLLISKIREEYPDRIMNTYSVMPSPKVSDTVVEPYNATLSVHQLVENTDETYCIDN

EALYDICFRTLKVPNPSYGDLNHLVSLTMSGVTTCLRFPGQLNADLRKLAVNMVPFPRLHFFMPGFAPLT

SRGSQQYRALTVPELTQQMFDAKNMMAACDPRHGRYLTVAAVFRGRMSMKEVDEQMLAVQNKNSSYFVEW

IPNNVKTAVCDIPPVGLKMSSTFIGNTTAIQELFKRISEQFAAMFRRKAFLHWYTGEGMDEMEFTEAESN

MNDLVSEYQQYQEATADEEYEAEEEAAADDFNC

>Tc_bTub4

MREIVHLQVGQCGNQIGSRFWHTISKEHGINPVGKYVGDNDLQLERIEVYFNEAENANYVPRAVLVDLEP

GTMDMVRAGPLGTLFNPNNFVFAQSGAGNNWATGHYTEGAEICDNVLDVIQHEVERCDCFQGFQFTHSLG

GGTGSGFGTLLTSKVREEYPDRMVTSFSVVPSPKVSEVVLEPYNATLSVQQLIENTDQTQCIDNEALYDI

CFNTLKKSSPKYDDLNHLISLTMAGVTTCFRFPGQLNADLRKLSVNMVPFPRLHFFVPGFAPLTAPGSVA

YRKLSVKDLTEQMFSPRNIMAACNPANGRYLTVATIFRGPVSMKEVDHVMFETQQKNSAYFVEWIPHNIQ

TAVCDISSPHAPISSTFIANTTAIQELFKRIQEQFSAMFRRKAYVHWYQAEGMDLQEFTEAESNLNDLIS

EYQQYQDAEVDEEYGDEDETEEDKFEEET

>Dm_beta-Tubulin at 56D, isoform B

MREIVHIQAGQCGNQIGAKFWEIISDEHGIDATGAYHGDSDLQLERINVYYNEASGGKYVPRAVLVDLEP

GTMDSVRSGPFGQIFRPDNFVFGQSGAGNNWAKGHYTEGAELVDSVLDVVRKEAESCDCLQGFQLTHSLG

GGTGSGMGTLLISKIREEYPDRIMNTYSVVPSPKVSDTVVEPYNATLSVHQLVENTDETYCIDNEALYDI

CFRTLKLTTPTYGDLNHLVSLTMSGVTTCLRFPGQLNADLRKLAVNMVPFPRLHFFMPGFAPLTSRGSQQ

YRALTVPELTQQMFDAKNMMAACDPRHGRYLTVAAIFRGRMSMKEVDEQMLNIQNKNSSYFVEWIPNNVK

TAVCDIPPRGLKMSATFIGNSTAIQELFKRISEQFTAMFRRKAFLHWYTGEGMDEMEFTEAESNMNDLVS

EYQQYQEATADEDAEFEEEQEAEVDEN

>Nv_tubulin beta chain [Nasonia vitripennis]

MREIVHLQAGQCGNQIGAKFWEVISEEHGIDQSGIYHGDSDLQLERIDVYYNEASVATSTNGGKYVPRAI

LLDLEPGTMDAVRSGAYGKLFRPDNFVFGQSGAGNNWAKGHYTEGAELVDSVLDVVRKECENCDCLQGFQ

LTHSLGGGTGSGMGTLLISKIREEYPDRIMNTYSVMPSPKVSDTVVEPYNATLSVHQLVENTDETYCIDN

EALYDICFRTLKVSNPSYGDLNHLVSLTMSGVTTCLRFPGQLNADLRKLAVNMVPFPRLHFFMPGFAPLT

SRSMQQYSTLSVPELTQQMFDAKNMMAACDPRHGRYLTVAAVFRGRMSMKEVDEQMLSVQNKNSSYFVEW

IPNNVKTAVCDIPPKGLKMSSTFIGNTTAIQELFKRISEQFTAMFRRKAFLHWYTGEGMDEMEFTEAESN

MNDLVSEYQQYQEATTEEDFETEDAGDDFETCDQE

>Nv_tubulin beta chain [Nasonia vitripennis]

MDSVRSGPFGQIFRPDNFVFGQSGAGNNWAKGHYTEGAELVDSVLDVVRKEAESCDCLQGFQLTHSLGGG

TGSGMGTLLISKIREEYPDRIMNTYSVVPSPKVSDTVVEPYNATLSVHQLVENTDETYCIDNEALYDICF

RTLKLSTPTYGDLNHLVSLTMSGVTTCLRFPGQLNADLRKLAVNMVPFPRLHFFMPGFAPLTSRGSQQYR

ALSVPELTQQMFDAKNMMAACDPRHGRYLTVAAIFRGRMSMKEVDEQMLNIQNKNSSYFVEWIPNNVKTA

VCDIPPRGLKMSATFIGNSTAIQELFKRISEQFTAMFRRKAFLHWYTGEGMDEMEFTEAESNMNDLVSEY

QQYQEATTEEDFETEDAGDDFETCDQE

>Ag_AGAP010929-PA [Anopheles gambiae beeta tub1]

MREIVHIQAGQCGNQIGAKFWEIISDEHGIDATGGYHGDSDLQLERINVYYNEASGGKYVPRAVLVDLEP

GTMDSVRSGPFGQIFRPDNFVFGQSGAGNNWAKGHYTEGAELVDSVLDVVRKEAESCDCLQGFQLTHSLG

GGTGSGMGTLLISKIREEYPDRIMNTYSVVPSPKVSDTVVEPYNATLSVHQLVENTDETYCIDNEALYDI

CFRTLKLTTPTYGDLNHLVSLTMSGVTTCLRFPGQLNADLRKLAVNMVPFPRLHFFMPGFAPLTSRGSQQ

YRALTVPELTQQMFDAKNMMAACDPRHGRYLTVAAVFRGRMSMKEVDEQMLNIQNKNSSYFVEWIPNNVK

TAVCDIPPRGLKMSATFIGNSTAIQELFKRISEQFTAMFRRKAFLHWYTGEGMDEMEFTEAESNMNDLVS

EYQQYQEATADEDAEFDEEQEAEVDEN

>Ag_AGAP005293-PB [Anopheles gambiae str. PEST]

MREIVHLQAGQCGNQIGSKFWEIISDEHGIDPTGHYHGDSDLQLERIDVYYTEVNGNKYVPRAVLVDLEP

GTMDSVRQSPYGALFRPDNFVYAQSGAGNNWAKGHYTEGAELVDNVLDVIRKETESCDCLQGFQLAHSLG

GGTGSGMGTLLISKIREEYPDRIMNTFSVVPSPKVSDTVVEPYNATLSIHQLVENTDDTFCIDNEALYDI

CFRTLKLSSPTYGDLNHLVSVTMSGVTTCLRFPGQLNADLRKLAVNMVPFPRLHFFMPGFAPLTAKGSQQ

YRALTVPELTQQMFDAKNMMTACDPRHGRYLTCAAIFRGSMSMKEVDQQMLNIQSKYSSYFVEWIPNNVK

VAVCDIAPRGLKMSATFIGNTTAIQEIFKRISEQFTAMFRRKAFLHWYTGEGMDEMEFTEAESNMNDLIS

EYQQYQDAEVEDYDEMEEIPEEEQQQQQE

>Aa_tubulin beta-1 chain [Aedes aegypti]

MREIVHIQAGQCGNQIGAKFWEIISDEHGIDATGAYHGDSDLQLERINVYYNEASGGKYVPRAVLVDLEP

GTMDSVRSGPFGQIFRPDNFVFGQSGAGNNWAKGHYTEGAELVDSVLDVVRKEAESCDCLQGFQLTHSLG

GGTGSGMGTLLISKIREEYPDRIMNTYSVVPSPKVSDTVVEPYNATLSVHQLVENTDETYCIDNEALYDI

CFRTLKLTTPTYGDLNHLVSLTMSGVTTCLRFPGQLNADLRKLAVNMVPFPRLHFFMPGFAPLTSRGSQQ

YRALTVPELTQQMFDAKNMMAACDPRHGRYLTVAAVFRGRMSMKEVDEQMLNIQNKNSSYFVEWIPNNVK

TAVCDIPPRGLKMSATFIGNSTAIQELFKRISEQFTAMFRRKAFLHWYTGEGMDEMEFTEAESNMNDLVS

EYQQYQEATADEDAEFDEEQEAEVDEN

>Aa_beta-4 tubulin [Aedes aegypti]

MREIVHIQAGQCGNQIGAKFWEIISDEHGIDATGAYHGDSDLQLERINVYYNEASGGKYVPRAVLVDLEP

GTMDSVRSGPFGQIFRLDNFVFGQSGAGNNWAKGHYTEGAELVDSVLDVVRKEAESCDCLQGFQLTHSLG

GGTGSGMGTLLISKIREEYPDRIMNTYSVVPSPKVSDTVVEPYNATLSVHQMVENTDETYCIDNEALYDI

CFRTLKLTTPTYGDLNHLVSLTMSGVTTCLRFPGQLNADLRKLAVNMVPFPRLHFFMPGFAPLTSRGSQQ

YRALTVPELTQQMFDAKNMMAACDPRHGRYLTVAAVFRGRMSMKEVDEQMLNIQNKNSSYFIEWIPNNVK

TAVCDIPPRGLKMSATFIGNSTAIQELFKRISEQFTAMFRRKAFLHWYTGEGMDEMEFTEAESNMNDLVS

EYQQYQEATADEDAEFDEEQEAEVDEN

>Aa_tubulin beta-3 chain [Aedes aegypti] XP_001656026.2

MREIVHLQAGQCGNQIGAKFWEIISEEHGIDATGIYHGESDLQLERVSVYYNEASAVSRSSGGKYVPRAI

LLDLEPGTMEAVRSGAYGKLFRPDNFVFGQSGAGNNWAKGHYTEGAELVDAVLDVVRKECENCDCLQGFQ

LTHSLGGGTGSGMGTLLISKIREEYPDRIMNTYSVVPSPKVSDTVVEPYNATLSIHQLVENTDETYCIDN

EALYDICFRTLKVPNPSYGDLNHLVSLTMSGVTTCLRFPGQLNADLRKLAVNMVPFPRLHFFMPGFAPLT

SRGSQQYRALTVPELTQQMFDAKNMMAACDPRHGRYLTVAAVFRGRMSMKEVDEQMLAVQNKNSSYFVEW

IPNNVKTAVCDIPPKGLKMSSTFIGNTTAIQELFKRISEQFSAMFRRKAFLHWYTGEGMDEMEFTEAESN

MNDLVSEYQQYQEATADDEFEQEECADEMEGECV

>Ld_tubulin beta chain XP_023018856.1

MREIVHIQAGQCGNQIGAKFWEVISDEHGIDPTGTYHGDSDLQLERINVYYNEATGGKYVPRAVLVDLEP

GTMDSVRSGPFGQIFRPDNFVFGQSGAGNNWAKGHYTEGAELVDSVLDVVRKEAEGCDCMQGFQLTHSLG

GGTGSGLGTLLISKIREEYPDRIMNTFSVVPSPKVSDTVVEPYNATLSVHQLVENTDETYCIDNEALYDI

CFRTLKLTTPTYGDLNHLVSATMSGVTTCLRFPGQLNSDLRKLAVNMVPFPRLHFFMPGFAPLTSRGSQQ

YRALTVPELVLQMFDAKNMMAACDPRHGRYLTVAAIFRGRMSMKEVDEQMLNIQNKNSSYFVEWIPNNVK

TAVCDIPPRGLKMSSTFIGNSTCIQELFKRISEQFTAMFRRKAFLHWYTGEGMDEMEFTEAESNMNDLVS

EYQQYQDATAEEEGEFDEEEEADEGEN

>Ld_tubulin beta-1 chain [Leptinotarsa decemlineata] XP_023026756.1

MREIVHIQAGQCGNQIGAKFWEIISDEHGIDPTGAYHGDSDLQLERINVYYNEASGGKYVPRAILVDLEP

GTMDSVRSGPFGQIFRPDNFVFGQSGAGNNWAKGHYTEGAELVDSVLDVVRKEAESCDCLQGFQLTHSLG

GGTGSGMGTLLISKIREEYPDRIMNTYSVVPSPKVSDTVVEPYNATLSVHQLVENTDETYCIDNEALYDI

CFRTLKLTTPTYGDLNHLVSLTMSGVTTCLRFPGQLNADLRKLAVNMVPFPRLHFFMPGFAPLTSRGSQQ

YRALTVPELTQQMFDAKNMMAACDPRHGRYLTVAAVFRGRMSMKEVDEQMLNIQNKNSSYFVEWIPNNVK

TAVCDIPPRGLKMSATFIGNSTAIQELFKRISEQFTAMFRRKAFLHWYTGEGMDEMEFTEAESNMNDLVS

EYQQYQEATADEDAEFDEDQEAEVDEN

>Ld_tubulin beta chain-like XP_023012462.1

MDAVRSGPFGQIFRPDNFVFGQSGAGNNWAKGHYTEGAELVDSVLDVVRKEAETCDCLQGFQLTHSLGGG

TGSGMGTLLISKIREEYPDRIMNTYSVVPSPKVSETVVEPYNATLSVHQLVENTDETYCIDNEALYDICF

RTLKLTSPTYGDLNHLVSLTMSGVTTCLRFPGQLNADLRKLAVNMVPFPRLHFFMPGFAPLTSMGSKQYR

ALTVPELTQQMFDAKNMMAACDPRKMMLSPNKNTQSLRRSNLAYFKVQKL

>Bm_tubulin beta chain [Bombyx mori] XP_004923508.1

MREIVHIQAGQCGNQIGAKFWEVISDEHGIDPTGTYHGDSDLQLERINVYYNEATGGKYVPRAILVDLEP

GTMDSVRSGPFGQIFRPDNFVFGQSGAGNNWAKGHYTEGAELVDSVLDVVRKEAEGCDCLQGFQLTHSLG

GGTGAGLGTLLISKIREEYPDRIMNTFSIVPSPKVSDTVVEPYNATLSVHQLVENTDESYCIDNEALYDI

CFRTLKLTTPTYGDLNHLVSATMSGVTTCLRFPGQLNADLRKLAVNMVPFPRLHFFIPGFAPLTSRGSQQ

YRALTVPELTQQMFDAKNMMAACDPRHGRYLTVAAVFRGRMSMKEVDEQMMNIQNKNSSYFVEWIPNNVK

TAVCDIPPRGLKMSATFIGNSTAIQELFKRISEQFTAMFRRKAFLHWYTGEGMDEMEFTEAESNMNDLVS

EYQQYQDATAEEEGEFDEEEEGGDEGD

>Bm_beta-tubulin [Bombyx mori] NP_001036887.1

MREIVHLQAGQCGNQIGAKFWEIISDEHGIDPTGAYHGDSDLQLERINVYYNEASGGKYVPRAILVDLEP

GTMDSVRSGPFGQIFRPDNFVFGQSGAGNNWAKGHYTEGAELVDSVLDVVRKESESCDCLQGFQLTHSLG

GGTGSGMGTLLISKIREEYPDRIMNTYSVVPSPKVSDTVVEPYNATLSVHQLVENTDETYCIDNEALYDI

CFRTLKLSTPTYGDLNHLVSLTMSGVTTCLRFPGQLNADLRKLAVNMVPFPRLHFFMPGFAPLTSRGSRQ

YRALTVPELTQQMFDAKNMMAACDPRHGRYLTVAAIFRGRMSMKEVDEQMLNIQNKNSSYFVEWIPNNVK

TAVCDIPPRGLKMAATFIGNSTAIQELFKRISEQFTAMFRRKAFLHWYTGEGMDEMEFTEAESNMNDLVS

EYQQYQEATADEDAEFDEEQEQEIEEH

>Bm_beta-tubulin [Bombyx mori] NP_001036964.1

MREIVHIQAGQCGNQIGAKFWEIISDEHGIDPTGAYHGDSDLQLERINVYYNEASGGKYVPRAILVDLEP

GTMDSVRSGPFGQIFRPDNFVFGQSGAGNNWAKGHYTEGAELVDSVLDVVRKEAESCDCLQGFQLTHSLG

GGTGSGMGTLLISKIREEYPDRIMNTYSVVPSPKVSDTVVEPYNATLSVHQLVENTDETYCIDNEALYDI

CFRTLKLSTPTYGDLNHLVSLTMSGVTTCLRFPGQLNADLRKLAVNMVPFPRLHFFMPGFAPLTSRGSQQ

YRALTVPELTQQMFDAKNMMAACDPRHGRYLTVAAIFRGRMSMKEVDEQMLNIQNKNSSYFVEWIPNNVK

TAVCDIPPRGLKMAATFIGNSTAIQELFKRISEQFTAMFRRKAFLHWYTGEGMDEMEFTEAESNMNDLVS

EYQQYQEATADEDAEFDEEQEQEIEDN

>Bm_beta-tubulin [Bombyx mori] BAA32102.1

MREIVHIQAGQCGNQIGAKFWEIISDEHGIDPTGAYHGDSDLQLERINVYYNEASGGKYVPRAILVDLEP

GTMDSVRSGPFGQIFRPDNFVFGQSGAGNNWAKGHYTEGAELVDSVLDVVRKEAESCDCLQGFQLTHSLG

GGTGSGMGTLLISKIREEYPDRIMNTYSVVPSPKVSDTVVEPYNATLSVHQLVENTDETYCIDNEALYDI

CFRTLKLSTPTYGDLNHLVSLTLSGVTTCLRFPGQLNADLRKLAVNMVPFPRLHFFMPGFAPLTSRGSQQ

YRALTVPELTQQMFDAKNMMAACDSRHGRYLTVAAIFRGRMSMKEVDEQMLNIQNKNSSYFVEWIPNNVK

TAVCDIPPRGLKMAATFIGNSTAIQELFKRISEQFTAMFRRKAFLHWYTGEGMDEMEFTEAESNMNDLVS

EYQQYQEATASEDAEFDEEQEQEIEDN

>Bm_beta-tubulin [Bombyx mori] NP_001036965.1

MREIVHIQAGQCGNQIGAKFWEVISDEHGIDATGAYSGDSDLQLERINVYYNEASGGKYVPRAVMVDLEP

GTMDSVRSGPFGQIFRPDNFVFGQSGAGNNWAKGHYTEGAELVDSVLDVVRKEAEGCDCLQGFQLTHSLG

GGTGSGMGTLLISKIREEYPDRIMNTFSVVPSPKVSDTVVEPYNATLSVHQLVENTDETYCIDNEALYDI

CFRTLKLTTPTYGDLNHLVSATMSGVTTCLRFPGQLNADLRKLAVNMVPFPRLHFFMPGFAPLTSRGSQQ

YRALSVPELTQQMFDAKNMMAACDPRHGRYLTVAAVFRGRMSMKEVDEQMLNIQNKNSSYFVEWIPNNVK

TAVCDIPPRGLKMSATFIGNTTAIQELFKRISEQFTAMFRRKAFLHWYTGEGMDEMEFTEAESNMNDLIS

EYQQYQDATADDEGEFDEEAEEGLEE

>Bm_beta-tubulin isoform X1 [Bombyx mori] XP_021203550.1

MREIVHLQAGQCGNQIGAKFWEIISDEHGIDPTGAYHGDSDLQLERINVYYNEASGGKYVPRAILVDLEP

GTMDSVRSGPFGQIFRPDNFVFGQSGAGNNWAKGHYTEGAELVDSVLDVVRKESESCDCLQGFQLTHSLG

GGTGSGMGTLLISKIREEYPDRIMTXXXXXXXPKVSDTVVEPYNATLSVHQLVENTDETYCIDNEALYDI

CFRTLKLSTPTYGDLNHLVSLTMSGVTTCLRFPGQLNADLRKLAVNMVPFPRLHFFMPGFAPLTSRGSRQ

YRALTVPELTQQMFDAKNMMAACDPRHGRYLTVAAIFRGRMSMKEVDEQMLNIQNKNSSYFVEWIPNNVK

TAVCDIPPRGLKMAATFIGNSTAIQELFKRISEQFTAMFRRKAFLHWYTGEGMDEMEFTEAESNMNDLVS

EYQQYQEATADEDAEFDEEQEQEIEEH

>Bm_beta-tubulin [Bombyx mori] NP_001093079.1

MREIINLQVGSCGNQIGGKFWEVISDEHGIDPSGCYHGDSDLQLERINVYYNEASAGKYVPRTVLIDLKP

ATMDAVRSGPFGCLFRPDNFVYGQNCAANNWAKGHYTEGVEILESALDVIRREAEGCDCLQGFQMSHSLG

GGTGSGLGTLLVNRIREEYPDRIILTFSVFPSPRVSDCVVEPYNTTLAVNQLVENTDHTFCLDNEALYDI

CFRTLKLTTPTYGDLNHLVCATMSGITTCLRFPGQLNADLRKLAVNMVPFPRLHFYTPGFAPLTSRGAQQ

YRALTVPELTLQMFDAKNMMVACDPRHGRYLTVATIFRGRMSMKEVDEQLANIQNKNSTYFVEWIPNNCK

IAVCDIPPRGLKMASTFIGNTTAIQTIFKRVSEQFVAMFRRKAFLHWYTGEGMDEMEFTEAESNMNDLIS

EYQQYQDATADDEGEFDEEAEEGLEE

>Bm_b4t

MREIVHVQVGRCGNQIGSKFWEVISDEHGIDPCGRYHGDSDLQLERINVYYNEAFGAKYVPRAVLVDLEP

STMDSIRGGPYGSLYRPDNVVCGASGAGNNWAKGHYTEGADLLETVLDVVRKEAEGCDCLQGFQLVHSLG

GGTGSGMGTLLLANLTDEYPDRITATYSVVPSPTVSETVVEPYNATLSVNQLIENSIQSYCIDNEALYYI

CHRTLKLMAPTYGALNHLVSLTMSGVTTCLRFPGQLNADLRKLAVNMIPFPRLHFFMPGFAPLTSRGSQQ

YRALTVPELTQQMFDAKNMMAACDPHRGRYLTVATVFRGRMSMKEIDEQILNVQKKNKDFFVEWIPNNVQ

TAVCDIPPRGMKMSATFIGNTTAIQEIFKRISEQFAAMFSRKAFLHWYTGEGMEEGDFAEADNNVSDLLS

EYQQYQDATIDQEFEDEEEVEEQNDDSDEQ

>Aa_b2t

MREIVHIQAGQCGNQIGAKFWEVISDEHGIDATGAYCGDSDLQLERINVYYNEATGGKYVPRAVLVDLEP

GTMDSVRAGPFGQLFRPDNFVFGQSGAGNNWAKGHYTEGAELVDSVLDVVRKESEGCDCLQGFQLTHSLG

GGTGSGMGTLLISKIREEYPDRIMNTFSVVPSPKVSDTVVEPYNATLSVHQLVENTDESYCIDNEALYDI

CFRTLKLTTPTYGDLNHLVSATMSGVTTCLRFPGQLNADLRKLAVNMVPFPRLHFFMTGFAPLTSRGSQQ

YRALTVPELTQQMFDAKNMMAACDPRHGRYLTVAAIFRGRMSMKEVDEQMLNIQSKNSSYFVEWIPNNVK

TAVCDIPPRGLKMSSTFIGNSTAIQEIFKRIAEQFTAMFRRKAFLHWYTGEGMDEMEFTEAESNMNDLVS

EYQQYQEATADEEGEFDEEEEGGEE

**Table S4. Rad50 protein sequences used to build alignment in Supplementary Fig. S2.**

>XP_969783.1 PREDICTED: DNA repair protein RAD50 [Tribolium castaneum]

MATLERLQISGVRSFGPNEEHCQTIKFATPLTLILGQNGSGKTTIIEAIKYVCTAELPQGTNGGQGFVNDPKMSSLTTTR

GQIKLRVVDSKNNAVTISRIMELTQTAINTQRFSSKGATIRIVKPNGDDSSISGRCADITNECCQIMNVSSSILNNVVFC

HQENSAWPLDEGKKVKEKFDEIFDAQQSNKCVEIYRKLLKEKQEKIKLLKLELEYKKEKKEQVDKDKRVLQDKEAKLESF

DAEIAQKMTKLQPVKKRIDEIIDLEKVLSELERDLATKEATKNGLVEEQKTIKKNLAFEFEGTDQELQDKIKSFENERQK

DETLIQDLVKRQNDIETKKREINEAVQKTQSDLGRLNQQKSHNLKKCCERKKLFDQALDKFRVKISNFDDNTGAKHAINQ

LKSALNERQDDLGTLIEEKELDLMSLQATLDDVRAKSIKTQQIISSKTREVTDCEQKIESVSLELAALASSDEFLQDYTK

RLEAISKTIFNLNNSFRETEESERMEASRSQVTTLEHNLDGLEREYRILLQNDTVEGKIESERHLIMEKQREINKIKSKH

AESLRELFGTQSPDCNLKDAVLAKQKSADLALQTLNEQIARKQKDVTTLEVQLQNQIERIGSCQRDLKTGREKISQVCNG

RDFDEVLTRCSEKKERLQRDKGNYTSVKIIYNQYIKKFEEERPCCPVCETNFSGKTTVVGKIITTLKSKLDKVPQQLAKV

ETELVEEEALYNKLQQLKVVNDEITVLTREKLPELESGLNEIKRDYEAKKTDLEALKQQTCGPLKTLEISKSVISDVSTL

DQNQADVDKSQKTVEKLKRELALVPSQKSKQEVEAEIDSTKAELSELRRKIEAQTKKINSHKDRLQQLTQERNALFEKQI

RMKDSMQNRPNLEAQLNEANEKLQTLRGEIVAKKTELEDLEDELGKIETQKKNLVQENKTVVEKERNQVAMFGNLVHNIE

KLEGEIEDYVREGVDEKLAALETTLGEFRQKEATLDHTKTKILNLLSEKREALAKHELRFRTLQSNVTLREKKVLELDLV

LKIEDLRKLIGGHDYRTVYDEKCELIRQKESHEKTINSITGEKNALLETVDDLRLKLAKPDWKNAYSNYMTKLYELKLAE

CVVKDLFKYITVLEKAILEFHATRMKQINKTIREMWREIYRGNDVDYIEIKAEHAGSTTANRKRTYNYRVVQVKKGVELE

MRGRCSAGQKVLACLIIRMALAETLSANCGILALDEPTTNLDRENIFSLCEALARIVESRQKEKNFQLVVITHDEEFINA

LTRAQGVPFFYRVSRNQDGFSVIKKEYAL

>XP_021712276.1 DNA repair protein RAD50 [Aedes aegypti]

MSTISKLEIRGIRSFGVESGDVQKIKFQSPLTLIVGQNGCGKTTIIECLKYGLTGEVPPGTDRGKAFVHDPKIFSTVESM

GQVKLMVTDFTGNRVTATRSMKVSQKGRGQQPKFETLDSVVTMENVATGEKTTLSRPRAADINNEMCDAMGVSKAIINNV

IFCHQEDSNWPLEEPKELKKKFDAIFGTTEYNRVIEKLIKISKEYNDRQKEKAGDLKLLENIKSQAEVKHLQLLKVDKSR

QDKLAVVDSLENSLKPIYERLDQIAKIERDFSKLKQQEIEFKSKINTKEDQQNQIRSKIQNLFKGSFHELEIEIQNFKQS

KSSKLLELREAESDLSSRKAQEKVLQSKIQDFESRRIHLNTKRGQEQDLCGDRSKKMVALCDRLKLPAVGDFEAGGADVE

SALKAIKHGIRSEETAVQTLSKSHDEADHKAQKAIDKLREDKTKLEADFRMKGQMVADFVREKAKTQSEIAAIQRSAETL

KKIVTEIEKLEKDYENQKSNSNVEGMRRQLAEKKIKREELQAKLDRVEEQISALDAVAAKATELHLKEQQFNAKESEFKR

LRNKHTENLKRLFPSKTIDTNYKRNVQTLYDDLQRQIKQLNESMRKSQATVTEMETTRRSQKRELDRIERELTENKEKIY

TVCQGNPFEEVLAKLKDKISKNNLEHGELRSAEVLYKKYISRIEDDKCCPVCHKGMDGADVQDISSELSDEIRRLPEKIE

ALERMLKADQKKYDQLLALQPYSERIAKQTEEIPKLKEQLEETERKLVQASSDLEECQMSILEPNSSVQLINSILGDMSI

LDESVRDIDRMKKGIENLKIEVAVKTPEGTTASLDDIKLEREALRGELRVERISIDAMQNKIDEETERLNNLHQRYNQMK

EKKIQLQESVQSLDQKKAKELELGEKIASCQREMDETENKLGPVKQNLITEEKSKAKAKEENRANLNKAQAELEELKRMD

AEIVRLCKELDALSRLNLAEEIQKIKRKLQESNEEMKKIIAAIDEKTYQIDSLKKDVMNQDMIERDYLDNRDLMKLKDET

TSLKQGLDALMQSIGDMDAPNVAQERNKLLEQRDAIQSTKSQTTGQIAELENQSKALKKELDRSEFKNAVKNYLKTYSES

VVLKKMISDILKYRNALEWALMKYHAEKMEQINRSIYSLWRDIYRGNDIDYIRIKTEDESKPDKVTDKRRQYHYRVVQAK

NDVEIDMRGRCSAGQKVLASLIIRMALAETFSNNCGVMALDEPTTNLDRENIASLCESLRRIVTERENGNFLLVIITHDE

DFVTKLEKFDTYYRVSRDHDGKSIIKEEQL

>XP_005167995.1 DNA repair protein RAD50 [Danio rerio]

MSKIEKMSILGVRSFGVEDKDKQVISFFSPLTVLVGPNGAGKTTIIECLKYITSGDFPPGSKGNTFVHDPKDAHETDVRA

QIRLQFRDVNGDAVAVQRSMQCTQKGKKTEFKTLEGVITRIKHGEKVSLSSKCAEIDREMISSLGVSRAVLNHVIFCHQE

ESNWPLSEGKALKQKFDEIFSATRYIKVLETLRTLRQKQTNTVKSCQMELKYLKQNKDKAQEIRELLSTKETQLASSKES

VNRIEGQIDPLERRLNDIESSLGKVMKLDNDIKALDSRKKQMEDDNRELEEKMEQVFQGSDDQLQDMYQNHQRTVKEKEK

RLVECQRELERAGRECQRMNRIKSELLVEQGRLQLEADRHTQNIKKRDTQVKTLASFLELEGYDRTPLSERQLQSFYRQI

KERLDQDSEALNQTMHDMQQKETQKQHNIDDLRDKKTGLERTIELKKDLQAKKQQELKNIKSDLQKLEGSSNRLQELDTE

LQKAERELDNAVQACTVDSLKVEVTELLKEKAQLDQAQRKLDQEMEMLNTHTTARAQMDMMKKTKMDKEEQVRKIKSRHN

EELVSLLGHFPNKKELEDWIYSKSREIKSTREQITKMNKELASGEQKKSHYTAEIKRKEEQLAKYEERLFNVCGSQDFQS

DLSKLEDELEKCSKQRAMLAGATAVYSQFISQLTEEGDPCCPVCQRVFPSEAELQDVINDMQSKLRLVPDKLKNTEHDLK

RKERRRDEMMTLKPIRQSLVELQEKELPELRNQLQRVNRDIEKLKGDIEEQETLLCTLVSEEETAKACLQDISLMDRFQL

DLKDVERKIAQHAAKLQGVDLSRTMQQVSQEKQETQHRLDTTSSKIELKRKLIQDQQEQIQALRSSVNEIRGEKLQISSN

MQKRQQLEEQCVEFSTEIQTLHRDIRDAKEQASPLAATLEKLQQEKQDLVERRRKKQEEGQEKINAIKEKMKNITLFEKE

ITKYIEEGKDSYKEQKETELQEMDKQLHEAEKQREKTNKDMGNIRQDIDTQKVQERWLQDNLTLRKRVEELKEVSRKREA

LIKEMGNMQVLQLRNERREVERKLEDLKKNRSVALGRQKGYEDEILRFRKELNEDQYCRAEDLYRDKMIVMRTTELANKD

LDIYYKALDQTIMRFHSMKMEEINKIIRDLWRSTYRGQDIEYVEIRSDVDENASAGVKRRTYNYRVVMVKGDAALDMRGR

CSAGQKVLASLIIRLALAETFCLNCGILALDEPTTNLDRENIESLAHALVEIIKSRSRQRNFQLLVITHDEDFVELLGRS

NYVEHFYRIRKNQDQCSEISKCSINSLNSYLH

>XP_026298578.1 DNA repair protein RAD50 [Apis mellifera]

MSRIRRLSIRGIRNFGDEKEEALIKFSRPLTLILGPNGTGKTTIIEALKFATCGEFPPGSDRGKSFIHDPTLSTTSSVRG

VIRAEIIDVVGNTYTICRTIESTKANVTRFKTLDSALSRINKDTKEVISITNRCTNVDTELTLAMGVSKPILDYVIFCHQ

EDLSWPFQDGKKLKERFDEIFDSAKFNKALESIMKLIKDLKQRVNILKEQKQNYQLIVNEVIDKETKLEDNKKRLENSKI

KIEEFNKELEPVIQKIKKLEKMDVEYKDLQSEQKRKKAEYDMFKQQLDNLKEDLQNIFEGTTEKLLKEIESYDEKLIGKI

NEIDEFKKKLRDIAEKESTISNKLANERVTNGSLRQQIKDHEKKVILRNQILNESLSSWNLNIVDSNVSELEVIAFTKRL

QEKMRELEHKLEENKIKREEEEKELQKVVDALRDKYLKLDTEKNLKENEIIETRKEIDKMKLDIMQLSAESNKLNFIESK

LQKVQIRIQQLNDSINVDTMKKEIDDKIKIRNEMDILLNTIDEEITLLLKQSSLQTELELNKSTLVIKEKEIEKLKNKHE

EKIISLLDIKDLSQIKLKNNFDIVQKELINEIESINQKIRTEEHQIATLETTISHIEHELQNKKKEINLNKEKISLICSY

KNFDEVLLLQSKKVKDLQDKRGMFAHQSAAYKEYMKQLRNTNPCCPLCHRGFDKIETITTLLKEMETEIENHPNRLKECE

KELIIQQEKYDKMLQLKPVVERIIQLEEIELEKLMSNLEKFKNKLNESRTIAIELKTKKSNPEKKLVMCKDIISDVMLWD

TYIDDIFKLKQIIDNFHIRMTAAGIKTKRSLQEAQTEREELKISLKKIRENIERLQFEINMYNEKLHNNQQEQNTLHKEQ

LKIQSDMQKLKELKDNQEILYLKEISLGKSIDTLREKITLSETELNSGFDKLEKKKKDNWEKQETDRKLITEGSRRLSDL

QKMQDDVDSFIYRKIPESLECSESKIKSYEKLLNELFCKKNDIETTINKLKEEMTRQEVRKRELSDNVKLREIQEIINNL

QEQYSNIKEKLNTINYSEIFDEWQNLQSREQTILRQKNIIKGNQEELERTVQQYVQELKKDIYKQAHKNYKSKCIELTVV

EESILNLKEYSKVLDTAMIQYHEERMATVNRIIKQLWKLVYTGTDTTSIEIRTDATEGIGGTKRTYNYKLIQMKHGHEID

MKGRCSAGQKVLASIIIRLALAETFCKDCGILALDEPTTNLDQENADSLANTLATVVKLRSQHQKNFQLIVISHDEKFLF

KLAELSSNKGFYQLYRKQTGYSTVKYCLVENQYHVASNDIKEESDEEVNEHSLRDKLQYEISLGGKIHKEQIYNKKRNIE

DDDFTENRMSKRRYVFQ

>NP_033038.2 DNA repair protein RAD50 [Mus musculus]

MSRIEKMSILGVRSFGIEDKDKQIISFFSPLTILVGPNGAGKTTIIECLKYICTGDFPPGTKGNTFVHDPKVAQETDVRA

QIRLQFRDVNGEMVAVHRSMLCSQKNKKTEFKTLEGVITRMKHGEKVSLSSKCAEIDREMISCLGVSKSVLNNVIFCHQE

DSNWPLSEGKALKQKFDEIFSATRYIKALDTLRQVRQTQGQKVKECQTELKYLKQNKEKACEIRDQITSKEAQLASSQEI

VRSYEDELEPLKNRLKEIEHNLSKIMKLDNEIKALESRKKQMEKDNSELEQKMEKVFQGTDEQLNDLYHNHQRTVREKER

RLVDCQRELEKLNKEARLLNQEKAELLVEQGRLQLQADRHQEHIRARDSLIQSLATHLELDGFERGPFSERQIKNFHELV

KERQEREAKTASQLLSDLTDKEALKQRQLDELRDRKSGLGRTIELKTEILTKKQSELRHVRSELQQLEGSSDRILELDQE

LTKAERELSKAEKNSSIETLKAEVMSLQNEKADLDRSLRKLDQEMEQLNHHTTTRTQMEMLTKDKTDKDEQIRKIKSRHS

DELTSLLGYFPNKKQLEDWLHSKSKEINQTRDRLAKLNKELASAEQNKNHINNELKKKEEQLSSYEDKLFDVCGSQDLES

DLGRLKEEIEKSSKQRAMLAGATAVYSQFITQLTDENQSCCPVCQRVFQTEAELQEVISDLQSKLRLAPDKLKSTESELK

KKERRRDEMLGLVPVRQSIIDLKEKEIPELRNRLQSVNRDIQRLKNDIEEQETLLGTIMPEEESAKVCLTDVTIMERFQM

ELKDVERKIAQQAAKLQGVDLDRTVQQVNQEKQEKQHRLDTVTSKIELNRKLIQDQQEQIQHLKSKTNELKSEKLQIATN

LQRRQQMEEQSVELSTEVQSLNREIKDAKEQISPLETALEKLQQEKEELIHRKHTSNKMAQDKINDIKEKVKNIHGYMKD

IENYIQDGKDDYKKQKETELNGVAVQLNECEKHREKINKDMGTMRQDIDTQKIQERWLQDNLTLRKRRDELKEVEEERKQ

HLKEMGQMQVLQMKNEHQKLEENIDTIKRNHSLALGRQKGYEDEILHFKKELREPQFRDAEEKYREMMIVMRTTELVNKD

LDIYYKTLDQAIMKFHSMKMEEINKIIRDLWRSTYRGQDIEYIEIRSDADENVSASDKRRNYNYRVVMLKGDTALDMRGR

CSAGQKVLASLIIRLALAETFCLNCGILALDEPTTNLDRENIESLAHALVEIIKSRSQQRNFQLLVITHDEDFVELLGRS

EYVEKFYRVKKNMDQCSEIVKCSISSLGSYVH

>NP_005723.2 DNA repair protein RAD50 [Homo sapiens]

MSRIEKMSILGVRSFGIEDKDKQIITFFSPLTILVGPNGAGKTTIIECLKYICTGDFPPGTKGNTFVHDPKVAQETDVRA

QIRLQFRDVNGELIAVQRSMVCTQKSKKTEFKTLEGVITRTKHGEKVSLSSKCAEIDREMISSLGVSKAVLNNVIFCHQE

DSNWPLSEGKALKQKFDEIFSATRYIKALETLRQVRQTQGQKVKEYQMELKYLKQYKEKACEIRDQITSKEAQLTSSKEI

VKSYENELDPLKNRLKEIEHNLSKIMKLDNEIKALDSRKKQMEKDNSELEEKMEKVFQGTDEQLNDLYHNHQRTVREKER

KLVDCHRELEKLNKESRLLNQEKSELLVEQGRLQLQADRHQEHIRARDSLIQSLATQLELDGFERGPFSERQIKNFHKLV

RERQEGEAKTANQLMNDFAEKETLKQKQIDEIRDKKTGLGRIIELKSEILSKKQNELKNVKYELQQLEGSSDRILELDQE

LIKAERELSKAEKNSNVETLKMEVISLQNEKADLDRTLRKLDQEMEQLNHHTTTRTQMEMLTKDKADKDEQIRKIKSRHS

DELTSLLGYFPNKKQLEDWLHSKSKEINQTRDRLAKLNKELASSEQNKNHINNELKRKEEQLSSYEDKLFDVCGSQDFES

DLDRLKEEIEKSSKQRAMLAGATAVYSQFITQLTDENQSCCPVCQRVFQTEAELQEVISDLQSKLRLAPDKLKSTESELK

KKEKRRDEMLGLVPMRQSIIDLKEKEIPELRNKLQNVNRDIQRLKNDIEEQETLLGTIMPEEESAKVCLTDVTIMERFQM

ELKDVERKIAQQAAKLQGIDLDRTVQQVNQEKQEKQHKLDTVSSKIELNRKLIQDQQEQIQHLKSTTNELKSEKLQISTN

LQRRQQLEEQTVELSTEVQSLYREIKDAKEQVSPLETTLEKFQQEKEELINKKNTSNKIAQDKLNDIKEKVKNIHGYMKD

IENYIQDGKDDYKKQKETELNKVIAQLSECEKHKEKINEDMRLMRQDIDTQKIQERWLQDNLTLRKRNEELKEVEEERKQ

HLKEMGQMQVLQMKSEHQKLEENIDNIKRNHNLALGRQKGYEEEIIHFKKELREPQFRDAEEKYREMMIVMRTTELVNKD

LDIYYKTLDQAIMKFHSMKMEEINKIIRDLWRSTYRGQDIEYIEIRSDADENVSASDKRRNYNYRVVMLKGDTALDMRGR

CSAGQKVLASLIIRLALAETFCLNCGIIALDEPTTNLDRENIESLAHALVEIIKSRSQQRNFQLLVITHDEDFVELLGRS

EYVEKFYRIKKNIDQCSEIVKCSVSSLGFNVH

>NP_726199.3 rad50, isoform D [Drosophila melanogaster]

MSSIESLSIQGIRSFGTYADDLQSIKFSSPVTLILGENGCGKTTVVECLKYALTGECPPGSDRGKSFVHDPKIFGLNEVL

AQIKMQVRDRRGAQVSICRTMKVSKKRNKMSFETMDSTINFLTGAGQSKREKQDSLSGRSVDIDVAISDFMGVSKAIINN

VLFCHQEDSSWPLDESKKLKEKFDAIFGITEYNKALDKIIKLRKEAMEELKIKEANIKHVAYLKQEMEVKTLNLQKAQRK

CDAIKAQCSECEEEMKPIEARLVEIRNVEFEIGKYQAQKVEMDTKHKNCKDQISTLTLKIKKPFRGTLDELDQEISNFDQ

RMLEMRQKRTEVEGDLSQIKRSSVAEQEKLGTQDRKHCLAKQRHQSELACRAQLLKRVKEFCRELHIPIDCDLVEQPEKM

GEVLRDIEAMIITKHCEITEIVEQNEKADRSRQVKIDELRIELTKSEQSVTAQEKQRESSKRESETLGVEIKKIETSMQD

LKKLEKEINEVNELYESATKNIDQQAIKDAIARKKASIAENQIQFKKLDEQLTFLGSMAKLVAECSLKQKELDKKNQEVH

RVRSRHSDHFGKLFKEPITCNYRRSMQVVYEKLRREIQELNEKANTQKLKEQSYEIKRKNLISDISRMEKELKDSEELIY

QKCRSTPYDDLLERSKTTISKLQFDHGALKSSEALYKKYIQKMDEEPSCPLCHHNMTSDEACDLTSELTDEIQKLPDNIT

RAEKALKAEQIKYENLLQLKPTILKVKELKDSLPQKKEELKKVEELLGDSVSEYETLIALIGEPTHNMELANSMMGDMSL

LDEALKDSARLTKDLDLQKGQLPASYDSSVSMDDLQAEKSKVSKELETERKELESAQNAVQQQMDALNRLREKKNSLKDR

QIHLREGLQSLPQLKERLEKLNSFLTTVASEISELKAKIQPLKLNLRAAIEEKERLKKSESEKLAQLNSKYNSYKSTDHD

IQRLNKEAEDYAKLDLRNEIKKLDEIIMASKDKLRKLEAEISLKTDELETIKTECSNQQTVERDLKDNRELKQLEDKEAK

LRESCQVLDKQLGNLDFHSVSKEKVNLTKQRDKATVRKGELLGQLGEIHSQVNKLQREIDEPRFKESLKNFRKANYEIEV

TRLCIEDLGQYRLALEWALIQFHSEKMEMINRLIREYWRKIYRGNDIDYIQVKTDEVSSDASADRRKTYNYRVVQSKNYS

EIEMRGRCSAGQRVLASLIIRLALAETFSSNCGVLALDEPTTNLDRANINSLCEALNCIVEERQSQSNFMLIIITHDENF

VSSLGKITSYHRVFRNEECKSVIRRVEAGPSKKALIDQ

>XP_029343905.1 DNA repair protein RAD50 isoform X3 [Acyrthosiphon pisum]

MSTLESISIQGIRSYHPDEKQTLKFYKPLTLILGQNGCGKTTIIECLKYITCSDLPRNANKGGFVWDPKLSDHHTVKGNV

KLSFHDTKDTRVVVSKTLESTQKLATVSTKSLDQTISRKGQSTSHKCADLDDVMCTYLGVSKSILKDVVFCHQEDSNWPL

DEDKKVKEKFDSIFDVGKYDKCIKQIQIEIKATQADKRTCNNNIQHFWKYREEARNKRATLENKKFSFDELQQKLNSIDK

EIIPLEERYKEIMKKEEHFFSLKNKLLTSEGNLNSIRLAIKDLKLLIKLEFKGNDSDLDEELKNFNNNLVQKEKDKYKLN

CLKNDAITNENKIQENMSKEQVNLGRYQSEEKQNLNNINKQNAKIIELGNEMQFNLPDLTEYNRVQVISNLSNNLSSMND

KLESTQKEHREIENKLQSNIDSIRVEKAKLDQEIKIKEKQILENTSEIQKLKKDIDQVRNSAEKLSALQKSKVDIETKLN

ILRGSLNVDDVKKDINKKKDEKYEQNIKLEVIEKEVQKLQLLSIVQAELDTVILNKTGIDNKLKILKNKVNDVLKDLLGF

IPQEKLKFEFTSFLDKLSDTIRCNRKSIAEKQKQLTTLEANYNHTKEKLRTKQIELSNDENALSDMCQGQEYETILKEVN

EKVEELQAQKGSVSTSGHLFRRYIKNLEAQDPSCPLCHRCFGSIDEVTELINDLNMRVHKIPSELETTIAQLETLLKKQS

KLQQLRPTYTRISVLKQTEIPNLKDELERISSNLRKCRDELTKLSDENGKLETNETNAKNIQSDIIMIDSFDNDVKRYSK

EEQRLTTKMTESGSIRNLQEAISERNALKSTINEICNVLEKKQYELNEYTEMLHNLQTQQNKITSDELNIKSKMQDEKSV

IDKLNDLQNLEATISLELDSARETLGPIQEKLDTCINTFEQTKKQQSRKIENNRKEIIILEKKIQEINNLQSSIDNYEKK

EWTLRIKETNNLLNNLNKEKMNYIEEQKNLQKQIDSINEYNANQEVKKIDLENNKKLRQKSKEEIECLSEINKFHEDFGT

MDVNSLLSEKSTNTKQRQALAEEKNKSLGRKQELDNIIRHITEELTAIHFKNAENIYLHEKVQLEILNRVEKDLTKYNKA

LEWAMNRFHKERMQSINTIIKKLWRDIYTGNDIDYIQIKTSSDDKPIQTDTIKKRVFNYRVVQIKNEVELDMRGRCSAGQ

KVLACLIVRMALAETFSKNCGILALDEPTTNLDESNIQSLAESLSEIITRRSSQKSFQLLIITHDPNFLRKLSARDMVDT

YYEVKRSNEGMSIVCEQDIKFLP

>XP_313457.4 AGAP003676-PA [Anopheles gambiae str. PEST]

MSTINKLEIRGIRSFGVDRADVQQINFRSPLTLIVGQNGCGKTTIIECLKYGLTGEVPPGTNRGVGFVHDPKIFNTVESL

GQVKLMVKDFTGNTVTAIRSMKITHKGKNPKFETMDSTITMENAQTKEKVTMTRSRVTDINNEMCDAMGVSKAILNNVIF

CHQEDSCWPLEEPKELKKKFDAIFGTTEYNRVIDKLIKISKEYNEKLKEKQGDLKLLQNVKAQAETKQLQLEDGERNMAR

LTQMIGELEEDIRPIQSKLEQLALVERQYSSLLAKKIEFNSKIKSKHEQSQQLRSKINKLFEGSLPELEMEVRIFQQTSA

SKRSELEDEEAELQRLKLQDRSLQSKLQSIDGQKIELAEKQKRERELQIERGKKIRALGDKLSLSLSDDYGAASVNASLV

EAALGSIRNALKAQEATVQSKGKAFEEEDAQAQKRIDKLRETKATLESDCTSKRKQIEQLDREKLQTAREVAEIERSAET

LKRLIDEIDRLEQEYETQVTAADLPAKRKELAERKLLRDQLQDQLDKLDERISSLDAVAMKEQELTLKEQQCTGREAELR

RLRNKHGDSLRRLFPDRTIESNYKSNLQNLYDELQKEVKTVNEKVRRTQATVTEMETTRKSQKQQLDRLERELADAEEKI

YSACRGNAYEEVLAKLNEKIQKNNMEHGEARSAVVLYQKFIANIDNDRCCPVCEKGLESEDAHEVSGKLGDEIRRLPEKI

ETLERTLKKDRADYDKLLALKPISERLEKQRKELPQLKQQLQDTEKRLSTASDELEEYQLAVSEPTANMQLINAIVGDMS

VLDKLGSELERMKRGVEELRAQLGPNMAEGVTIESLKSEREALRAKFKTERNRTDELQNTIDTKTEKLNNLQARNNQMKS

KKLKLQESVQSLEQKRASVNELGAKIATLEEEFKDAERRLTPVRQQLQQELEQKQRSKEQNNKELQRLRKALEELRWEET

AIVRLSGELDDLAALNLGVELSRLQAKREQTQQEQDQLKRTMDGKAKSIEKLRQDIANQHLQERDLLDNRDLKRLMRETA

DLEAELAALVKSMGEMEVCSVQKERDRLIDLRDELQARRSEMNGKVGELQRQLNELRKELNRSEFRNAVRNYVTTLTESI

VQRKIISDIKKYRDALESALREYHTEKMQEINRTIFSLWRDIYRGNDIDYIRINTVDDGVAERSDKRRAYTYGVVQAKND

VEIDMRGRCSAGQKVLASLIIRLALAETFSSNCGVMALDEPTTNLDRDNIDSLCESLRRIVSEREGGHFLLIVITHDEEF

VTKLEKFENYYRISRNSEGKSVIKEEQL

>EFX76217.1 hypothetical protein DAPPUDRAFT_322547 [Daphnia pulex]

MSRIDQLSITGIRNFSSDSAEVIKFEPPVTLILGKNGSGKTTIIESLKYATTGEVPSGTGRGQSFIHDPNLSTNKISIGS

IKLQFFDEHNNKFIVTRSMEARILKSKMDFKTIDGTISIVQPDGTLKSHKNKNNDLNTFVCNTLGVSKALLNNVLFCHQE

DSNWPLEEAKKVKDKFDDIFNTTRYVKCQDEIRKQMIEVKAKMKDIEKMVDRYKMQKDQAEEFQNDQRRHTERKAILEAE

MQQLQKTKTELDAKLKQSMDQYREAEKTKAEIQRKKVRMEELERNRHDLESDITRVITASVEDLEREIQRFQMTKGEREK

ELNVLQRELQRVDQSGKINAAAQQEQLVQLGKLQSEEEQHNNRIKERDCLLSNLARSYQWPGHSAFLSNQPLSSVQVSQY

QDLLKQTVEQEKHKESVLKQELENEERRLLNSLNKLREECVRLEQDIKAKDTFLNTCQRELSEIEAKLDAMEENSRQMTI

LERQQVEAQTLVDRAKRELNEDELIRNIDRAKSEKVMQEAELDHCRSQLKKVMGQRDIRVKLEMHQKDLSSKRTQERRLM

SRETVEDAIKILVGDNANQDNLLSYYNGRLKSITASLSSKQHNLTKLGQEGATCEANLRNIRSQLDSKQSELSAHQQKLK

NAGSSGAAELENDIARLALEIQDLDDQRGLVHGSEKMYNHFIEKMEKQRAHADCPLCHREFDDADESLVLVDELKGRVEA

MPTKKADLDRKIAEKKSKHGQLLQLRPVAQSIAKLAEAEIPKLESGLKDLESRSSIIQVDLRDLEESIEFLKNEEDIGKK

AHPDIIQLDAVKTDIKKLQSLVDNIQAQLGLSGAEGSLTVEEVQEQVEKAEASYRLTTQSIESMNDRYNGHQNKLTQLQQ

NVNRLMERKLQLNNNLQQRGNLLERKTTLETDIEKARLDVEEWKQNLQPMVAKQLAAQATHVDSQKKRNSLTEQARRKVE

ELLNQLRALASFDKEIEKWSADGKVAQLQHSQQEVERLKVEKLALEQRKMEIQENAKKIELALSNCENEERNLRNNLKLI

VNTTDRQVVKQELDQLIATLETFKLQQLVKEIQRLEEQLERCKIRLGELNGTFQEIEKSIRELEVKLRREEFATAEERYL

DQVKDFEIHQGISEDLANFYNALESALMKFHKERMSVINRMVREMWHSTYKGKDIDYVEIHAEESGALGVNSRRQYNYRV

VMVKNGVEMDMRGRCSAGQKVLASLIIRMALAEAFSSKCGVLALDEPTTNLDEDNIASLSDTILELSNAITRQKRKFQLI

VITHDEKFLERMSRDKRMEKFYRVDRNRFNLSEIRQYDVMGGSQSTNIVKKMLK

>CAF18543.1 RAD50 protein, partial [Gallus gallus]

MAKIEKMSILGVRSFGVEDKDKQIITFFNPLTILVGPNGAGKTTIIECLKYISTGDFPPGTKGNSFVHDPKVANETDVRA

QIRLQFRDVSGELIAVQRSMVCTQKSKKTEFKTLEGVITRTKHGEKVSLSSKCAEIDREMISALGVSKSVLNNVIFCHQE

ESNWPLSEGKALKQKFDEIFSATRYIKALETLRQVRLKQGTKVKECQTELKYLKQNKEKAQEIQDNLANREAQLSASKEN

IKSIESQLDPLKSSLAAVEKNLMEVMSLDNNVKALESRRIQMEKDNQDLQRKMEKVFQGTDEQLKDRYHNHQRTVKEKEK

RLSDCKRELDRASKECQRFNSEKSELLIERGRLQLQADRHQEHIKVRDSLIQALSAQLELDGFEQAPFNDRQIAVFHELL

KERQKSDTEAANQLMREFTQKEAMKQEQIDKIRDRKTGLERSIDLKSDIQNKRLAELKNVKYELCQLEGSSDRIAELDRE

IVKMEHELEKAERNSNVETLEQEVQTLQNEKINLDKVLRRLDQEMEQLNLHTTTITQMEMLKKDKADKEEQIRKVKLRHF

EELTLLLGYFPNKKQLEDWLHGKSTEINETRSRHALLNKQLASAEQQKNYISAELRKKEEQLSNYEAKLFDVCGSQDFDS

NLNKLQDEIEKSSKQRAVLAGATAVYSQFITQLTEENQSCCPVCQRVFQTEAELQDVISDLQSKLRLAPDKLKSTESELK

KREKKRDEMIGLKPIRQTVLELQERDIPDLRNRLQTVNRDFARLKGEIEEQETLLQTVLSEKEGANACLQDITLMERYQT

DIRDVERKIAQQEAKLLGVDLNRTVLQVSQEKQAKKHLWDTVTSKIELNQKMKQDQQNQIQELKSTVNELRAEKLQISSS

VQRRQQLEEQTVELTTEVQSLSREIKEEKEQVFPLDATLEKLQQDKEDLINKRTASNKEIQEKINAIKEKVKDINKYTKE

IENYIQQGKEEYKKQKECELDEVNSQLVACEKQKEKISKEMEIIRQDIDTQKIQERWLEDNLTLRERNKELKGVEDNIKE

LVKKMGEMKVPQLKNEQKHLEEKIEALKRNHHVALGRQRGFEEEIVRFKKELRESQFKDAEEKHREMMIVMRTTELVNKD

LDIYYKALDKAIMTFHSMKMQEINKIIRDLWRSTYRGQDIEYIEIRSDADENVSASDKRRSYNYRVVMIKGDTALDMRGR

CSAGQKVLASLIIRLALAETFCLNCGILALDEPTTNLDRENIESLAHALV

>EAT32997.1 AAEL014748-PA [Aedes aegypti]

MPCLCGMIKIETLRSTYVNSERFRWINTKEDQQNQIRSKIQNLFKGSFHELEIEIQNFKQSKSSKLLELREAESDLSSRK

AQEKVLQSKIQDFESRRIHLNTKRGQEQDLCGDRSKKMVALCDRLKLPAVGDFEAGGADVESALKAIKHGIRSEETAVQT

LSKSHDEADHKAQKAIDKLREDKTKLEADFRMKGQMVADFVREKAKTQSEIAAIQRSAETLKKIVTEIEKLEKDYENQKS

NSNVEGMRRQLAEKKIKREELQAKLDRVEEQISALDAVAAKATELHLKEQQFNAKELEFKRLRNKHTENLKRLFPSKTID

TNYKRNVQTLYDDLQRQIKQLNESMRKSQATVTEMETTRRSQKRELDRVERELTENKEKIYTVCQGNPFEEVLAKLKDKI

SKNNLEHGELRSAEVLYKKYISRIEDDKCCPVCHKGMDGADVQDISSELSDEIRRLPEKIEALERMLKADQKKYDQLLAL

QPYSERIAKQTEEIPKLKEQLEETERKLVQASSDLEECQMSILEPNSSVQLINSILGDMSILDESVRDIDRMKKGIENLK

IEVAVKTPEGTTASLDDIKLEREALRGELRVERISIDAMQNKIDEETERLNNLHQRYNQMKEKKIQLQESVQSLDQKKAK

ELELGEKIASCQREMDETENKLGPVKQNLITEEKSKAKAKEGNRANLNKAQAELEELKRMDAEIVRLCKELDALSRLNLA

EEIQKIKRKLQESNEEMKKIIAAIDEKTYQIDSLKKEVMNQDMIERDYLDNRDLMKLKDETTSLKQGLDALMQSIGDMDA

PNVAQERNKLLEQRDAIQSTKSQTTGQIAELENQSKALKKELDRSEFKNAVKNYLKTYSESVVLKKMISDILKYRNALEW

ALMKYHAEKMEQINRSIYSLWRDIYRGNDIDYIRIKTEDESKPDKVTDKRRQYHYRVVQAKNDVEIDMRGRCSAGQKVLA

SLIIRMALAETFSNNCGVMALDEPTTNLDRENIASLCESLRRIVTERENGNFLLVIITHDEDFVTKLEKFDTYYRVSRDH

DGKSIIKEEQL

>EAT43309.1 AAEL005245-PA [Aedes aegypti]

MPPNKCRMINTKEDQQNQIRSKIQNLFKGSFHELEIEIQNFKQSKSSKLLELREAESDLSSRKAQEKVLQSKIQDFESRR

IHLNTKRGQEQDLCGDRSKKMVALCDRLKLPAVGDFEAGGADVESALKAIKHGIRSEETAVQTLSKSHDEADYKAQKAID

KLREDKTKLEADFRMKGQMVADFVREKAKTQSEIATIQRSAETLKKIVTEIEKLEKDYENQKSNSNVEGMRRQLAEKKIK

HEELQAKLDRVEEQISALDAVAAKATELHLKEQQFNAKESEFKRLRNKHTENLKRLFPSKTIDTNYKRNVQTLYDDLQRQ

IKQLNESMRKSQATVTEMETTRRSQKRELDRIERELTENKEKIYTVCQGNPFEEVLAKLKDKISKNNLEHGELRSAEVLY

KKYISRIEDDKCCPVCHKGMDGADVQDISSELSDEIRRLPEKIEALERMLKADQKKYDQLLALQPYSERIAKQTEEIPKL

KEQLEETERKLVQASSDLEECQMSILEPNSSVQLINSILGDMSILDESVRDIDRMKKGIENLKIEVAVKTPEGTTASLDD

IKLEREALRGELRVERISIDAMQNKIDEETERLNNLHQRYNQMKEKKIQLQESVQSLDQKKAKELELGEKIASCQREMDE

TENKLGPVKQNLITEEKSKAKAKEENRANLNKAQAELEELKRMDAEIVRLCKELDALSRLNLAEEIQKIKRKLQESNEEM

KKIIAAIDEKTYQIDSLKKEVMNQDMIERDYLDNRDLMKLKDETTSLKQGLDALMQSIGDMDAPNVAQERNKLLEQRDAI

QSTKSQTTGQIAELENQSKALKKELDRSEFKNAVKNYLKTYSESVVLKKMISDILKYRNALEWALMKYHAEKMEQINRSI

YSLWRDIYRGNDIDYIRIKTEDESKPDKVTDKRRQYHYRVVQAKNDVEIDMRGRCSAGQKVLASLIIRMALAETFSNNCG

VMALDEPTTNLDRENIASLCESLRRIVTERENGNFLLVIITHDEDFVTKLEKFDTYYRVSRDHDGKSIIKEEQL

>AAK93530.1 SD05424p [Drosophila melanogaster]

MAKLVAECSLKQKELDKKNQEVHRVRSRHSDHFGKLFKEPITCNYRRSMQVVYEKLRREIQELNEKANTQKLKEQSYEIK

RKNLISDISRMEKELKDSEELIYQKCRSTPYDDLLERSKTTISKLQFDHGALKSSEALYKKYIQKMDEEPSCPLCHHNMT

SDEACDLTSELTDEIQKLPDNITRAEKALKAEQIKYENLLQLKPTILKVKELKDSLPQKKEELKKVEELLGDSVSEYETL

IALIGEPTHNMELANSMMGDMSLLDEALKDSARLTKDLDLQKGQLPASYDSSVSMDDLQAEKSKVSKELETERKELESAQ

NAVQQQMDALNRLREKKNSLKDKQIHLREGLQSLPQLKERLEKLNSFLTTVASEISELKAKIQPLKLNLRAAIEEKERLK

KSESEKLAQLNSKYNSYKSTDQDIQRLNKEAEDYAKLDLRNEIKKLDEIIMASKDKLRKLEAEISLKTDELETIKTECSN

QQTVERDLKDNRELKQLEDKEAKLRESCQVLDKQLGNLDFHSVSKEKVNLTKQRDKATVRKGELLGQLGEIHSQVNKLQR

EIDEPRFKESLKNFRKANYEIEVTRLCIEDLGQYRLALEWALIQFHSEKMEMINRLIREYWRKIYRGNDIDYIQVKTDEV

SSDASADRRKTYNYRVVQSKNYSEIEMRGRCSAGQRVLASLIIRLALAETFSSNCGVLALDEPTTNLDRANINSLCEALN

CIVEERQSQSNFMLIIITHDENFVSSLGKITSYHRVFRNEECKSVIRRVEAGPSKKALIDQ

>XP_021710211.1 DNA repair protein RAD50 [Aedes aegypti]

MVALCDRLKLPAVGDFEAGGADVESALKAIKHGIRSEETAVQTLSKSHDEADHKAQKAIDKLREDKTKLEADFRMKGQMV

ADFVREKAKTQSEIAAIQRSAETLKKIVTEIEKLEKDYENQKSNSNVEGMRRQLAEKKIKREELQAKLDRVEEQISALDA

VAAKATELHLKEQQFNAKELEFKRLRNKHTENLKRLFPSKTIDTNYKRNVQTLYDDLQRQIKQLNESMRKSQATVTEMET

TRRSQKRELDRVERELTENKEKIYTVCQGNPFEEVLAKLKDKISKNNLEHGELRSAEVLYKKYISRIEDDKCCPVCHKGM

DGADVQDISSELSDEIRRLPEKIEALERMLKADQKKYDQLLALQPYSERIAKQTEEIPKLKEQLEETERKLVQASSDLEE

CQMSILEPNSSVQLINSILGDMSILDESVRDIDRMKKGIENLKIEVAVKTPEGTTASLDDIKLEREALRGELRVERISID

AMQNKIDEETERLNNLHQRYNQMKEKKIQLQESVQSLDQKKAKELELGEKIASCQREMDETENKLGPVKQNLITEEKSKA

KAKEGNRANLNKAQAELEELKRMDAEIVRLCKELDALSRLNLAEEIQKIKRKLQESNEEMKKIIAAIDEKTYQIDSLKKE

VMNQDMIERDYLDNRDLMKLKDETTSLKQGLDALMQSIGDMDAPNVAQERNKLLEQRDAIQSTKSQTTGQIAELENQSKA

LKKELDRSEFKNAVKNYLKTYSESVVLKKMISDILKYRNALEWALMKYHAEKMEQINRSIYSLWRDIYRGNDIDYIRIKT

EDESKPDKVTDKRRQYHYRVVQAKNDVEIDMRGRCSAGQKVLASLIIRMALAETFSNNCGVMALDEPTTNLDRENIASLC

ESLRRIVTERENDIIASPVTTMESR

>XP_021207651.1 DNA repair protein RAD50 [Bombyx mori]

MAGVKSLAIRGIRSFGPEDTDEQRILFDSPLTLILGQNGCGKTTIIECLRYAITGQMPPGSRNECFVHDAKVNRSTEVLG

QVKLKIVNAKDKQLEVTRSMKVTALAKKKTKFQTLDSFLSTVDESGKTKDVSSRCADLDILMHEELGVSKAILNSVVFCH

QEDSSWPLDEGKKVKERFDEIFDADKYSDCLDRLKKIRKDYAQNLKLLEQEVAHLTEKKQDLDKKKLNVVNVSFSWRISD

LDHSLRSTDELEQNIKNYATTVKAKQKELEDSYKKNTSFNKEEEKIANEKSSNEIEYNKFILLESQNQEKIDKRNEQIVE

TAKIAEIENITEVQTNEEAENATKTILEKVESLKSDHKVQKSQADEEEKKAQGFVDECRVALSGHTQKISSKEGEINKKK

KEITKIEKDITSANQSKDKLELIEKKLLAVEEEHKQAENELNIEECQQEINEDEKAVEQYEQEMDELSQKITKLQKQTAK

LKEKEMIEENLKTKEKQLIVLKNKHKTALMELLGNVPEKDFAVTINKFECEVRNEVETLKKKLKEKQIEITTLEAERKHI

RESLNERRSELTKAEDKIYKACGTQPYDVTLSKYTTTVEKLQDEQNVLQSSMFIINKYKGQLKDNSCCPLCNRGFENESE

VTDLVTQLTTQVMNVPGKLEKVTEDLQRASAKKDEILSMKSLNEKITTLKDTEIPQLEKRIVDVDTQIKTLNETVEEITM

SLIEPEQKMTTAKQIHGDMPMLDRYIQEIKTVTKKFEAVKEQCGDVDSEMTLDVATAKQVDFKQKISTLRNRVKTTQKKL

NAHNKKLQTLYEKKTKAKEELLNTQKKVQDIVNLEESKKQLQSDCERLETELKELKEAITPLEVALKEKIEAKTEVVRRN

RDLIEAGSAYIAKVEVAFNKVKSLDAEIQQHKKKNVPREMEKIQEANDKLMEKQKQIMTDRQVLTKKIDTLKDEIAKQEV

YKRDLDDNLCLRKAQTDITNWQKQDDELNEKLNGINKEELNEKESLIIKQTKLFQQKAECTGALSELKKSLKGFRNELEK

SLSKDIEKKFREKMYELHVTKAIDKDIREYAVALDKCLMEFHREKMENINMIIRELWRKIYRGNDIDYIEIKTEGNLTVE

SERRKYDYRVVQSKNGVEIDMRGRCSAGQKVLACLIIRLALAETFSSRFGILALDEPTTNLDQENIHSLCAALGEIVQER

MMQKNFMFIIITHDKEFIESLGNIDKVTHYYEVSRNDNGKSRVKRIRFM

>AAH73850.1 RAD50 protein, partial [Homo sapiens]

MSRIEKMSILGVRSFGIEDKDKQIITFFSPLTILVGPNGAGKTTIIECLKYICTGDFPPGTKGNTFVHDPKVAQETDVRA

QIRLQFRDVNGELIAVQRSMVCTQKSKKTEFKTLEGVITRTKHGEKVSLSSKCAEIDREMISSLGVSKAVLNNVIFCHQE

DSNWPLSEGKALKQKFDEIFSATRYIKALETLRQVRQTQGQKVKEYQMELKYLKQYKEKACEIRDQITSKEAQLTSSKEI

VKSYENELDPLKNRLKEIEHNLSKIMKLDNEIKALDSRKKQMEKDNSELEEKMEKVFQGTDEQLNDLYHNHQRTVREKER

KLVDCHRELEKLNKESRLLNQEKSELLVEQGRLQLQADRHQEHIRARDSLIQSLATQLELDGFERGPFSERQIKNFHKLV

RERQEGEAKTANQLMNDFAEKETLKQKQIDEIRDKKTGLGRIIELKSEILSKKQNELKNVKYELQQLEGSSDRILELDQE

LIKAERELSKAEKNSNVETLKMEVISLQNEKADLDRTLRKLDQEMEQLNHHTTTRTQMEMLTKDKADKDEQIRKIKSRHS

DELTSLLGYFPNKKQLEDWLHSKSKEINQTRDRLAKLNKELASSEQNKNHINNELKRKEEQLSSYEDKLFDVCGSQDFES

DLDRLKEEIEKSSKQRAMLAGATAVYSQFITQLTDENQSCCPVCQRVFQTEAELQEVISDLQSKLRLAPDKLKSTESELK

KKK

>XP_021712904.1 DNA repair protein RAD50-like [Aedes aegypti]

MKEKKIQLQESVQSLDQKKAKELELLLQSTKKTYQIDSLKKEVMNQDMIERDYLDNRDLMKLKDETTSLKQGLDALMQSI

GDMDAPNVAQERNKLLEQRDAIQSTKSQTTGQIAELENQSKALKKELDRSEFKNAVKNYLKTYSESVVLKKMISDILKYR

NALEWALMKYHAEKMEQINRSIYSLWRDIYRGNDIDYIRIKTEDESKPDKVTDKRRQYHYRVVQAKNDVEIDMRGRCSAG

QKVLASLIIRMALAETFSNNCGVMALDEPTTNLDRENIASLCESLRRIVTERENGNFLLVIITHDEDFVTKLEKFDTYYR

VSRDHDGKSIIKEEQL

>BAE21438.1 unnamed protein product [Mus musculus]

MFSSEHQKLEENIDTIKRNHSLALGRQKGYEDEILHFKKELREPQFRDAEEKYREMMIVMRTTELVNKDLDIYYKTLDQA

IMKFHSMKMEEINKIIRDLWRSTYRGQDIEYIEIRSDADENVSASDKRRNYNYRVVMLKGDTALDMRGRCSAGQKVLASL

IIRLALAETFCLNCGILALDEPTTNLDRENIESLAHALVEIIKSRSQQRNFQLLVITHDEDFVELLGRSEYVEKFYRVKK

NMDQCSEIVKCSISSLGSYVH

>BAF83286.1 unnamed protein product, partial [Homo sapiens]

MSRIEKMSILGVRSFGIEDKDKQIITFFSPLTILVGPNGAGKTTIIECLKYICTGDFPPGTKGNTFVHDPKVAQETDVRA

QIRLQFRDVNGELIAVQRSMVCTQKSKKTEFKTLEGVITRTKHGEKVSLSSKCAEIDREMISSLGVSKAVLNNVIFCHQE

DSNWPLSEGKALKQKFDEIFSATRYIKALETLRQVRQTQGQKVKEYQMELKYLKQYKEKACEIRDQITSKEAQLTSSKEI

VKSYENELDPLKNRLKEIEHNLSKIMKLDNEIKALDSRKKQMEKDNSELEEKMEKVFQGTDEQLNDLYHNHQRTVREKER

KLVDCHRELEKLNKESRLLNQEKSELLVEQGRLQLQADRHQEHIRARDSLIQSLATQLELDGFERGPFSERQIKNFHKLV

RERQEGEAKTANQLMNDFAEKETLKQKQIDEIRDKKTGLGRIIELKSEILSKKQNELKNVKYELQQLEGSSDRILELDQE

LIKAERELSKAEKNSNVETLKMEVISLQNEKADLDRTLRKLDQEMEQLNHHTTTRTQMEMLTKDKADKDEQIRKIKSRHS

DELTSLLGYFPNKKQLEDWLHSKSKEINQTRDRLAKLNKELASSEQNKNHINNEL

>AAH62603.1 RAD50 protein, partial [Homo sapiens]

MSRIEKMSILGVRSFGIEDKDKQIITFFSPLTILVGPNGAGKTTIIECLKYICTGDFPPGTKGNTFVHDPKVAQETDVRA

QIRLQFRDVNGELIAVQRSMVCTQKSKKTEFKTLEGVITRTKHGEKVSLSSKCAEIDREMISSLGVSKAVLNNVIFCHQE

DSNWPLSEGKALKQKFDEIFSATRYIKALETLRQVRQTQGQKVKEYQMELKYLKQYKEKACEIRDQITSKEAQLTSSKEI

VKSYENELDPLKNRLKEIEHNLSKIMKLDNEIKALDSRKKQMEKDNSELEEKMEKVFQGTDEQLNDLYHNHQRTVREKER

KLVDCHRELEKLNKESRLLNQEKSELLVEQGRLQLQADRHQEHIRARDSLIQSLATQLELDGFERGPFSERQIKNFHKLV

RERQEGEAKTANQLMNDFAEKETLKQKQIDEIRDKKTGLGRIIELKSEILSKKQNELKNVKYELQQLEGSSDRILELDQE

LIKAERELSKAEKNSNVETLKMEVISLQNEKADLDRTLRKLDQEMEQLNHHTTTRTQMEMLTKDKADKDEQIRKKKK

>AAH76425.1 Im:6906849 protein, partial [Danio rerio]

MSKIEKMSILGVRSFGVEDKDKQVISFFSPLTVLVGPNGAGKTTIIECLKYITSGDFPPGSKGNTFVHDPKDAHETDVRA

QIRLQFRDVNGDAVAVQRSMQCTQKGKKTEFKTLEGVITRIKHGEKVSLSSKCAEIDREMISSLGVSRAVLNHVIFCHQE

ESNWPLSEGKALKQKFDEIFSATRYIKVLETLRTLRQKQTNTVKSCQMELKYLKQNKDKAQEIRELLSTKETQLASSKES

VNRIEGQIDPLERRLNDIESSLGKVMKLDNDIKALDSRKKQMEDDNRELEEKMEQVFQGSDDQLQDMYQNHQRTVKEKEK

RLVECQRELERAGRECQRMNRIKSELLVEQGRLQLEADRHTQNIKKRDTQVKTLASFLELEGYDRTPLSERQLQSFYRQI

KKKKKKKKK

>AAH58180.1 Rad50 protein, partial [Mus musculus]

MSRIEKMSILGVRSFGIEDKDKQIISFFSPLTILVGPNGAGKTTIIECLKYICTGDFPPGTKGNTFVHDPKVAQETDVRA

QIRLQFRDVNGEMVAVHRSMLCSQKNKKTEFKTLEGVITRMKHGEKVSLSSKCAEIDREMISCLGVSKSVLNNVIFCHQE

DSNWPLSEGKALKQKFDEIFSATRYIKALDTLRQVRQTQVQKVKECQTELKYLKQNKEKACEIRDQITSKEAQLASSQEI

VRSYEDELEPLKNRLKEIEHNLSKIMKLDNEIKALESRKKQMEKDNSELEQKMEKVFQGTDEQLNDLYHNHQRTVREKER

RLVDCQRELEKLNKEARLLNQEKAELLVEQGRLQLQADRHQEHIRARDSLIQSLATHLELDGFERGPFSERQIKNFHELV

KERQEREAKTASQLLSDLTDKEALKQRQLDELRDRKSGLGRTIELKTEILTKKQSELRHVRSELQQLEGSSDRILELDQE

LTKATDKDEQIRKIKSRHSDELTSLLGYFPNKKQLEDWLHSKSKEINQTRDRLAKLNKELASAEQNKNHINNELKKKEEQ

LSSYEDKLFDVCGSQDLESDLGRLKEEIEKSSKQRAMLAGATAVYSQFITQLTDENQSCCPVCQRVFQTEAELQEVISDL

QSKLRLAPDKLKSTESEKKKK

>XP_001661899.2 DNA repair protein RAD50 [Aedes aegypti]

MSTISKLEIRGIRSFGVESGDVQKIKFQSPLTLIVGQNGCGKTTIIECLKYGLTGEVPPGTDRGKAFVHDPKIFSTVESM

GQVKLMVTDFTGNRVTATRSMKVSQKGRGQQPKFETLDSVVTMENVATGEKTTLSRPRAADINNEMCDAMGVSKAIINNV

IFCHQEDSNWPLEEPKELKKKFDAIFGTTEYNRVIEKLIKISKEYNDRQKEKAGDLKLLENIKSQAEVKHLQLQKVDKSR

QDKLAVVDSLENSLKPIYERLDQIAKIERDFSKLKQQEVEFKSK

>EAT36122.1 AAEL011772-PA [Aedes aegypti]

MSTISKLEIRGIRSFGVESGDVQKIKFQSPLTLIVGQNGCGKTTIIECLKYGLTGEVPPGTDRGKAFVHDPKIFSTVESM

GQVKLMVTDFTGNRVTATRSMKVSQKGRGQQPKFETLDSVVTMENVATGEKTTLSRPRAADINNEMCDAMGVSKAIINNV

IFCHQEDSNWPLEEPKELKKKFDAIFGTTEYNRVIEKLIKISKEYNDRQKEKAGDLKLLENIKSQAEVKHLQLQKVDKSR

QDKLAVVDSLENSLKPIYERLDQIAKIERDFSKLKQQEIEFKSK

>ACI97325.1 RAD50, partial [Drosophila melanogaster]

IKFSSPVTLILGENGCGKTTVVECLKYALTGECPPGSDRGKSFVHDPKIFGLNEVLAQIKMQVRDRRGAQVSICRTMKVS

KKRNKMSFETMDSTINFLTGAGQSKREKQDSLSGRSVDIDVAISDFMGVSKAIINNVLFCHQEDSSWPLDESKKLKEKFD

AIFGITEYNKALDKIIKLRKEAMEELKIKEANIKHVAYLKQEMEVKTLNLQKAQRKCDAIKAQCSECEEEMKPIEARLVE

IRNVEFEIGKYQAQKVEMDTKHKNCKDQISTLTLKIKKPFRGTLDELDQEISNFDQRMLEMRQKRTEVEGDLSQIKRSSV

AEQEKLGTQDRKHCLAKQRHQSELACRAQLLKRVKEFCRELHIPIDCDLVEQPEKMGEVLRDIEAMIITKHCEITEIVEQ

NEKADRSXQVKIDELRIELTKSEQSVTAQEKQRESSKRESETLGVEIKKIETSMQDLKKLEKEINEVNELYESATKNIDQ

QAIKDAIARKKASIAENQIQFKKLDEQLTFLGSMAKLVAECSLKQKELDKKNQEVHRVRSRHSDHFGKLFKEPITCNYRR

SMQVVYEKLRREIQELNEKANTQKLKEQSYEIKRKNLINDISRMEKELKDSEELIYQKCRSTPYDDLLERSKA

>XP_021712818.1 DNA repair protein RAD50-like [Aedes aegypti]

MSTISKLEIRGIRSFGVESGDVQKIKFQSPLTLIVGQNGCGKTTIIECLKYGLTGEVPPGTDRGKAFVHDPKIFSTVESM

GQVKLMVTDFTGNRVTATRSMKVSQKGRGQQPKFETLDSVVTMENVATGEKTTLSRPRAADINNEMCDAMGVSKAIINNV

IFCHQEDSNWPLEEPKELKKKFDAIFGTTEYNRVIEKLIKISKEYNDRQKEKAGDLKLLENIKSQAEVKHLQLQKVDKAG

RTNSL

>ACI97330.1 RAD50, partial [Drosophila melanogaster]

IKFSSPVTLILGENGCGKTTVVECLKYALTGECPPGSDRGKSFVHDPKIFGLNEVLAQIKMQVRDRRGAQVSICRTMKVS

KKRNKMSFETMDSTINFLTGAGQSKREKQDSLSGRSVDIDVAISDFMGVSKAIINNVLFCHQEDSSWPLDESKKLKEKFD

AIFGITEYNKALDKIIKLRKEAMDELKIKEANIKVEAYLKQEMEVKTLNLQKAQRKCDAIKAQCSECEEEMKPIEARLVE

IRNVEFEIGKYQAQKVEMDTKHKNCKDQISTLTLKIKKPFRGTLDELDQEISNFDQRMLEMRQKRTEVEGDLSQIKRSSV

AEQEKLGTQDRKHCLAKQRHQSELACRAQLLKRVKEFCRELHIPIDCDLVEQPEKMGEVLRDIEAMIITKHCEITEIVEQ

NEKADRXXQVKIDELRIELTKSEQSVTAQEKQRESSKRESETLGVEIKKIETSMQDLKKLEKEINQVNELYESATKNIDQ

QAIKDAIARKKASIAENQIQFKKLDEQLTFLGSMAKLVAECSLKQKELDKKNQEVHRVRSRHSDHFGKLFKEPITCNYRR

SMQVVYEKLRREIQELNEKANTQKLKEQSYEIKRKNLISDISRMEKELKDSEELIYQKCRSTPYDDLLERSKT

>BAB31030.1 unnamed protein product, partial [Mus musculus]

MSRIEKMSILGVRSFGIEDKDKQIISFFSPLTILVGPNGAGKTTIIECLKYICTGDFPPGTKGNTFVHDPKVAQETDVRA

QIRLQFRDVNGEMVAVQRSMLCSQKNKKTEFKTLEGVITRMKHGEKVSLSSKCAEIDREMISCLGVSKSVLNNVIFCHQE

DSNWPLSEGKALKQKFDEIFSATRYIKALDTLRQVRQTQGQKVKECQTELKYLK

>ACI97320.1 RAD50, partial [Drosophila melanogaster]

IKFSSXVTLILGENGCGKTTVVECLKYALTGECPPGSDRGKSFVHDPKIFGLNEVLAQIKMQVRDRRGAQVSICRTMKVS

KKRNKMSFETMDSTINFLTGAGQSKREKQDSLSGRSVDIDVAISDFMGVSKAIINNVLFCHQEDSSWPLDESKKLKEKFD

AIFGITEYNKALDKIIKLRKEAMEELKIKEANIKHVAYLKQEMEVKTLNLQKAQRKCDAIKAQCSECEEEMKPIEARLVE

IRNVEFEIGKYQAQKVEMDTKHKNCKDQISTLTLKIKKPFRGTLDELDQEISNFDQRMLEMRQKRTEVEGDLSQIKRSSV

AEQEKLGTQDRKHCLAKQRHQSELACRAQLLKRVKEFCRELHIPIDCDLVEQPEKMGEVLRDIEAMIITKHCEITEXXEQ

NEKADRXXQVKIDELRIELTKSEQSVTAQEKQRESSKRESETLGVEIKKIETSMQDLKKLEKEINEVNELYESATKNIDQ

QAIKDAIARKKASIAENQIQFKKLDEQLTFLGSMAKLVAECSLKQKELDKKNQEVHRVRSRHSDHFGKLFKEPITCNYRR

SMQVVYEKLRREIQELNEKANTQKLKEQSYEIKRKNLISDISRMEKELKDSEELIYQKCRSTPYDDLLERSKT

>XP_021712392.1 DNA repair protein RAD50-like [Aedes aegypti]

MTWRSTCEDVASAGQKVLASLIIRMALAETFSNNCGVMALDEPTTNLDRENIASLCESLRRIVTERENGNFLLVIITHDE

DFVTKLEKFDTYYRVSRDHDGKSIIKEEQL

>AAI40006.1 RAD50 protein, partial [Homo sapiens]

MSRIEKMSILGVRSFGIEDKDKQIITFFSPLTILVGPNGAGKTTIIECLKYICTGDFPPGTKGNTFVHDPKVAQETDVRA

QIRLQFRDVNGELIAVQRSMVCTQKSKKKK

>ACI97317.1 RAD50, partial [Drosophila melanogaster]

XXXXXXXXXXXXXXXLXQIXXQXXDRXXAQVSIXXXMKVSKKRNKMSFETMDSTINFLTGAGQSKREKQDSLSGRSVDID

VAISDFMGVSKAIINNVLFCHQEDSSWPLDESKKLKEKFDAIFGITEYNKALDKIIKLRKEAMEELKIKEANIKHVAYLK

QEMEVKTLNLQKAQRKCDAIKAQCSECEEEMKPIEARLVEIRNVEFEIGKYQAXKVEMDTKHKNCKDQISTLTLKIKKPF

RGTLDELDQEISNFDQRMLEMRQKRTEVEGDLSXIKRSSVXEQXXLGTXXXXHXXAKQRHXXXLXXXXXXXXXXXXXXXX

XXXXXXXXXXXXXXXXXXXXXXXXXXXXXXXXXXXXXXXXXXXXXXXXQVKIDELRIELTKSEQSVTAQEKQRESSKRES

ETLGVEIKKIETSMQDLKKLEKEINQINELYESATKNIDQQAIKDAIARKKASIAENQIQFKKLDEQLTFLGSMAKLVAE

CSLKQKELDKKNQEVHRVRSRHSDHFGKLFKEPITCNYRRSMQVVYEKLRREIQELNEKANTQKLKEQSYEIKRKNLISD

ISRMEKELKDSEELIYQKCRSTPYDDLLERSKT

**Table S5. Enolase protein sequences used to build alignment in Supplementary Fig. S3**.

>XP_008198334.1 PREDICTED: enolase [Tribolium castaneum]

MPIKSVAARQIFDSRGNPTVEVDLITDLGLFRAAVPSGASTGVHEALELRDNDKKKYHGKSVQKAIDNINIIIGPELVKS

HLEVTQQTEIDELMIKLDGTENKSKLGANAILGVSLAVAKAGAAKRGLPLYKHIADLCGNKNIILPVPAFNVINGGSHAG

NKLAIQEFMILPTGATSFNEAMKMGSEVYHHLKEIIKKKHGMNATAVGDEGGFSPNLKKAEDALKLIVQAINKAGYKGRI

EIGMDIAASEFFKKGKYDLDFKNKKSNKAKWLTGDKLLKLYKGYVKKFPIVSIEDPFDQDDWKFWHSITSSMDRQIVGDD

LTVTNPKRIETAVEKQACNCLLLKVNQIGSVTEALRAHQLAKANGWGTMVSHRSGETEDTFIADLVVGLSTGQIKTGAPC

RSERLAKYNQILRIEEELGENAKYAGSSFHKPL

>XP_967559.1 PREDICTED: enolase [Tribolium castaneum]

MPMKTIFARQIFDSRGNPTVEVDLITDLGLFRAAVPSGASTGVHEALELRDNDKANYHGKSVQKAINNVNTIIAPELIKS

NLEVTQQTEIDDLMLKLDGTENKSKLGANAILGVSLAVAKAGAAKKGVPLYKHLADLAGNKNIILPVPAFNVINGGSHAG

NKLAMQEFMILPTGAKSFTEAMKMGSEVYHHLKNVIKAQFGLDATAVGDEGGFAPNIQNNEDALNLIIEAIKKAGYEGKI

DIGMDIAASEFYKDGQYDLDFKNPKSDKSKWISGEKLLEMYKGFIKKFPVVSIEDPFDQDDWSSWSAITSSTDIQIVGDD

LTVTNPKRIQTAVEKKACNCLLLKVNQIGSVTESIKAHLLAKSNGWGTMVSHRSGETEDTFIADLVVGLSTGQIKTGAPC

RSERLAKYNQILRIEEELGANAKYAGKAFRKPQ

>XP_975266.1 PREDICTED: enolase [Tribolium castaneum]

MKRTFSSAAKTLVKKIQARQIFDSRGNPTVEVDLVTDLGLFRAALPSGASTGVHEALELRDKDKANYHGKSVFKAIKNVA

LIAPEVIKSQLCVTQQCQIDEIMVNLDGTENKSKLGANAILGVSLAVAKAGAATKKVSLFKHLADLAGNKDIILPVPAFN

VINGGSHAGNKLAMQEFMILPTGAKSFTEAMKMGSEVYHHLKNVIKAQFGLDATAVGDEGGFAPNIQNNEDALNLIIEAI

KKAGYEGKIDIGMDIAASEFYKDGQYDLDFKNPKSDKSKWISGEKLLEMYKGFIKKFPVVSIEDPFDQDDWTSWSAITSS

TDIQIVGDDLTVTNPKRIQTAVEKKACNCLLLKVNQIGSVTESIKAHLLAKSNGWGTMVSHRSGETEDTFIADLVVGLST

GQIKTGAPCRSERLAKYNQILRIEEELGTKAKYAGRNFKFPHK

>XP_317672.2 AGAP007827-PA [Anopheles gambiae str. PEST]

MPFKSIKARQIFDSRGNPTVEVDLVTDLGLFRAAVPSGASTGVHEALELRDNEKANWHGKGVLKAVENINKIIAPAVLSS

NLCVTQQKELDELMLKLDGTENKSKLGANAILGVSLAICKAGAAKKGVPLYKHIADLAGNGNIILPVPAFNVINGGSHAG

NKLAMQEFMILPTGASSFTEAMKIGSEVYHHLKNVIKAKFGLDATAVGDEGGFAPNILENKEALNLIQDAIAKAGYTGKV

EIGMDVAASEFHKEGKYDLDFKNPKSDPSAWLTPDALEQMYQGFIKDFPIVSIEDPFDQDHWDAWSKITANTTIQIVGDD

LTVTNPKRIATAVEKKACNCLLLKVNQIGSVTESINAHLLAKKNGWGTMVSHRSGETEDTFIADLVVGLSTGQIKTGAPC

RSERLAKYNQILRIEEELGAGAKFAGKSFRHPQ

>EAT47221.1 AAEL001668-PA [Aedes aegypti]

MPFKSIKARQIFDSRGNPTVEVDLVTDLGLFRAAVPSGASTGVHEALELRDNVKADWHGKGVLKAVENINKTIAPAVLNS

GLCVTQQKELDELMLKLDGTENKSKLGANAILGVSLAVCKAGAAKKGIPLYKHIAELSGNGNIILPVPAFNVINGGSHAG

NKLAMQEFMILPTGASSFTEAMKIGSEVYHHLKNVIKAKFGLDATAVGDEGGFAPNILENKEALNLIQDAIAKAGYTGKV

EIGMDVAASEFHKDGKYDLDFKNPNSDKSAWLTPDALEGMYQGFIKDFPIVSIEDPFDQDHWDAWAKMTANTSIQIVGDD

LTVTNPKRIATAVEKKACNCLLLKVNQIGTVTESINAHLLAKKNGWGTMVSHRSGETEDTFIADLVVGLSTGQIKTGAPC

RSERLAKYNQILRIEEELGSDAKFAGKNFRHPQ

>BAW35594.1 enolase [Bombyx mori]

MVIKSIKARQIFDSRGNPTVEVDLVTELGLFRAAVPSGASTGVHEALELRDNIKSEYHGKGVLTAIKNINELIAPELTKA

NLEVTQQREIDELMLKLDGTENKSKLGANAILGVSLAVAKAGAAKKNVPLYKHLADLAGNNDIVLPVPAFNVINGGSHAG

NKLAMQEFMIFPTGASTFSEAMRMGSEVYHHLKKIIKEKFGLDSTAVGDEGGFAPNIQNNKDALYLIQDAIQKAGYAGKI

DIGMDVAASEFFKDGKYDLDFKNPDSNPGDYLSSEKLADVYLDFIKDFPMVSIEDPFDQDDWSAWANLTGRTPIQIVGDD

LTVTNPKRIATAVEKKACNCLLLKVNQIGSVTESIDAHLLAKKNGWGTMVSHRSGETEDTFIADLVVGLSTGQIKTGAPC

RSERLAKYNQILRIEEELGVNAKYAGKNFRRPV

>ABH06829.1 enolase, partial [Drosophila melanogaster]

MTIKAIKARQIYDSRGNPTVEVDLTTELGLFRAAVPSGASTGVHEALELRDNDKANYHGKSVLKAVGHVNDTLGPELIKA

NLDVVDQASIDNFMIKLDGTENKSKFGANAILGVSLAVAKAGAAKKGVPLYKHIADLAGNKEIILPVPAFNVINGGSHAG

NKLAMQEFMILPTGATSFTEAMKMGSEVYHHLKNVIKAKFGLDATAVGDEGGFAPNIQSNKEALNLISDAIAKAGYTGKI

EIGMDVAASEFYKDGQYDLDFKNEKSDKSQWLPADKLANLYQEFIKDFPIVSIEDPFDQDHWEAWSNLTGCTAIQIVGDD

LTVTNPKRIATAVEKKACNCLLLKVNQIGTVTESIAAHLLAKKNGWGTMVSHRSGETEDSFIGDLVVGLSTGQIKTGAPC

RSERLAKYNQILRIEEEIGAGVKFAGKSFRKPQ

>EFX83276.1 enolase [Daphnia pulex]

MSIQKIFARMIFDSRGNPTVEVDLTTEKGLFRAAVPSGASTGIHEALELRDDIKTEYHGKGVKKAVSNINDLIAPALLKE

NLQVTDQEAIDNFMIKLDGTENKSKFGANAILGVSLAACKAGATHKGVPLYRHIADLAGTPEIILPTPAFNVINGGSHAG

NKLAMQEFMILPTGASSFTEAMRMGTETYHHLKKVINARFGLDATAVGDEGGFAPNILNNKDALDLITSAIEKAGYTGKI

EIGMDVAASEFHKNGKYDLDFKNPASDPATYLESNKLAELYQEFIKDFPMVSIEDPFDQDDWAAWTSLTGNTPIQIVGDD

LTVTNPKRIQMAVDCKACNCLLLKVNQIGTVTESIAAHKLAKANGWGTMVSHRSGETEDSFIGDLVVGLSTGQIKTGAPC

RSERLAKYNQILRIEEELGAAAKYAGKNFRHPL

>XP_016655758.1 alpha-enolase isoform X1 [Acyrthosiphon pisum]

MMVPFCKYLLNLKQYPDLKLIISRHLQTTNFNRFSCNENMPITKIVARSIFDSRGNPTVEVDLTIDNGPVFRAAVPSGAS

TGIYEALELRDNEKSNYLGKGVNTAVNNINNIIAPALIKAGLIVTEQRAIDDIMLKLDGTPNKAKLGANAILGVSLAVAK

AGAFKKGIPLYKHIADLAGNKEIILPVPAFNVINGGSHAGNKLAMQEFMILPTGASSFTEAMKIGSEVYHTLKNVIKAKF

GLDATAVGDEGGFAPNILENKEGLRLIETAIEKAGYKGKVEIGMDVAASEFFVDGKYDLDFKNKSDSKDKSQIISTEALT

DLYKEFIKEFPMVSIEDPFDQDHWEAWTTLTSSTDIQIVGDDLTVTNPTRIAEAVEKKACNCLLLKVNQIGTVTESIDAH

LLAKKNGWGTMVSHRSGETEDTFIADLVVGLSTGQIKTGAPCRSERLAKYNQILRIEEELGSCAKYAGKNFRNPQAGN

>XP_026300411.1 enolase [Apis mellifera]

MSSIKSIKARQIFDSRGNPTVEVDLVTDDGLFRSEVPSGASTGIHEALELRDNDKSKYHGKSVFKAISNINNTIGPELIK

SNLDVTSQSDIDNFLLKLDGTPNKSNLGANAILGVSLAVCKAGAAKKKKPLYRYIADLAGNTNIILPVPAFNVINGGSHA

GNKLAMQEFMILPTGASSFSDAMKMGTEIYHHLKNGIKSRFGLDATSVGDEGGFAPNILDNKEALNLIIDSIKTAGYDGK

VKIGMDVAASEFYKNGKYDLNFKNEKSDPSTYLDSDSLKNLYLQFIKEFPIVSIEDPFDQDDWSSWTTLTSSTDIQIVGD

DVTVTNPNRIKMAIDKKACNCLLLKVNQIGTVTESINAHKLAKSAGWGTMVSHRSGETEDTFIADLVVGLSTGQIKTGAP

CRSERLAKYNQILRIEEELGSSAKYAGNKFRNPQA

>XP_021701188.1 LOW QUALITY PROTEIN: enolase [Aedes aegypti]

MIRGIISKMPFKSIKARQIFDSRGNPTVEVDLVTDLGLFRAAVPSGASTGVHEALELRDNVKADWHGKGVLKAVENINKT

IAPAVLNSGLCVTQQKELDELMLKLDGTENKSKLGANAILGVSLAVCKAGAAKKGIPLYKHIAELSGNGNIILPVPAFNV

INGGSLPVTSKLAMQEFMILXTGASSFTEAMKIGSEVYHHXRTSSKAKFGLDATAVGDEGGFAPNILENKEALNLIQDAI

AKAGYXGKVEIGMDVAASEFHKDGKHDLDFKNPNSDKSAWLXPDALEGMYQGFIKDFPIVQYXGSFDQDHWDAWAKMTPT

LAFEIVGDDLTVTNPKRIATASEKKACNCLLLKVNQIGTVTESINAHCWAKKNGWGTMVSHRSGETEDTFIADLVVGLST

GQIKTGAPCRSERLAKYNQXLRIEEXLGSDAKFAGKNFRHPQ

>NP_001419.1 alpha-enolase isoform 1 [Homo sapiens]

MSILKIHAREIFDSRGNPTVEVDLFTSKGLFRAAVPSGASTGIYEALELRDNDKTRYMGKGVSKAVEHINKTIAPALVSK

KLNVTEQEKIDKLMIEMDGTENKSKFGANAILGVSLAVCKAGAVEKGVPLYRHIADLAGNSEVILPVPAFNVINGGSHAG

NKLAMQEFMILPVGAANFREAMRIGAEVYHNLKNVIKEKYGKDATNVGDEGGFAPNILENKEGLELLKTAIGKAGYTDKV

VIGMDVAASEFFRSGKYDLDFKSPDDPSRYISPDQLADLYKSFIKDYPVVSIEDPFDQDDWGAWQKFTASAGIQVVGDDL

TVTNPKRIAKAVNEKSCNCLLLKVNQIGSVTESLQACKLAQANGWGVMVSHRSGETEDTFIADLVVGLCTGQIKTGAPCR

SERLAKYNQLLRIEEELGSKAKFAGRNFRNPLAK

>XP_024998255.1 alpha-enolase isoform X1 [Gallus gallus]

MRVADLKRVFKMSILKIHAREIFDSRGNPTVEVDLYTNKGLFRAAVPSGASTGIYEALELRDNDKTRYLGKGVSKAVEHV

NKTIAPALISKNVNVVEQEKIDKLMLEMDGTENKSKFGANAILGVSLAVCKAGAAEKGVPLYRHIADLAGNPEVILPVPA

FNVINGGSHAGNKLAMQEFMILPVGADNFKEAMRIGAEVYHNLKNVIKEKYGKDATNVGDEGGFAPNILENKEALELLKT

AIGKAGYSDKVVIGMDVAASEFYRDGKYDLDFKSPDDPSRYISPDQLADLYKGFVKNYPVVSIEDPFDQDDWAAWKKFTA

SVGIQVVGDDLTVTNPKRIAKAVEEKSCNCLLLKVNQIGSVTESLQACKLAQSNGWGVMVSHRSGETEDTFIADLVVGLC

TGQIKTGAPCRSERLAKYNQLLRIEEELGSKARFAGRNFRNPRIN

>NP_956989.1 enolase 1b, (alpha) [Danio rerio]

MSILKIHAREIFDSRGNPTVEVDLYTERGLFRAAVPSGASTGIYEALELRDNDKSRYLGKGVSKAVENVNQHIAPALIGQ

GIPVVEQEKIDQFMLELDGTENKSRFGANAILGVSLAACKAGAAEKGVPLYRHIADLAGNPEVILPVPAFNVINGGSHAG

NKLAMQEFMILPVGASSFKEAMRIGAEVYHNLKNVIKQKYGQDATNVGDEGGFAPNILENKEALELLKNAISKAGYTDEV

VIGMDVAASEFYRDGQYDLDFKSPDDPDRYISPDELADLYKSFIEDYPVVSIEDPFDQDDWEAWTNFTNSTEIQVVGDDL

TVTNPKRIATAVENEACNCLLLKVNQIGTVTESLQACNMAQSSGWGVMVSHRSGETEDTFIADLVVGLCTGQIKTGAPCR

SERLAKYNQILRIEEELGEKARFAGKNFRNPLN

>NP_001966.1 gamma-enolase [Homo sapiens]

MSIEKIWAREILDSRGNPTVEVDLYTAKGLFRAAVPSGASTGIYEALELRDGDKQRYLGKGVLKAVDHINSTIAPALISS

GLSVVEQEKLDNLMLELDGTENKSKFGANAILGVSLAVCKAGAAERELPLYRHIAQLAGNSDLILPVPAFNVINGGSHAG

NKLAMQEFMILPVGAESFRDAMRLGAEVYHTLKGVIKDKYGKDATNVGDEGGFAPNILENSEALELVKEAIDKAGYTEKI

VIGMDVAASEFYRDGKYDLDFKSPTDPSRYITGDQLGALYQDFVRDYPVVSIEDPFDQDDWAAWSKFTANVGIQIVGDDL

TVTNPKRIERAVEEKACNCLLLKVNQIGSVTEAIQACKLAQENGWGVMVSHRSGETEDTFIADLVVGLCTGQIKTGAPCR

SERLAKYNQLMRIEEELGDEARFAGHNFRNPSVL

>NP_990451.1 alpha-enolase [Gallus gallus]

MSILKIHAREIFDSRGNPTVEVDLYTNKGLFRAAVPSGASTGIYEALELRDNDKTRYLGKGVSKAVEHVNKTIAPALISK

NVNVVEQEKIDKLMLEMDGTENKSKFGANAILGVSLAVCKAGAAEKGVPLYRHIADLAGNPEVILPVPAFNVINGGSHAG

NKLAMQEFMILPVGADTFKEAMRIGAEVYHNLKNVIKEKYGKDATNVGDEGGFAPNILENKEALELLKTAIGKAGYSDKV

VIGMDVAASEFYRDGKYDLDFKSPDDPSRYISPDQLADLYLGFVKNYPVVSIEDPFDQDDWAAWKKFTASVGIQVVGDDL

TVTNPKRIAKAVEEKSCNCLLLKVNQIGSVTESLQACKLAQSNGWGVMVSHRSGETEDTFIADLVVGLCTGQIKTGAPCR

SERLAKYNQLLRIEEELGSKARFAGRNFRNPRIN

>NP_999888.1 beta-enolase [Danio rerio]

MSISKIHAREILDSRGNPTVEVDLYTTKGRFRAAVPSGASTGVHEALELRDGDKTRYLGKGTQKAVDHVNKDIAPKLIEK

KFSVVEQEKIDKFMLELDGTENKSQFGANAILGVSLAVCKAGAAEKGVPLYRHIADLAGNKDVILPVPAFNVINGGSHAG

NKLAMQEFMILPVGAQNFHEAMRIGAEVYHNLKNVIKAKYGKDATNVGDEGGFAPNILENNEALELLKSAIEKAGYPDKI

IIGMDVAASEFFKSGKYDLDFKSPDDPKRHITGEQLGDLYKSFIKNYPVQSIEDPFDQDDWENWSKFTGSVDIQVVGDDL

TVTNPKRIQQACEKKACNCLLLKVNQIGSVTESIQACKLAQSNGWGVMVSHRSGETEDTFIADLVVGLCTGQIKTGAPCR

SERLAKYNQLMRIEEELGDKAKFAGKDFRHPKL

>AAH92869.2 Enolase 3, (beta, muscle) [Danio rerio]

MSISKIHAREILDSRGNPTVEVDLYTTKGRFRAAVPSGASTGVHEALELRDGDKTRYLGKGTQKAVDHVNKDIAPKLIEK

KFSVVEQEKIDKFMLELDGTENKSQFGANAILGVSLAVCKAGAAEKGVPLYRHIADLAGNKDVILPVPAFNVINGGSHAG

NKLAMQEFMILPVGAQNFHEAMRIGAEVYHNLKNVIKAKYGKDATNVGDEGGFAPNILENNEALELLKSAIEKAGYPDKI

IIGMDVAASEFFKSGKYDLDFKSPDDPKRPITGEQLGDLYKSFIKNYPVQSIEDPFDQDDWENWSKFTGSVDIQVVGDDL

TVTNPKRIQQACEKKACNCLLLKVNQIGSVTESIQACKLAQSNGWGVMVSHRSGETEDTFIADLVVGLCTGQIKTGAPCR

SERLAKYNQLMRIEEELGDKAKFAGKDFRHPKL

>1TE6_A Chain A, Gamma Enolase [Homo sapiens]

SIEKIWAREILDSRGNPTVEVDLYTAKGLFRAAVPSGASTGIYEALELRDGDKQRYLGKGVLKAVDHINSTIAPALISSG

LSVVEQEKLDNLMLELDGTENKSKFGANAILGVSLAVCKAGAAERELPLYRHIAQLAGNSDLILPVPAFNVINGGSHAGN

KLAMQEFMILPVGAESFRDAMRLGAEVYHTLKGVIKDKYGKDATNVGDEGGFAPNILENSEALELVKEAIDKAGYTEKIV

IGMDVAASEFYRDGKYDLDFKSPTDPSRYITGDQLGALYQDFVRDYPVVSIEDPFDQDDWAAWSKFTANVGIQIVGDDLT

VTNPKRIERAVEEKACNCLLLKVNQIGSVTEAIQACKLAQENGWGVMVSHRSGETEDTFIADLVVGLCTGQIKTGAPCRS

ERLAKYNQLMRIEEELGDEARFAGHNFRNPSVLHHHHHH

>CAA31512.1 neurone-specific enolase, partial [Homo sapiens]

SIQKIWAREILDSRGNPTVEVDLYTAKGLFRAAVPSGASTGIYEALELRDGDKQRYLGKGVLKAVDHINSTIAPALISSG

LSVVEQEKLDNLMLELDGTENKSKFGANAILGVSLAVCKAGAAERELPLYRHIAQLAGNSDLILPVPAFNVINGGSHAGN

KLAMQEFMILPVGAESFRDAMRLGAEVYHTLKGVIKDKYGKDATNVGDEGGFAPNILENSEALELVKEAIDKAGYTEKMV

IGMDVAASEFYRDGKYDLDFKSPTDPSRYITGDQLGALYQDFVRDYPVVSIEDPFDQDDWAAWSKFTANVGIQIVGDDLT

VTNPKRIERAVEEKACNCLLLKVNQIGSVTEAIQACKLAQENGWGVMVSHRSGETEDTFIADLVVGLCTGQIKTGAPCRS

ERLAKYNQLMRIEEELGDEARFAGHNFRNPSVL

>NP_001342149.1 gamma-enolase isoform 1 [Mus musculus]

MSIEKIWAREILDSRGNPTVEVDLYTAKGLFRAAVPSGASTGIYEALELRDGDKQRYLGKGVLKAVDHINSRIAPALISS

GISVVEQEKLDNLMLELDGTENKSKFGANAILGVSLAVCKAGAAERDLPLYRHIAQLAGNSDLILPVPAFNVINGGSHAG

NKLAMQEFMILPVGAESFRDAMRLGAEVYHTLKGVIKDKYGKDATNVGDEGGFAPNILENSEALELVKEAIDKAGYTEKM

VIGMDVAASEFYRDGKYDLDFKSPADPSRYITGDQLGALYQDFVRNYPVVSIEDPFDQDDWAAWSKFTANVGIQIVGDDL

TVTNPKRIERAVEEKACNCLLLKVNQIGSVTEAIQACKLAQENGWGVMVSHRSGETEDTFIADLVVGLCTGQIKTGAPCR

SERLAKYNQLMRIEEELGDEARFAGHNFRNPSVL

>XP_005162209.1 alpha-enolase isoform X1 [Danio rerio]

MSILKIHAREIFDSRGNPTVEVDLYTKKGLFRAAVPSGASTGIYEALELRDNDKTRYLGKGVKRAVKYVNEFLAPALCNQ

NVSVLEQEKIDKLMLDMDGTDNKSKFGANAILGVSLAVCKAGAAEKGVPLYRHIADLAGNPEVILPVPAFNVINGGSHAG

NKLAMQEFMILPVGASNFKEAMRIGAEVYHNLKNVIKEKYGKDATNVGDEGGFAPNILENKEALELLKNAISKAGYTDKI

VIGMDVAASEFYKGGKYDLDFKSPDDPSRYISPDKLADLYKSFVKDYPVVSIEDPFDQDDWEAWTNFTATTNIQVVGDDL

TVTNPKRIAKAVSDKACNCLLLKVNQIGSVTESLQACKMAQTNGWGVMVSHRSGETEDTFIADLVVGLCTGQIKTGAPCR

SERLAKYNQLLRIEEELGDKARFAGKNFRKPI

>XP_015152319.2 alpha-enolase isoform X3 [Gallus gallus]

MSILKIHAREIFDSRGNPTVEVDLYTNKGLFRAAVPSGASTGIYEALELRDNDKTRYLGKGVSRAVKYVNEFLATALCTQ

NVNVVEQEKIDKLMLEMDGTENKSKFGANAILGVSLAVCKAGAAEKGVPLYRHIADLAGNPEVILPVPAFNVINGGSHAG

NKLAMQEFMILPVGADNFKEAMRIGAEVYHNLKNVIKEKYGKDATNVGDEGGFAPNILENKEALELLKTAIGKAGYSDKV

VIGMDVAASEFYRDGKYDLDFKSPDDPSRYISPDQLADLYKGFVKNYPVVSIEDPFDQDDWAAWKKFTASVGIQVVGDDL

TVTNPKRIAKAVEEKSCNCLLLKVNQIGSVTESLQACKLAQSNGWGVMVSHRSGETEDTFIADLVVGLCTGQIKTGAPCR

SERLAKYNQLLRIEEELGSKARFAGRNFRNPRIN

>NP_001361453.1 beta-enolase isoform 3 [Homo sapiens]

MAVMRTLRAMAMQKIFAREILDSRGNPTVEVDLHTAKGRFRAAVPSGASTGIYEALELRDGDKGRYLGKGVLKAVENINN

TLGPALLQKKLSVVDQEKVDKFMIELDGTENKSKFGANAILGVSLAVCKAGAAEKGVPLYRHIADLAGNPDLILPVPAFN

VINGGSHAGNKLAMQEFMILPVGASSFKEAMRIGAEVYHHLKGVIKAKYGKDATNVGDEGGFAPNILENNEALELLKTAI

QAAGYPDKVVIGMDVAASEFYRNGKYDLDFKSPDDPARHITGEKLGELYKSFIKNYPVVSIEDPFDQDDWATWTSFLSGV

NIQIVGDDLTVTNPKRIAQAVEKKACNCLLLKVNQIGSVTESIQACKLAQSNGWGVMVSHRSGETEDTFIADLVVGLCTG

QIKTGAPCRSERLAKYNQLMRIEEALGDKAIFAGRKFRNPKAK

>NP_001366056.1 alpha-enolase isoform 1 [Mus musculus]

MSILRIHAREIFDSRGNPTVEVDLYTAKGLFRAAVPSGASTGIYEALELRDNDKTRFMGKGVSRPVKYVNEFLAPALCTQ

KVNVVEQEKIDKLMIEMDGTENKSKFGANAILGVSLAVCKAGAVEKGVPLYRHIADLAGNPEVILPVPAFNVINGGSHAG

NKLAMQEFMILPVGASSFREAMRIGAEVYHNLKNVIKEKYGKDATNVGDEGGFAPNILENKEALELLKTAIAKAGYTDQV

VIGMDVAASEFYRSGKYDLDFKSPDDPSRYITPDQLADLYKSFVQNYPVVSIEDPFDQDDWGAWQKFTASAGIQVVGDDL

TVTNPKRIAKAASEKSCNCLLLKVNQIGSVTESLQACKLAQSNGWGVMVSHRSGETEDTFIADLVVGLCTGQIKTGAPCR

SERLAKYNQILRIEEELGSKAKFAGRSFRNPLAK

>AAH17249.1 Enolase 3 (beta, muscle) [Homo sapiens]

MAMQKIFAREILDSRGNPTVEVDLHTAKGRFRAAVPSGASTGIYEALELRDGDKGRYLGKGVLKAVENINSTLGPALLQK

KLSVADQEKVDKFMIELDGTENKSKFGANAILGVSLAVCKAGAAEKGVPLYRHIADLAGNPDLILPVPAFNVINGGSHAG

NKLAMQEFMILPVGASSFKEAMRIGAEVYHHLKGVIKAKYGKDATNVGDEGGFAPNILENNEALELLKTAIQAAGYPDKV

VIGMDVAASEFYRNGKYDLDFKSPDDPARHITGEKLGELYKSFIKNYPVVSIEDPFDQDDWATWTSFLSGVNIQIVGDDL

TVTNPKRIAQAVEKKACNCLLLKVNQIGSVTESIQACKLAQSNGWGVMVSHRSGETEDTFIADLVVGLCTGQIKTGAPCR

SERLAKYNQLMRIEEALGDKAIFAGRKFRNPKAK

>NP_001361452.1 beta-enolase isoform 1 [Homo sapiens]

MAMQKIFAREILDSRGNPTVEVDLHTAKGRFRAAVPSGASTGIYEALELRDGDKGRYLGKGVLKAVENINNTLGPALLQK

KLSVVDQEKVDKFMIELDGTENKSKFGANAILGVSLAVCKAGAAEKGVPLYRHIADLAGNPDLILPVPAFNVINGGSHAG

NKLAMQEFMILPVGASSFKEAMRIGAEVYHHLKGVIKAKYGKDATNVGDEGGFAPNILENNEALELLKTAIQAAGYPDKV

VIGMDVAASEFYRNGKYDLDFKSPDDPARHITGEKLGELYKSFIKNYPVVSIEDPFDQDDWATWTSFLSGVNIQIVGDDL

TVTNPKRIAQAVEKKACNCLLLKVNQIGSVTESIQACKLAQSNGWGVMVSHRSGETEDTFIADLVVGLCTGQIKTGAPCR

SERLAKYNQLMRIEEALGDKAIFAGRKFRNPKAK

>CAA36216.1 muscle-specific enolase [Homo sapiens]

MAMQKIFAREILDSRGNPTVEVDLHTAKGRFRAAVPSGASTGIYEALELRDGDKGRYLGKGVLKAVENINNTLGPALLQK

KLSVADQEKVDKFMIELDGTENKSKFGANAILGVSLAVCKAGAAEKGVPLYRHIADLAGNPDLILPVPAFNVINGGSHAG

NKLAMQEFMILPVGASSFKEAMRIGAEVYHHLKGVIKAKYGKDATNVGDEGGFAPNILENNEALELLKTAIQAAGYPDKV

VIGMDVAASEFYRNGKYDLDFKSPDDPARHITGEKLGELYKSFIKNYPVVSIEDPFDQDDWATWTSFLSGVNIQIVGDDL

TVTNPKRIAQAVEKKACNCLLLKVNQIGSVTESIQACKLAQSNGWGVMVSHRSGETEDTFIADLVVGLCTGQIKTGAPCR

SERLAKYNQLMRIEEALGDKAIFAGRKFRNPKAK

>NP_990207.1 gamma-enolase [Gallus gallus]

MAVERIHAREILDSRGNPTVEVDLYTHKGMFRAAVPSGASTGIYEALELRDNDKSRFLGKGVLQAVDHINSTVAPAIVGS

GLSVVDQEKIDNLMLEMDGTENKSKFGANAILGVSLAVCKAGAAEKDVPLYRHIADLAGNSDLILPVPAFNVINGGSHAG

NKLAMQEFMILPVGAESFRDAMRIGAEVYHNLKSVIKEKYGKDATNVGDEGGFAPNILENSEALELLKEAIDKAGYTDKI

VIGMDVAASEFYRDGKYDLDFKSPDDPSRYISADELGDLYQSFVRAYPVLSIEDPFDQDDWEAWSKFTANVGIQIVGDDL

TVTNPKRIERAVEEKACNCLLLKVNQIGSVTEAIQACKLAQENGWGVMVSHRSGETEDTFIADLVVALCTGQIKTGAPCR

SERLAKYNQLMRIEEELGDEARFAGHNFRNPSVL

>NP_001129534.1 beta-enolase [Mus musculus]

MAMQKIFAREILDSRGNPTVEVDLHTAKGRFRAAVPSGASTGIYEALELRDGDKARYLGKGVLKAVEHINKTLGPALLEK

KLSVVDQEKVDKFMIELDGTENKSKFGANAILGVSLAVCKAGAAEKGVPLYRHIADLAGNPDLVLPVPAFNVINGGSHAG

NKLAMQEFMILPVGASSFKEAMRIGAEVYHHLKGVIKAKYGKDATNVGDEGGFAPNILENNEALELLKTAIQAAGYPDKV

VIGMDVAASEFYRNGKYDLDFKSPDDPARHISGEKLGELYKNFIQNYPVVSIEDPFDQDDWATWTSFLSGVDIQIVGDDL

TVTNPKRIAQAVEKKACNCLLLKVNQIGSVTESIQACKLAQSNGWGVMVSHRSGETEDTFIADLVVGLCTGQIKTGAPCR

SERLAKYNQLMRIEEALGDKAVFAGRKFRNPKAK

>CAJ18401.1 Eno3 [Mus musculus]

MAMQKIFAREILDSRGNPTVEVDLHTAKGRFRAAVPSGASTGIYEALELRDGDKARYLGKGVLKAVEHINKTLGPALLEK

KLSVVDQEKVDKFMIELDGTENKSKFGANAILGVSLAVCKAGAAEKGVPLYRHIADLAGNPDLALPVPAFNVINGGSHAG

NKLAMQEFMILPVGASSFKEAMRIGAEVYHHLKGVIKAKYGKDATNVGDEGGFAPNILENNEALELLKTAIQAAGYPDKV

VIGMDVAASEFYRNGKYDLDFKSPDDPARHISGEKLGELYKNFIQNYPVVSIEDPFDQDDWATWTSFLSGVDIQIVGDDL

TVTNPKRIAQAVEEKACNCLLLKVNQIGSVTESIQACKLAQSNGWGVMVSHRSGETEDTFIADLVVGLCTGQIKTGAPCR

SERLAKYNQLMRIEEALGDKAVFAGRKFRNPKAK

>NP_001003848.1 gamma-enolase [Danio rerio]

MSVVSIIAREILDSRGNPTVEVDLRTDKGLFRAAVPSGASTGIYEALELRDGDKSRYNGKGVLKAVGHINDTLGPAIIAS

EISVVDQEKLDNMMIEMDGTENKSQFGANAILGVSLAICKAGAAEKGAPLYRHIADLAGNTELVLPVPAFNVINGGSHAG

NKLAMQEFMVLPVGAESFRDALRVGAELYQTLKGVIKEKYGQDATNVGDEGGFAPNILENSEALELIKTAIDKAGFTDKV

VIGMDVAASEFYREGKYDLDFKSPPNADRHISSDELLEIYQTFINDFPVVSIEDPFDQDDWPAWTNMTGSVGIQIVGDDL

TVTNPKRIEKAAEDRACNCLLLKVNQIGSVTEAIQACKLAQANGWGVMVSHRSGETEDTFIADLVVGLCTGQIKTGAPCR

SERLAKYNQLMRIEEELADQARFAGHNFRNPSAL

>NP_990450.1 beta-enolase [Gallus gallus]

MSIQKIHAREILDSRGEPTVEVDLHTAKGHFRAAVPSGASTGIHEALEPRDGDKKRFLGKGVLKAVEHINKTIGPALIEK

KISVVEQEKIDKVMIEMDGTENKSKFGANAILGVSLAVCKAGAAEKGVPLYRHIADLAGNTELILPVPAFNVINGGSHAG

NKLAMQEFMVLPVGAASFHDAMRVGAEVYHSLKGVIKAKYGKDATNVGGEGGFAPNILDNHEALELLKAAIAQAGYTDKV

VIGMDVAASEFCRDGRYHLDFKSPPHTKRYITGEQLGEIYRGFIKDYPVVSIEDPFDQDDWEAWKRFVFHVDIQVVGDDL

TVTNPKRIAHGAEQHACNCLLLKVNQIGSVTESIQACKLAQSHGWGVMVSHRSGETEDTFIADLVVGLCTGQIKTGAPCR

SERLAKYNQLMRIEEALGDKAKFAGRKFRNPKAK

>AAA52388.1 gamma enolase, partial [Homo sapiens]

GCGLFRAAVPSGASTGIYEALELRDGDKQRYLGKGVLKAVDHINSTIAPALISSGLSVVEQEKLDNLMLELDGTENKSKF

GANAILGVSLAVCKAGAAERNLPLYRHIAQLAGNSDLILPVPAFNVINGGSHAGNKLAMQEFMILPVGAESFRDAMRLGA

EVYHTLKGVIKDKYGKDATNVGDEGGFAPNILENSEALELVKEAIDKAGYTEKIVIGMDVAASEFYRDGKYDLDFKSPTD

PSRYITGDQLGALYQDFVRDYPVVSIEDPFDQDDWAAWSKFTANVGIQIVGDDLTVTNPKRIERAVEEKACNCLLLKVNQ

IGSVTEAIQACKLAQENGWGVMVSHRSGETEDTFIADLVVGLCTGQIKTGAPCRSERLAKYNQLMRIEEELGDEARFAGH

NFRNPSVL

>ABW39167.1 putative enolase protein, partial [Bombyx mori]

LGLFRAAVPSGASTGVHEALELRDNIKSEYHGKGVLTAIKNINELIAPELTKANLEVTQQREIDELMLKLDGTENKSKLG

ANAILGVSLAVAKAGAAKKNVPLYKHLADLAGNNDIVLPVPAFNVINGGSHAGNKLAMQEFMIFPTGASTFSEAMRMGSE

VYHHLKKIIKEKFGLDSTAVGDEGGFAPNIQNNKDALYLIQDAIQKAGYAGKIDIGMDVAASEFFKDGKYDLDFKNPDSN

PGDYLSSEKLADVYLDFIKDFPMVSIEDPFDQDDWSAWANLTGRTPIQIVGDDLTVTNPKRIATAVEKKACNCLLLKVNQ

IGSVTESIDAHLLAKKNGWGTMVSHRSGETEDTFIADLVVGLSTGQIKTGAPCRSERL

>AAI07495.1 Eno3 protein, partial [Danio rerio]

GLLEKKKKKKKKKYKLRSCRRNAHSLTQGQTPRLTPSRLALRMSISKIHAREILDSRGNPTVEVDLYTTKGRFRAAVPSG

ASTGVHEALELRDGDKTRYLGKGTQKAVCKAGAAEKGVPLYRHIADLAGNKDVILPVPAFNVINGGSHAGNKLAMQEFMI

LPVGAQNFHEAMRIGAEVYHNLKNVIKAKYGKDATNVGDEGGFAPNILENNEALELLKSAIEKAGYPDKIIIGMDVAASE

FFKSGKYDLDFKSPDDPKRHITGEQLGDLYKSFIKNYPVQSIEDPFDQDDWENWSKFTGSVDIQVVGDDLTVTNPKRIQQ

ACEKKTCNCLLLKVNQIGSVTESIQACELAQSNGWGVMVSHRSGETEDTFIADLVVGLCTGQIKTGAPCRSERLAKYNQL

MRIEEELGDKAKFAGKDFRHPKL

>AAA37554.1 muscle-specific enolase beta subunit (EC 4.2.1.11), partial [Mus musculus]

GKGVLKAVEHINKTLGPALLEKKLSVVDQEKVDKFMIELDGTENKSKFGANAILGVSLAVCKAGAAEKGVPLYRHIADLA

GNPDLVLPVPAFNVINGGSHAGNKLAMQEFMILPVGASSFKEAMRIGAEVYHHLKGVIKAKYGKDATNVGDEGGFAPNIL

ENNEALELLKTAIQANAYPDKVVIGMDVAASEFYRNGKYDLDFKSPDDPARHISGEKLGELYKNFIQNYPVVSIEDPFDQ

DDWATWTSFLSGVDIQIVGDDLTVTNPKRIAQAVEKKACNCLLLKVNQIGSVTESIQACKLAQSNGWGVMVSHRSGETED

TFIADLVVGLCTGQIKTGAPCRSERLAKYNQLMRIEEALGDKAVFAGRKFRNPKAK

>AAH56611.1 Eno1 protein [Mus musculus]

MLSLSPHFLSLQKVNVVEQEKIDKLMIEMDGTENKSKFGANAILGVSLAVCKAGAVEKGVPLYRHIADLAGNPEVILPVP

AFNVINGGSHAGNKLAMQEFMILPVGASSFREAMRIGAEVYHNLKNVIKEKYGKDATNVGDEGGFAPNILENKEALELLK

TAIAKAGYTDQVVIGMDVAASEFYRSGKYDLDFKSPDDPSRYITPDQLADLYKSFVQNYPVVSIEDPFDQDDWGAWQKFT

ASAGIQVVGDDLTVTNPKRIAKAASEKSCNCLLLKVNQIGSVTESLQACKLAQSNGWGVMVSHRSGETEDTFIADLVVGL

CTGQIKTGAPCRSERLAKYNQILRIEEELGSKAKFAGRSFRNPLAK

>CAA47179.1 enolase [Homo sapiens]

MSILKIIHARDIFESRGNPTVEVDLYTNKGGLFGRAAVPSGASTGIYEALLELRDNDKTRYMGGKGVSKAVEHIINKTIA

PALISKNVNVVEQDKIDNLMLDMDGSENKSKFGANAILGVSLAVCSNAGATAEKGVPLYRHIADLAGNNPEVILPVPAFN

VINGGSHAGNKLAMQEFMIPPCGADRFNDAIRIGAEVYHNLKNVIKEKYGKDATNVGDEGGFAPNILENKEALELLKTAI

GKAGYSDKVVIGMDVAASEFYRDGKYDLDFNSPDDPSRYISPDQLADLYKGFVLGHAVKNYPVGVSIEDPPFDQDDWGAW

KKLFTGSLVGIQVVGDDLTVTKPEARIAKAVEEVKACNCLLLLKVNQIGSVTESLQACKLAQSNGWGVMPVSHRLSGETE

DTFMADLVVGLCTGQIKTGPTCRSERLAKYNQLLRIEEAEAGSKARFAGRNFRNPRIN

>AAB88178.1 alpha enolase, partial [Homo sapiens]

GTENKSKFGANAILGVSLAVCKAGAVEKGVPLYRHIADLAGNSEVILPVPAFNVINGGSHAGNKLAMQEFMILPVGAANF

REAMRIGAEVYHNLKNVIKEKYGKDATNVGDEGGFAPNILENKEGLELLKTAIGKAGYTDKVVIGMDVAASEFFRSGKYD

LDFKSPDDPSRYISPDQLADLYKSFIKDYPVVSIEDPFDQDDWGAWQKFTASAGIQVVGDDLTVTNPKRIAKAVNEKSCN

CLLLKVNQIGSVTESLQACKLAQANGWGVMVSHRSGETEDTFIADLVVGLCTGQIKTGAPCRSERLAKYNQLLRIEEELG

SKAKFAGRNFRNPLAK

>XP_006562554.1 enolase [Apis mellifera]

MPIQKVKARQIFNSRGDPTLEVDVITDVGLLRSSVPSVLVPNPNQAQELRDGNEAMYHGRSVFRAVDVVNNIIAPQLLKS

KLEACQQTEIDSLLNRLDGTENKSKLGANAILGVSIACCKAGAAKKGLPVYRYIAELAENGDLYVPVPSFNMISGGRHAN

NTLPCQEFMIIPIGAESFADAMKMGMEVYRVLEQKIATAQEIQLPLPVSDEGAFTPLEIEEDREALLLLDESIKDAGYEG

RIKISLDMAASAFYKEGGYDLAFKTEESDPDEYMEAEALKDHYLEYFTEFPSVVSIEDPFDQEDWEGWLTLADQDMQIVA

DDLTAMNIDRIEEAIERQMANSLILRLSQIGTVTETINCAKIARISNWGYIVTACEGETEDNFVADLAVGLSAGQFKAGA

PCRSERTAKYNQILRIEEELGKDAKYAGLNFRNPLAK

>XP_004922096.1 enolase [Bombyx mori]

MPIKLLLARQIFDSTGVPTVEVDMVTELGLFRIGVPSTDSKKIAEATQLRDNNPAQYFGMGVSSAIKNINVIIAPELIKQ

NLEVTMQKEIDQFLISLDGTENRSRLGANAILCVSLVVAKAGAAKKGVPLYRHISDMAGVTTIILPVPHFTILTGGILSS

NGLPFQEYIIMPTGASSFADAMRIGSEIYRYVKNAIANKFSVDCTYVSDSGGFSVPLQSHRDALMFLTDAIKQCGYVGKA

EIAINAAASDMFKDGAYDLEFKNPNSNPQEYMSSDKLAEIYLDNIKEFPVCSIEDGFQFDDWSAWSTLTSRTQAQIFGND

LTQTNLRRVGLATEKKAGNAIGLRLNQAGTFTEVIEAFKLLKSNGFASVVVDRWGDTEDVFLADLAVGLSTGQMKCGAPV

RSERVGKYNQIIRIEEELGALSKYAGKNYRGLN

>AAM88901.1 enolase 3, partial [Danio rerio]

ALELRDGDKSRYNGKGVLKAVGHINDTLGPAIIASEISVVEQEKLDNMMIEMDGTENKSQFGANAILGVSLAICKAGAAE

KGVPLYRHIADLAGNTELVLPVPAFNVINGGSHAGNKLTMQEFMVLPVGAESFRDALRVGAELYQTLKGVIKEKYGQDAT

NVGDEGGFAPNILENSEALELIKTAIDKAGFTDKVVIGMDVAASEFYREGKYDLDFKSPPNADRHISSDELLEIYQTFIN

DFPVVSIEDPFDQDDWPAWTNMTGSVGIQIVGDDLTVTNPKRIEKAAEDRACNCLLLKVNQIGSVTEAIQACKLAQANGW

GVMVSHRSGETEDTFIADLVVG

>ACN51957.1 enolase, partial [Daphnia pulex]

TEYHGKGVKKAVSNINDLIAPALLKENLQVTDQEAIDNFMIKLDGTENKSKFGANAILGVSLAACKAGATHKGVPLYRHI

ADLAGTPEIILPTPAFNVINGGSHAGNKLAMQEFMILPTGASSFTEAMRMGTETYHHLKKVINARFGLDATAVGDEGGFA

PNILNNKDALDLITTAIEKAGYTGKIEIGMDVAASEFHKNGKYDLDFKNPASDPATYLESNKLAELYQEFIKDFPMVSIE

DPFDQDDWAAWTSLTGNTPIQIVGDDLTVTNPKRIQMAVDCKACNCLLLKVNQIGTVTESIAAHKLAKANG

>AAH04325.1 ENO1 protein, partial [Homo sapiens]

LAMQEFMILPVGAANFREAMRIGAEVYHNLKNVIKEKYGKDATNVGDEGGFAPNILENKEGLELLKTAIGKAGYTDKVVI

GMDVAASEFFRSGKYDLDFKSPDDPSRYISPDQLADLYKSFIKDYPVVSIEDPFDQDDWGAWQKFTASAGIQVVGDDLTV

TNPKRIAKAVNEKSCNCLLLKVNQIGSVTESLQACKLAQANGWGVMVSHRSGETEDTFIADLVVGLCTGQIKTGAPCRSE

RLAKYNQLLRIEEELGSKAKFAGRNFRNPLAK

>AUR38828.1 enolase, partial [Aedes aegypti]

PTGASSFTEAMKIGSEVYHHLKNVIKAKFGLDATAVGDEGGFAPNILENKEALNLIQDAIAKAGYTGKVEIGMDVAASEF

HKDGKYDLDFKNPNSDKSAWLTPDALEGMYQGFIKDFPIVSIEDPFDQDHWDAWAKMTANTSIQIVGDDLTVTNPKRIAT

AVEKKACNCLLLKVNQIGTVTESINAHLLAKKNGWGTMVSHRSGETEDTFIADLVVGLSTGQIKTGAPCRSERL

>AAH09018.1 Eno2 protein, partial [Mus musculus]

PLPPPPPPTPPTESAVLEEIPAIMSIEKIWAREILDSRGNPTVEVDLYTAKGLFRAAVPSGASTGIYEALELRDGDKQRY

LGKGVLKAVDHINSRIAPALISSGISVVEQEKLDNLMLELDGTENKSLELVKEAIDKAGYTEKMVIGMDVAASEFYRDGK

YDLDFKSPADPSRYITGDQLGALYQDFVRNYPVVSIEDPFDQDDWAAWSKFTANVGIQIVGDDLTVTNPKRIERAVEEKA

CNCLLLKVNQIGSVTEAIQACKLAQENGWGVMVSHRSGETEDTFIADLVVGLCTGQIKTGAPCRSERLAKYNQLMRIEEE

LGDEARFAGHNFRNPSVL

>BAH72040.1 ACYPI005806 [Acyrthosiphon pisum]

MDVAASEFFVDGKYDLDFKNKSDSKDKSQIISTEALTDLYKEFIKEFPMVSIEDPFDQDHWEAWTTLTASTDIQIVGDDL

TVTNPTRIAEAVEKKACNCLLLKVNQIGTVTESIDAHLLAKKNGWGTMVSHRSGETEDTFIADLVVGLSTGQIKTGAPCR

SERLAKYNQILRIEEELGSCAKYAGKNFRNPQAGN

**Table S6. Genomic fragments derived from *T. castaneum* β2t, Rad50 and enolase genes used in this study. Also the sequences for tTA, P2A and EGFP genes used in the vector construction.**

5' -end cis-regulatory sequences in piggybac-ß2t plasmid:

AATTTACGAAAAACCGATGCATGGATTCAAAACATTTTTTAGGTTAAAAACATTTGCGGGGGCAGAGGGCGCCACTGTGCGAATTTAAATGTAAACAATGGAAATTTAAGTTTTCAAGACTTTTCTAAAGGGCAAATAAGTTTGTTTGAGATTTTAGGCCCAAAATGTGTGCACTTGTGGGTTCTGGACGTAAACTGGACGCTTATTTTTGTTGTAATTAGTTGTCGGTAACGTAGCGCTTTGCTTTACCATCGACGCGAATTTAAAATTCCGAAAGAGGCGGCAGTTTTGAAACAACATCTGCTACATGGTTACGAGTTTGTTTACTCCAATTTTCGGTATTTCTTTAATAAATTTGTTTAACGTTAAAGTAAAATGCGAGAAATAGTCCACATACAAGCAGGTCAATGCGGCAATCAAATAGGAGCAAAGGTAAGCGCGCGAAAAATCCACTCGAGCAGTGAGTATTTGTTTTCAGTTT

3' -end cis-regulatory sequences in piggybac-ß2t plasmid:

CCGATTTTTGAAAATTTGCAAGTTGAACGGTGTATTGGACCGATTTATGATGTTATATTGTATTTTTTGTAATAGATAGTAAATTTTTCAACAAATTTTTCAATTAACTACACTTATAAATTCTAAATTAAATTTCGTGGGCATCAAATTGGTTTTCCTCTCTAGAATAAATAAGGAACTTAATTACTACCCCAAATGAAAAGGCATTGGGTTTTTGATATTTGCGGGTTGTTGCATAAATAAAGTTATAACGAACAACAATCGATGAGGTGAACGTTTGGTTGAATTCCTGGCTTTTGGTTTTGAATTATGCAAGTTCAGGTAATAATGAAAAGATTTAGCAAGAGATAAAGTGAATTTTATTAAAATTCCAGCATGCACCATTGCATAACCCTATTTACAAAATAGTTCTAGATTTAGTACAAGTAGTGAACTTTTTCAAGACAATACAGTATTTACAAATTATTACAGTCTGTTCAATACATTATTTAATTTCTTTTAAAATTTATATAACGAACTAATGAATATACCTAAAAAATAGTAATGCGTTCTAAACTGATAAATACATGCACATCTCTTTATTGTAATCATCTATATAAATATTACTTTTACGCGATTTTAATGAAATACTTGATCCTACGATTTTCTGGATAACAAAAAATCATTGAAAAATACATTGGAACTCGACATATTACCCTAAAAATCTTCCATAAATGACTTAGTTAAATATAATTTATTTTTCTAAATTTTTAGTTTTTATCACAATCGAAGCAGGTATACTATTTTTACAAAATAGATATACTTAGATAATAAATTAAGTACCGAATAACTTAAGTGACTAGATGAATGTTAATAATACACAGATATCATCGAATTAAATGCGACAATAAACCGTTAAATGCAAACGCAATCGAATTTTTGGCAAAGACACCTCTCGATGGAAATAATATCAACTTGAAAAATTTGGCTTAGCACACTATAAACAGTCCGTGGAA

5' -end cis-regulatory sequences in piggybac-Rad50 plasmid:

GGTTTGGGGAGATTTTCCAGTCTCTGTTGCAATTGGTTTTCGGGAATGCGTTGCAGGCGCATACGCTCTATATCCTCCGAACGGCGCTGGTTGACCCTAGCATTTACATAAGGATCAGCAGCAAAATTTGCCTCTACTTCATTGCCCGGAATCACAGCAATCAGATGTCCCTTTCGGTTACGATGGATATTCAGGTGCGAACCGCACACAAAGCTCTCGCCGCACACTCCACACTGATATGGTCGCTCGCCCGTGTGGCGCCGCATATGGATCTTAAGGTCGTTGGACTGCACAAAGCTCTTGCTGCACATTTTGCAGGAGTACGGCCTTTGACCCGTGTGCAATCGCATGTGTCGCGCCAGCTTGTTCTGCGAAATAAACTCGTCGACGGTTCGTAGTGCCCCAACTGGGGTAACCTTTGAGTTCTCTCAGTTGGGGGCGTAGGGGGCCTGCTTAATTAATCAGACTGCGCGCGCGATTCCCACTAATTGTGATACTTAGATAAGCTCTATTTTTCTGTATGTTGTAAGCACTTGCTTCGAAATAAAGTTTTTATATTTTTGTCCGCCTTTTTATTTCAATCAAGTCATTACGAGTCACTCAAATTAAAAGTGTCAGAATGTCTCGTTAATTAGGCGAATGCCTAAGTCAATATTTTTGTTGGTTTACACGCAAAACAAGCGAC

3' -end cis-regulatory sequences in piggybac-Rad50 plasmid:

CCTAGGTTGTCTGAATAAATTGATTTCAAGTGTTTGCTGCGAATCCACGAAAAACCCACACAGGAAAAAACGCACTGAGAAAAAAATCTCATGTTGAAACCACCTGACAGAACACGGGACGATTTTTTTGAGTTCTTTTGTATGTTTTTTTGGAAAGTGAGTATTTTCCTGTGTGAGTTTTTTTCCACGTGGCCTTTTTTGTGGCCTTTTTTCTGTGGGAAAAAAATATTCGTATTCGGCGCCCATTTTGGTAGATGTCGCTCGCGTCGCCCCGTTCGAAAATGGCGTTGTGTGTGAAAAGTGAGTGATGTGTTGACAAAAACCAGCAATTTGAAAAAC

5’ -end cis-regulatory sequences in piggybac-Eno plasmid:

GCTTATCGAATACGCGTACGGGGTTTGGGGAGATTTTCCAGTCTCTGTTGCAATTGGTTTTCGGGAATGCGTTGCAGGCGCATACGCTCTATATCCTCCGAACGGCGCTGGTTGACCCTAGCATTTACATAAGGATCAGCAGCAAAATTTGCCTCTACTTCATTGCCCGGAATCACAGCAATCAGATGTCCCTTTCGGTTACGATGGATATTCAGGTGCGAACCGCACACAAAGCTCTCGCCGCACACTCCACACTGATATGGTCGCTCGCCCGTGTGGCGCCGCATATGGATCTTAAGGTCGTTGGACTGCACAAAGCTCTTGCTGCACATTTTGCAGGAGTACGGCCTTTGACCCGTGTGCAATCGCATGTGTCGCGCCAGCTTGTTCTGCGAAATAAACTCGTCGACGGTTCGTAGTGCCCCAACTGGGGTAACCTTTGAGTTCTCTCAGTTGGGGGCGTAGGGGGCCTGCTTAATTAATCAGACTGCGCGCGCGTTTTTGCGATTTGTTGACTTATCTTCCGTGTTTTGATAAGTTTTTACAATTTTTTGTTTGTAAACACTGTGAGGTTATGTAATAGCGCATTCGCAAAAAAAGTTATCTGTCAATTGTGATTCCGTAAGCGCGTAAGCCCGAAAATTCTTTTAATATAATACGAGTTTTTAATGAAGCACATTTTTAAATCCTGTGTTGTGGGAAAAGGAGCCTATTATAAATATTGCGGTACATTTTAATTAAATACACAAGATGTTTTATACAAAATTTTACGCCGATTGTATGCCACCAGCAATACATATTCCATTTAAGAATTTTTTTACCTTACGAACCCTTTTTAAGATCCGACAGAAATTGGCAAATTTTTTGTAAGCTATAAAAAAACAATTTTGTCGTGTTAAGTTTATTTAAATTTATTGATTTCTAATTTTTTACAACAATCACTAACCAAGTTAATAACTAATTTGTCCAACAAAAATTACGTAAAGGTTTAAAATATAGCCAAGAATAAACGAAGAAAAGATGAAAAAAAAATCAAAAACGAGATTAATTTAAGAAAATTTATAAAAAATAAGCCAAAAAACATCCGAATAATTAATTCGAATAAAGTATTCAATAACTTAAAGAAAGTGTCTTTTTTTCTAAATGATTGTTTAAAACCTAAACCATTTAAGAAATTCAAAAATTGGATTATTTTCGTTTTTTTTAAGCATTTAAGCACTTGATTTCACTAGATCCACCATAAATTGGGACGAGTTTTTATTCATTTTTTCTAAATCAAGTCTCATCCGAAACTTGTAACCTTGTACGTCTTTCACCTTGTCATTCCATCAAATCAAAATTCTGTAAGAAATCTCTTTTTTCTCTTTAAAAACCCTTTCCCTTAATTCCGTAGCAGTGTCCTTGACCCTCTGGGCCCCAAATCCAAATTCCTGTAATGTAAATCTCCTTGACTCGAATAATATGCCCATAAAGAGCGTCGCAGCCCGCCAGATATTCGATTCTCGGGGAAACCCAACAGTGGAAGTAGACTTGATCACTGACTTGGGTTTGTTCCGAGCAGCTGTACCGTCCGGAGCCAGCACTGGTTTGTTTCCTTAAACCCTAATCCCACCCAGAGTAACACAAATTTAGG

3’-end cis-regulatory sequences in piggybac-Eno plasmid:

CCTAGGTAAAGTTTCAGACAAATAAAGCTTTTATTTTTTATCACAAATCGAGTTTTTTTTAATTTTCAGTAAGTGCCCAAACGAATCAATCCAAATAACCAGAAGATTTTTTCAAATATTTATTATTTTTAATGTTTGAGTTCAAGGTAAAATTGCTCCAGTAAACAATTTCTTTTTTTGTTCGTGCAGATAATAATTATTTTTGGGAAACTTTATGGAAGTATGAAAAAGAAGCTGTGGAAATAGTTTTACTTGTAGTAGATAAAAAAATATTAATTAATAAAACTCGCTTATTTAAGAGAATTTCATTTAGCCAACGAACAACATCACAAGACTGGATCTAAAATTTACGTAACTTTCGATTATTTTAAACAATGTTAAAGAAAAAGGGAGTACAATTTAGATTCAAAATTTTTGGTTATCATTTTTTTTTAGAAATTCTCCGAACGCTAATTAAATAACAAGAACAGAAATCTACAAAAAAAATTGAGTTGGTGACAGAAACAATGACAGTGGCATTGTCAATTTTCTGCGCCAGATTTTAGCTCTAATAATTCCTTTCGGAACCATTATTTAAGTGTGATAACCCGGCCGATCTCG

tTA adv seq:

ATGTCTAGACTGGACAAGAGCAAAGTCATAAACTCTGCTCTGGAATTACTCAATGAAGTCGGTATCGAAGGCCTGACGACAAGGAAACTCGCTCAAAAGCTGGGAGTTGAGCAGCCTACCCTGTACTGGCACGTGAAGAACAAGCGGGCCCTGCTCGATGCCCTGGCAATCGAGATGCTGGACAGGCATCATACCCACTTCTGCCCCCTGGAAGGCGAGTCATGGCAAGACTTTCTGCGGAACAACGCCAAGTCATTCCGCTGTGCTCTCCTCTCACATCGCGACGGGGCTAAAGTGCATCTCGGCACCCGCCCAACAGAGAAACAGTACGAAACCCTGGAAAATCAGCTCGCGTTCCTGTGTCAGCAAGGCTTCTCCCTGGAGAACGCACTGTACGCTCTGTCCGCCGTGGGCCACTTTACACTGGGCTGCGTATTGGAGGATCAGGAGCATCAAGTAGCAAAAGAGGAAAGAGAGACACCTACCACCGATTCTATGCCCCCACTTCTGAGACAAGCAATTGAGCTGTTCGACCATCAGGGAGCCGAACCTGCCTTCCTTTTCGGCCTGGAACTAATCATATGTGGCCTGGAGAAACAGCTAAAGTGCGAAAGCGGCGGGCCGGCCGACGCCCTTGACGATTTTGACTTAGACATGCTCCCAGCCGATGCCCTTGACGACTTTGACCTTGATATGCTGCCTGCTGACGCTCTTGACGATTTGGACCTTGACATGCTCCCCGGG

8) P2A sequence:

gccactaacttctccctgttgaaacaagcaggggatgtcgaagagaatcccgggcca

9) EGFP sequence:

atggtgagcaagggcgaggagctgttcaccggggtggtgcccatcctggtcgagctggacggcgacgtaaacggccacaagttcagcgtgtccggcgagggcgagggcgatgccacctacggcaagctgaccctgaagttcatctgcaccaccggcaagctgcccgtgccctggcccaccctcgtgaccaccctgacctacggcgtgcagtgcttcagccgctaccccgaccacatgaagcagcacgacttcttcaagtccgccatgcccgaaggctacgtccaggagcgcaccatcttcttcaaggacgacggcaactacaagacccgcgccgaggtgaagttcgagggcgacaccctggtgaaccgcatcgagctgaagggcatcgacttcaaggaggacggcaacatcctggggcacaagctggagtacaactacaacagccacaacgtctatatcatggccgacaagcagaagaacggcatcaaggtgaacttcaagatccgccacaacatcgaggacggcagcgtgcagctcgccgaccactaccagcagaacacccccatcggcgacggccccgtgctgctgcccgacaaccactacctgagcacccagtccgccctgagcaaagaccccaacgagaagcgcgatcacatggtcctgctggagttcgtgaccgccgccgggatcactctcggcatggacgagctgtacaagtaa
